# Recentrifuge: Robust comparative analysis and contamination removal for metagenomics

**DOI:** 10.1371/journal.pcbi.1006967

**Published:** 2019-04-08

**Authors:** Jose Manuel Martí

**Affiliations:** Institute for Integrative Systems Biology (I^2^SysBio), Valencia, Spain; University of Technology Sydney, AUSTRALIA

## Abstract

Metagenomic sequencing is becoming widespread in biomedical and environmental research, and the pace is increasing even more thanks to nanopore sequencing. With a rising number of samples and data per sample, the challenge of efficiently comparing results within a specimen and between specimens arises. Reagents, laboratory, and host related contaminants complicate such analysis. Contamination is particularly critical in low microbial biomass body sites and environments, where it can comprise most of a sample if not all. Recentrifuge implements a robust method for the removal of negative-control and crossover taxa from the rest of samples. With Recentrifuge, researchers can analyze results from taxonomic classifiers using interactive charts with emphasis on the confidence level of the classifications. In addition to contamination-subtracted samples, Recentrifuge provides shared and exclusive taxa per sample, thus enabling robust contamination removal and comparative analysis in environmental and clinical metagenomics. Regarding the first area, Recentrifuge’s novel approach has already demonstrated its benefits showing that microbiomes of Arctic and Antarctic solar panels display similar taxonomic profiles. In the clinical field, to confirm Recentrifuge’s ability to analyze complex metagenomes, we challenged it with data coming from a metagenomic investigation of RNA in plasma that suffered from critical contamination to the point of preventing any positive conclusion. Recentrifuge provided results that yielded new biological insight into the problem, supporting the growing evidence of a blood microbiota even in healthy individuals, mostly translocated from the gut, the oral cavity, and the genitourinary tract. We also developed a synthetic dataset carefully designed to rate the robust contamination removal algorithm, which demonstrated a significant improvement in specificity while retaining a high sensitivity even in the presence of cross-contaminants. Recentrifuge’s official website is www.recentrifuge.org. The data and source code are anonymously and freely available on GitHub and PyPI. The computing code is licensed under the AGPLv3. The Recentrifuge Wiki is the most extensive and continually-updated source of documentation for Recentrifuge, covering installation, use cases, testing, and other useful topics.

## Author summary

Whether in a clinical or environmental sample, metagenomics can reveal what microorganisms exist and what they do. It is indeed a powerful tool for the study of microbial communities which requires equally powerful methods of analysis. Current challenges in the analysis of metagenomic data include the comparative study of samples, the degree of uncertainty in the results, and the removal of contamination. The scarcer the microbes are in an environment, the more essential it is to have solutions to these issues. Examples of sites with few microbes are not only habitats with low levels of nutrients, but also many body tissues and fluids. Recentrifuge’s novel approach combines statistical, mathematical and computational methods to tackle those challenges with efficiency and robustness: it seamlessly removes diverse contamination, provides a confidence level for every result, and unveils the generalities and specificities in the metagenomic samples.

This is a *PLOS Computational Biology* Software paper.

## Introduction

Studies of microbial communities by metagenomics are becoming more popular in different biological arenas, like environmental, clinical, food and forensic studies [1–3]. New DNA and RNA sequencing technologies are boosting these works by dramatically decreasing the cost per sequenced base. Scientists can now analyze sets of sequences belonging to microbial communities from different sources and times to unravel longitudinal (spatial or temporal) patterns in the microbiota (see S1 Fig for an example model). In shotgun metagenomic sequencing (SMS) studies, researchers extract and purify nucleic acids from each sample, sequence them, and analyze the sequences through a bioinformatics pipeline (see S2 and S3 Figs for detailed examples). With the development of nanopore sequencing, portable and affordable real-time SMS is a reality [4].

### Contamination in metagenomics

In the case of low microbial biomass samples, there is very little native DNA from microbes; the library preparation and sequencing methods will return sequences whose principal source is contamination [5, 6]. Sequencing of RNA requiring additional steps introduces still further biases and artifacts [7], which in case of low microbial biomass studies translates into a severe problem of contamination and spurious taxa detection [8]. The clinical metagenomics community is stressing the importance of negative controls in metagenomics workflows and, recently, raised a fundamental concern about how to subtract the contaminants from the results [9].

From the data science perspective, this is just another instance of the importance of keeping a good *signal-to-noise ratio* [10]. When the *signal* (inherent DNA/RNA, target of the sampling) approaches the order of magnitude of the *noise* (acquired DNA/RNA from contamination and artifacts), particular methods are required to tell them apart.

The roots of contaminating sequences are diverse, as they can be traced back to nucleic acid extraction kits (the *kitome*) [11, 12], reagents and diluents [13, 14], the host [15], and the post-sampling environment [16], where contamination arises from different origins such as airborne particles, crossovers between current samples or DNA remains from past sequencing runs [17]. Variable amounts of DNA from these sources are sequenced simultaneously with native microbial DNA, which could lead to severe bias in magnitudes like abundance and coverage, particularly in low microbial biomass situations [18]. If multiplex sequencing uses simple-indexing, false assignments could be easily beyond acceptable rates [19]. Even the metagenomic reference databases have a non-negligible amount of cross-contamination [15, 17, 20].

Regarding the *kitome*, it varies even within different lots of the same products. For example, the DNeasy PowerSoil Kit (formerly known as PowerSoil DNA Isolation Kit), a product that usually provides significant amounts of DNA and has been widely used, including Earth Microbiome Project and Human Microbiome Project, often yields a background contamination by no means negligible [6]. The lower the biomass in the samples, the more essential it is to collect negative control samples to help in the contamination background assessment because, without them, it would be almost impossible to distinguish inherent microbiota in a specimen —signal— from contamination —noise—.

Assuming that the native and contaminating DNA are accurately separated, the problem of performing a reliable comparison between samples remains. In general, the taxonomic classification engine assigns the reads from a sequencing run to different taxonomic ranks, especially if the method uses a more conservative approach like the lowest common ancestor (LCA) [21]. While LCA drastically reduces the risk of false positives, it usually spreads the taxonomic level of the classifications from the more specific to the more general. Even if the taxonomic classifier does not use the LCA strategy, each read is usually assigned a particular score or confidence level, which should be taken into account by any downstream application as a reliability estimator of the classification.

On top of these difficulties, it is still more challenging to compare samples with very different DNA yields, for instance, low microbial biomass samples versus high biomass ones, because of the different resolution in the taxonomic levels. This sort of problem also arises when the samples, even with DNA yields in the same order of magnitude, have an entirely different microbial structure so that the minority and majority microbes are fundamentally different between them [18]. Finally, a closely related problem emerges in metagenomic bioforensic studies and environmental surveillance, where it is essential to have a method prepared to detect the slightest presence of a particular taxon [3, 22, 23] and provide quantitative results with both precision and accuracy.

### Comparison and validation of metagenomic results

From the beginning, the application of SMS to environmental samples supplied biologists with an insight of microbial communities not obtainable from the sequencing of Bacterial Artificial Chromosome (BAC) clones or 16S rRNA [24, 25]. The scientific community soon underlined the need and challenges of comparative metagenomics [26, 27]. MEGAN [28], one of the first metagenomic data analysis tools, provided in its initial release a very basic comparison of samples, which has improved with an interactive approach in more recent versions [29]. In general, metagenomic classification and assembly software is more intra- than inter-sample oriented [30]. Several tools have tried to fill this gap, starting with CoMet [31], a web-based tool for comparative functional profiling that combines different methods such as multi-dimensional scaling and hierarchical clustering analysis to predict functional differences in a collection of metagenomic samples. Soon after, a different approach appeared with the discovery of the crAssphage thanks to the crAss software [32], which provides reference-independent comparative metagenomics using cross-assembly. The following year, Community-analyzer was released, a tool for visually comparing microbial community structure across microbiomes using correlation-based graphs to infer differences in the samples and predict microbial interactions [33]. In 2014, yet another alternative came, COMMET [34], a piece of software that goes a step further by enabling the combination of numerous metagenomic datasets through a scalable method based on efficient indexing. Two years later, a parallel computation method called Simka was published [35], which performs *de novo* comparative metagenomics by counting k-mers concurrently in multiple datasets.

In 2015, a highly publicized report on the metagenomics of the New York subway suggested that the plague and anthrax pathogens were part of the normal subway microbiome. Soon afterward, several critics arose [36] and, later, reanalysis of the New York subway data with appropriate methods did not detect the pathogens [37]. As a consequence of this and other similar problems involving metagenomic studies, a work directed by Rob Knight [38] emphasized the importance of validation in metagenomic results and issued a tool based on BLAST (Platypus Conquistador). This software confirms the presence or absence of a taxon of interest within SMS datasets by relying on two reference sequence databases: one for inclusions, with the sequences of interest, and the other for exclusions, with any known sequence background. Another BLAST-based method for validating the assignments made by less precise sequence classification programs has been recently announced [22].

The approach of Recentrifuge to increased confidence in the results of taxonomic classification engines follows a dual strategy. Firstly, it accounts for the score level of the classifications in every single step. Secondly, it uses a robust contamination removal algorithm that detects and selectively eliminates various types of contaminants, including crossovers. Recentrifuge directly supports the following high-performance taxonomic classifiers: Centrifuge [7], LMAT [21], CLARK [39], CLARK-S [40], and Kraken [41]. Other classification software is supported through a generic parser. The interactive interface of Recentrifuge enables researchers to analyze the results of those taxonomic classifiers using scored Krona-like charts [42]. In addition to the plots for the raw samples, Recentrifuge generates four different sets of scored charts for each taxonomic level of interest: control-subtracted samples, shared taxa (with and without subtracting the controls), and exclusive taxa per sample. This battery of analysis and plots permits robust comparative analysis of multiple samples in metagenomic studies, especially useful in case of low microbial biomass environments or body sites.

### Recentrifuge’s novel approach

Recentrifuge enables robust contamination removal and score-oriented comparative analysis of multiple samples, especially in low microbial biomass metagenomic studies, where contamination removal is a must.

Just as it is essential to accompany any physical measurement by a statement of the associated uncertainty, it is desirable to attend any read classification with a confidence estimation of the assigned taxon. Recentrifuge reads the score given by a taxonomic classification software to the reads and uses this valuable information to calculate an average confidence level for each taxon in the taxonomic tree associated with the sample analyzed. This value may also be a function of further parameters, such as read quality or length, which is especially useful in case of significant variations in the length of the reads, like in the datasets generated by nanopore sequencers.

Only a few codes, such as Krona [42] and MetaTreeMap [43], are hitherto able to handle a score assigned to the classification nodes. In Recentrifuge, the calculated score propagates to all the downstream analysis and comparisons, including the interface, an interactive framework for a straightforward assessment of the validity of the taxonomic assignments. That is an essential advantage of Recentrifuge over other metagenomic dataset analysis tools.

## Design and implementation

### Scored taxonomic trees

**Fig 1:**
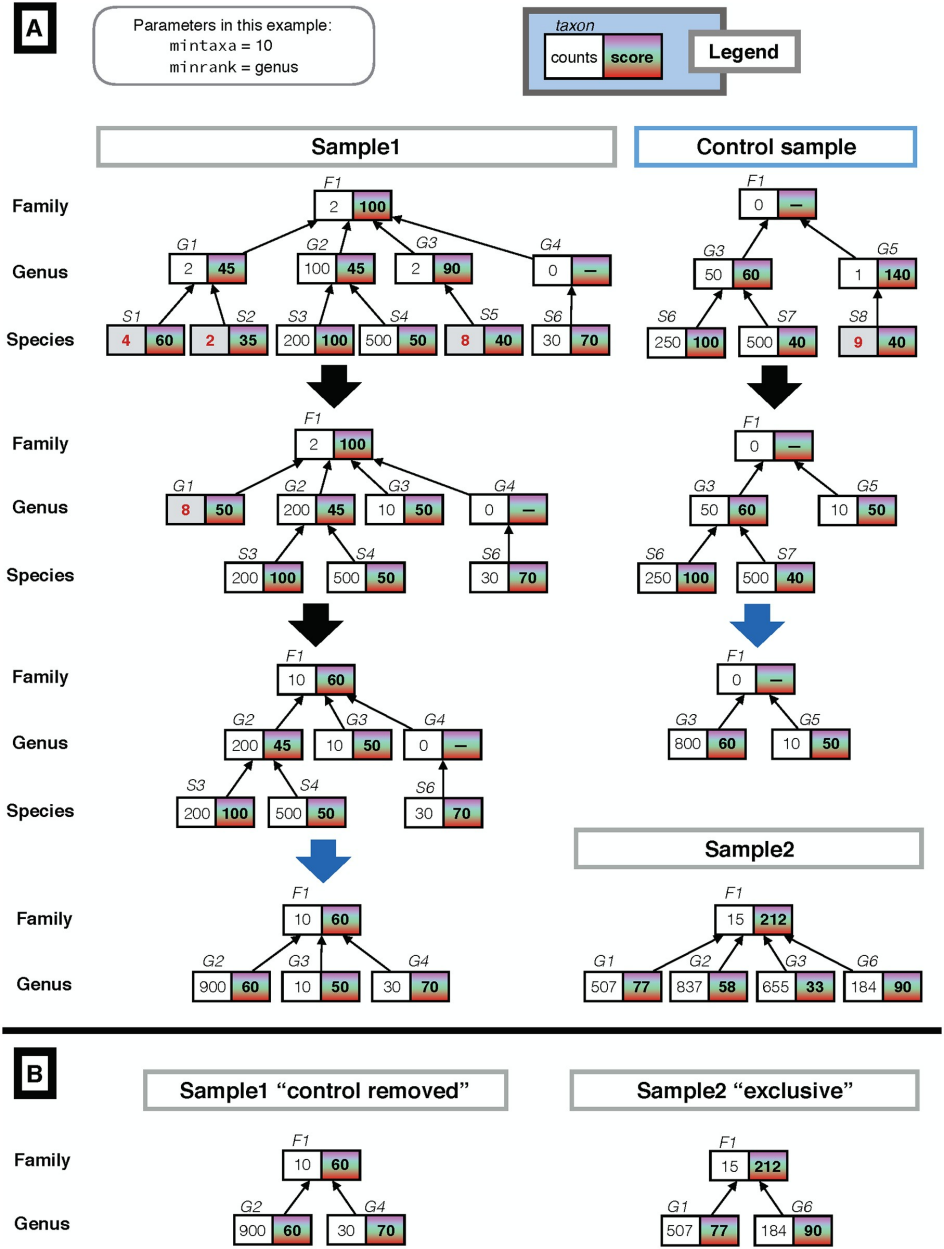
Operating with taxonomic trees. (**A**) Example of the recursive function which ‘folds the tree’ to prepare the taxonomic trees for further operations, with the parameter mintaxa set to 10 (explicitly for this example), and the minimum rank of interest minrank set to ‘genus.’ Initially, their trees show the direct taxonomic classification results. Then, recursively, the leaves of the tree are accumulated in the parent node if their number of assigned reads is under mintaxa (shown in red and bold counts) or if their corresponding taxonomic rank is below minrank. In this ‘folding’ the parent score is updated with a weighted average of its own score and the ones of the descendants that are being accumulated. E.g., after the 1st step, the *G*1 taxon at the sample is updated with *n*_*p*_ = 2 + 4 + 2 = 8 counts and score of $\sigma_{p}=\frac{1}{8}(60\times 4+35\times 2+45\times 2)=50$. As the counts for *G*1 are still under mintaxa, in the 2nd step they are accumulated in *F*1 and its score updated to $\frac{1}{10}(50\times 8+100\times 2)=60$. (**B**) Continuing with the example in (A), at genus level, there are two derived samples: the right one with the control removed from *Sample1*, the left one with the exclusive taxa of *Sample2* (those taxa not present in the rest of samples, in this case, the control and *Sample1*).

For each sample, according to the NCBI Taxonomy [44], Recentrifuge populates a logical taxonomic tree, with the leaves usually belonging to the lower taxonomic levels like species, variety or form. The methods involving trees were implemented as recursive functions for compactness and robustness, making the code less error-prone. One of such methods is essential for understanding the way Recentrifuge prepares samples before any comparison or operation such as control subtraction. It recursively ‘folds the tree’ for any sample if the number of assigned reads to a taxon is under the mintaxa setting (minimum reads assigned to a taxon to exist in its own), or because the taxonomic level of interest is over the assigned taxid (taxonomic identifier). See Fig 1A for a working example of the method in action for two samples. The same procedure applies to the trees of every sample in the dataset. This method does not just ‘prune the tree’, on the contrary, it accumulates the counts *n*_*i*_ of a taxon in the parent ones *n*_*p*_ and recalculates the parent score *σ*_*p*_ as a weighted average taking into account the counts and score of both. In general, the new score of parent taxa, $\sigma_{p}^{\prime }$ is calculated as follows:

$$\begin{array}{c} \sigma_{p}^{\prime }=\frac{1}{n_{p}+\sum_{i}^{D}n_{i}}(\sigma_{p}n_{p}+\sum_{i}^{D}\sigma_{i}n_{i})\forall \left(\sigma_{i},n_{i}\right) \end{array}$$

where 0 < *n_i_* < mintaxa and *D* is the number of descendant taxa that are to be accumulated in the parent one and *σ*_*i*_ their respective scores. This is done recursively until the desired conditions are met. This method is applied, at a given taxonomic level, to the trees of every sample before being compared in search for the shared and exclusive taxa. For a sample, the mintaxa parameter defaults to the nearest integer of the decimal logarithm of the number of reads passing the minimum score threshold (minscore) filter, thus growing with the order of magnitude of the effective size of the sample. However, the user can modify such automatic value for mintaxa and set it independently for control and real samples.

### Derived samples

In addition to the input samples, Recentrifuge includes some sets of derived samples in its output. After parallel calculations for each taxonomic level of interest, it adds hierarchical pie plots for CTRL (control subtracted), but also for EXCLUSIVE, SHARED and SHARED_CONTROL samples, defined below.

Let T mean the set of taxids in the NCBI Taxonomy and *T*_*s*_ the collection of taxids present in a sample *s*. If *R*_*s*_ stands for the set of reads of a sample *s* and *C*_*s*_ for the group of them classifiable, then the taxonomic classification *c* is a function from *C*_*s*_ to T, i.e., $C_{s}\overset{c}{\to }T$, where *C*_*s*_ ⊆ *R*_*s*_ and $c\left[C_{s}\right]=T_{s}\subseteq T$. The set *L* of the 32 − 1 different taxonomic levels used in the NCBI Taxonomy (see S5 Fig) [44] is ordered in accordance with the taxonomy, so (*L*, <) is a strictly ordered set, since *form* < *variety* < *subspecies* < ⋯ < *domain*. Then, $T_{s}=T_{s}^{form}\cup \cdots \cup T_{s}^{domain}=\cup^{L}T_{s}^{l}$, where $T_{s}^{l}$ represents the collection of taxa belonging to a sample *s* for a particular taxonomic rank or level *l*. Related with this, we can write as $T_{s}^{\to l}$ the taxa of the sample *s* for a taxonomic level *l* once we have applied the ‘tree folding’ to such level *l* detailed in the previous subsection (and in Fig 1A).

For a taxonomic rank *k* of interest, in a series of *S* samples where there are *N* < *S* negative controls, Recentrifuge computes the sets of taxa in the derived samples CTRL $\left({}^{\text{CTRL}}T_{s}^{k}\right)$, EXCLUSIVE $\left({}^{\text{EXCL}}T_{s}^{k}\right)$, SHARED (^SHARED^ *T*^*k*^) and SHARED_CONTROL (^SHARED_CTRL^ *T*^*k*^) as:

$$\begin{array}{ccc} {}^{\text{CTRL}}T_{s}^{k} & = & T_{s}^{\to k}\;\;\backslash \;\;\overset{N}{\underset{n}{\cup }}T_{n}^{\to k} \end{array}$$

$$\begin{array}{ccc} {}^{\text{EXCL}}T_{s}^{k} & = & T_{s}^{\to k}\;\;\backslash \;\;\overset{S}{\underset{m\neq s}{\cup }}T_{m}^{\to k} \end{array}$$

$$\begin{array}{ccc} {}^{\text{SHARED}}T^{k} & = & \overset{S}{\underset{m}{\cap }}T_{m}^{\to k} \end{array}$$

$$\begin{array}{ccc} {}^{\text{SHARED}\_\text{CTRL}}T^{k} & = & \overset{S}{\underset{m>N}{\cap }}T_{m}^{\to k}\;\;\backslash \;\;\overset{N}{\underset{n}{\cup }}T_{n}^{\to k} \end{array}$$

Please see Fig 1B for examples. Finally, Recentrifuge generates in parallel a set of SUMMARY samples condensing the results for all the taxonomic levels of interest.

### Robust contamination removal

**Fig 2:**
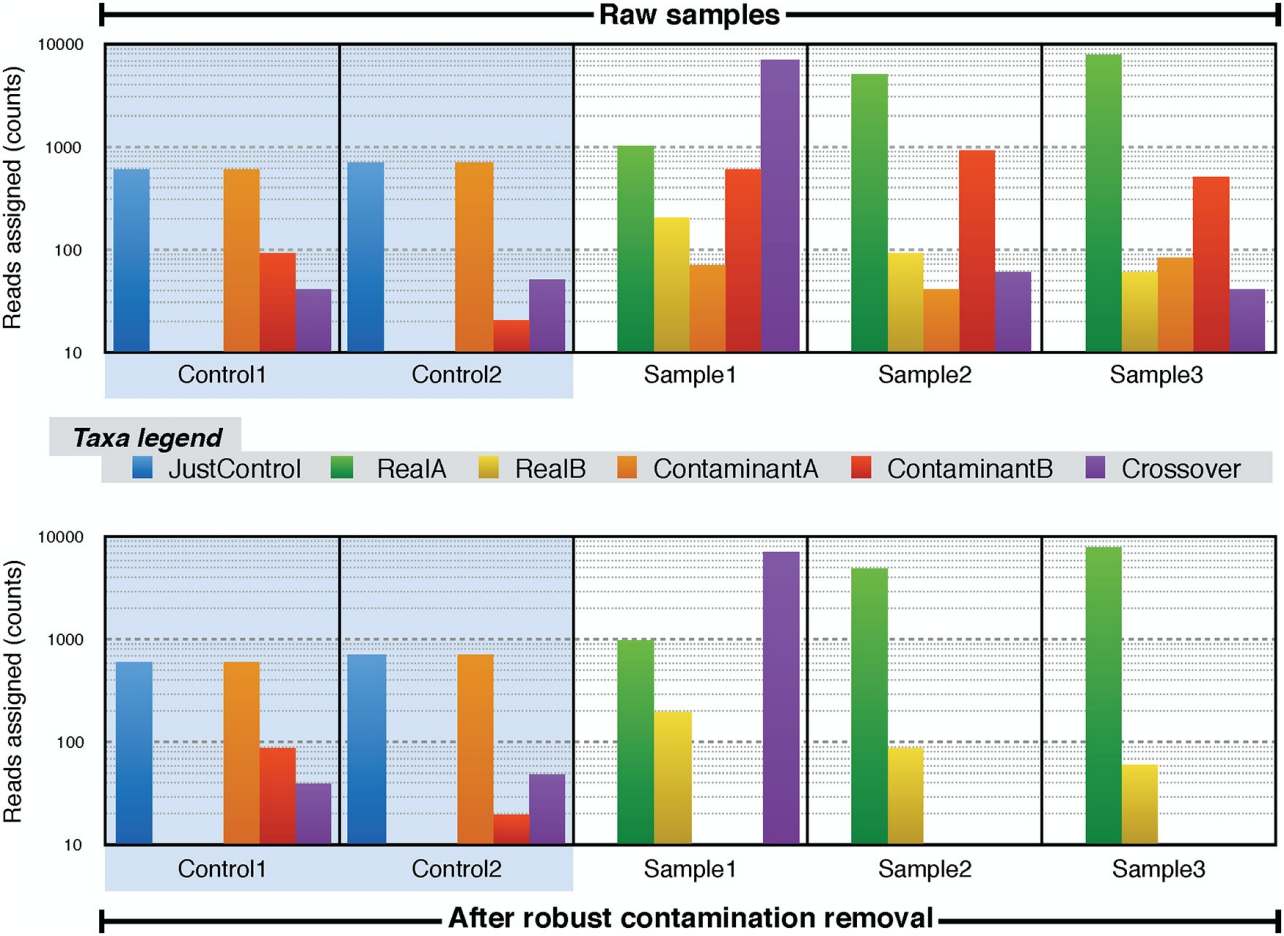
Robust contamination removal. This is a hypothetic example with 5 samples and 6 dominant taxa to illustrate how the algorithm works. The top and bottom part of the figure shows the absolute frequency of reads assigned to the taxa before and after the contamination removal, respectively. There are two control samples, not modified throughout the process. In the rest of specimens, the general contaminants (those taxa present in the controls and other samples, like *ContaminantA* and *ContaminantB*) are removed, except in case of crossover contamination: those taxa are kept in the source sample or samples (Sample1 here) and removed from other real samples (Sample2 and Sample3 in this example). The algorithm parameter *ξ* is set to 2.

For a taxonomic rank *k*, after the ‘tree folding’ procedure detailed above, the contamination removal algorithm retrieves the set of candidates ${\bar{T}}_{s}^{\to k}$ to contaminant taxa from the *N* < *S* control samples. Depending on the relative frequency (*f*_*i*_ = *n*_*i*_/∑_*i*_ *n*_*i*_) of these taxa in the control samples and if they are also present in other specimens, the algorithm classifies them in contamination level groups: critical, severe, mild, and other. Except for the latter group, the contaminants are removed from non-control samples. Then, Recentrifuge checks any taxon in the ‘other contaminants’ group for crossover contamination so that it eliminates any taxon marked as a crossover from every sample except the one or ones selected as the source of the pollution. In detail, the algorithm removes any taxon $t_{s}^{k}\in {\bar{T}}_{s}^{\to k}$ from a non-control sample unless it passes the robust crossover check: a statistical test screening for overall outliers and an order of magnitude test against the control samples. See Fig 2 for an example of this procedure.

The robust crossover tests are defined as follows:

$$\begin{array}{ccc} \text{Outliers}\;\text{statistic}\;\text{test}\;(t_{s}^{k}) & : & f_{t_{s}^{k}}>\mathrm{median}\{f_{t_{1}^{k}},\ldots ,f_{t_{S}^{k}}\}+\delta \;Q_{n} \end{array}$$

$$\begin{array}{ccc} \text{Order}\;\text{of}\;\text{magnitude}\;\text{test}\;(t_{s}^{k}) & : & f_{t_{s}^{k}}>10^{\xi }\max\{f_{t_{1}^{k}},f_{t_{2}^{k}},\ldots ,f_{t_{N}^{k}}\} \end{array}$$

where *Q*_*n*_ [45] is a scale estimator to be discussed below, and *δ* and *ξ* are constant parameters of the robust contamination removal algorithm. The parameter *δ* is an outliers cutoff factor, while *ξ* is setting the difference in order of magnitude between the relative frequency of the candidate to crossover contaminator in the sample *s* and the greatest of such values among the control samples. In Recentrifuge, *δ* typically ranges from 3 to 5, and *ξ* from 2 to 3.

*Q*_*n*_ is the chosen scale estimator for screening the data for outliers because of its remarkably general robustness and other advantages compared to other estimators [45, 46], like the MAD (median absolute deviation) or the *k*-step *M*-estimators. It has a 50% breakpoint point, a smooth influence function, very high asymptotic efficiency at Gaussian distributions and is suitable for asymmetric distributions, which is our case, all at a reasonable computational complexity, as low as *O*(*n*) for space and *O*(*n* log *n*) for time. So, here:

Qn=d{|ftik-ftjk|i<j≤S}(m):m=(S2+12)=Γ(S2+2)2Γ(S2)=S4(S2+1)

where *d* = 3.4760 is a constant selected for asymmetric non-gaussian models similar to the negative exponential distribution, *m* refers to the *m*th order statistics of the pairwise distances and Γ is the Gamma function.

### Parallel computation

**Fig 3:**
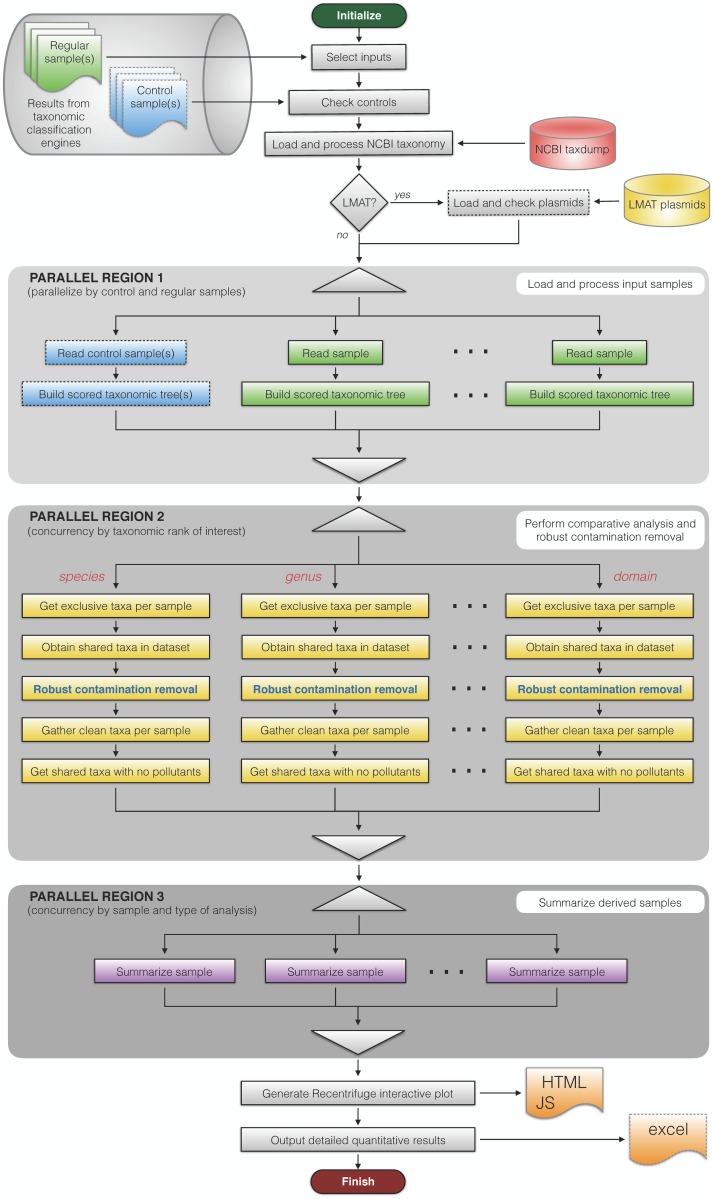
Recentrifuge’s flowchart. The three parallel regions in the code are delimited and labeled. The dashed lines indicate data or steps that are optional. For example, Recentrifuge loads and checks plasmids only in case of LMAT samples, and if the plasmids file of LMAT is present in the file system.

Recentrifuge is a metagenomics analysis software with two different main parts: the computing kernel, implemented and parallelized from scratch using Python, and the interactive interface, based on interactive hierarchical pie charts by extending the Krona [42] 2.0 JavaScript library developed at the Battelle National Biodefense Institute. Recentrifuge’s novel approach combines robust statistics, arithmetic of scored taxonomic trees, and concurrent computational algorithms to achieve its goals. Fig 3 is a flow diagram of Recentrifuge that clearly shows three parallel regions in the code. In each of them, the work divides into concurrent processes attending to different variables: control and regular samples in the first region, the taxonomic ranks in the second, and the specimen along with the type of analysis in the last parallel region, which summarizes the results.

### Components design and implementation

**Fig 4:**
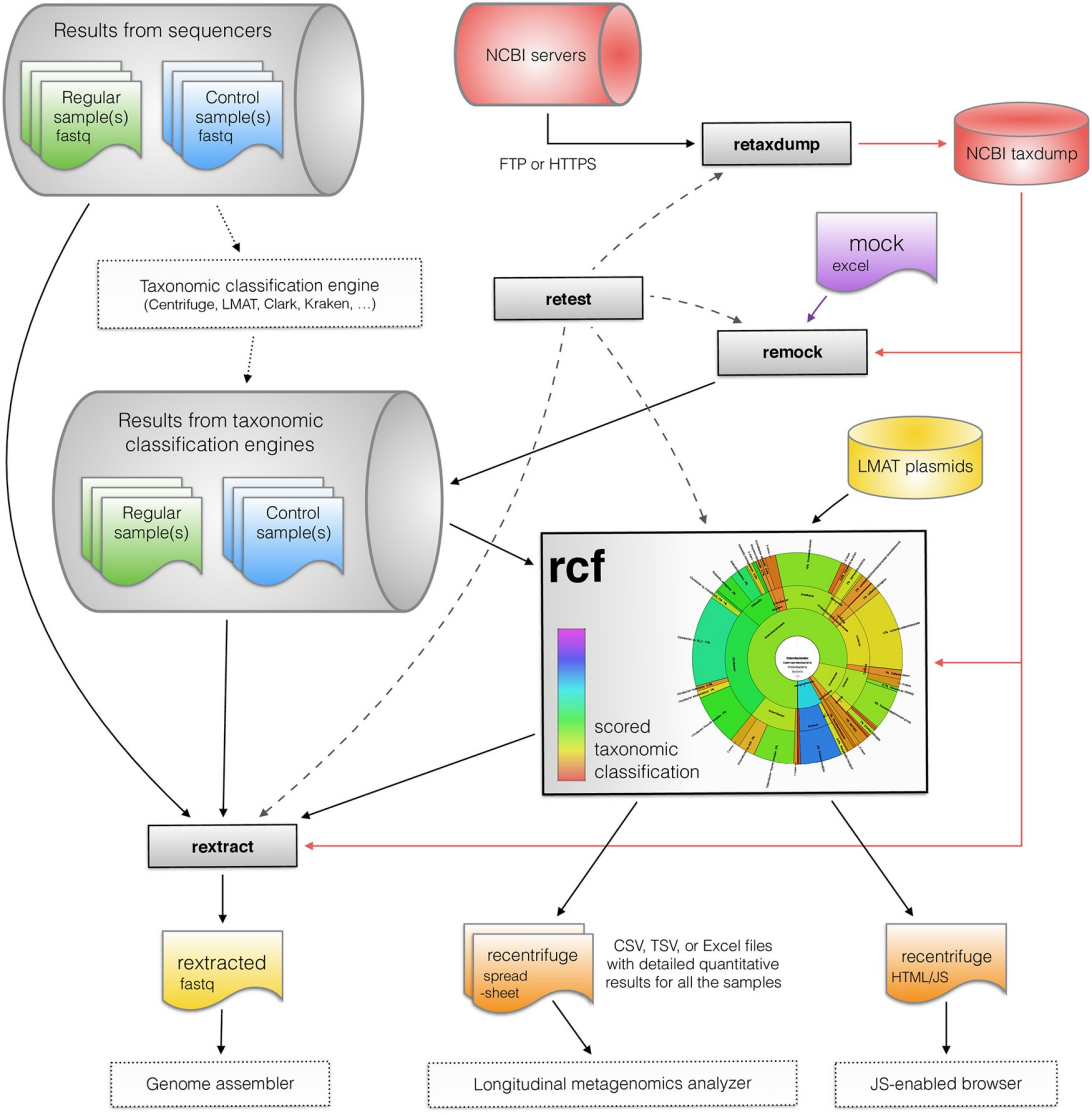
Outline of the Recentrifuge package with its ecosystem and main data flows. Recentrifuge (*rcf*) accepts output files from diverse taxonomic classifiers such as Centrifuge [7], LMAT [21], CLARK [39], CLARK-S [40], Kraken [41], and others, enabling a robust taxonomic analysis for metagenomics. Recentrifuge is also supporting LMAT plasmids assignment system [15]. The additional output of Recentrifuge to different text field formats enable further longitudinal (time or space) series analysis, for example, using *Dynomics* (in development). The NCBI Taxonomy dump databases [44] are easily retrieved using *Retaxdump*. *Rextract* utility extracts a subset of reads of interest from single or paired-ends FASTQ input files, which can be used in any downstream application, like genome assembling and visualization. *Remock* easily creates mock Centrifuge samples, useful for code validation but also for including previously known contaminants. *Retest* is the script in charge of testing (denoted by dashed lines) the other components of the package. The dotted lines indicate software and procedures beyond Recentrifuge.

In any SMS study with related samples, including negative controls, Recentrifuge generates four additional sets of scored charts: the samples with the contamination subtracted, the exclusive taxa per sample, and the shared taxa with and without control taxa subtracted (see S4 Fig). Fig 4 summarizes the package context and data flows. Recentrifuge straightforwardly accepts output files from various taxonomic classifiers, thus enabling a scored-oriented taxonomic visualization for metagenomics. Recentrifuge directly supports output from Centrifuge [7], LMAT [21], CLARK [39], CLARK-S [40], and Kraken [41]. Alternative taxonomic classifiers are supported through a generic interface developed to handle different file formats with comma-separated values (CSV), tab-separated values (TSV), or space-separated values (SSV). The software also includes support for LMAT plasmid assignment system [15]. For implementation details of the Recentrifuge computing kernel please see S1 Appendix, S5 and S6 Figs.

**Fig 5:**
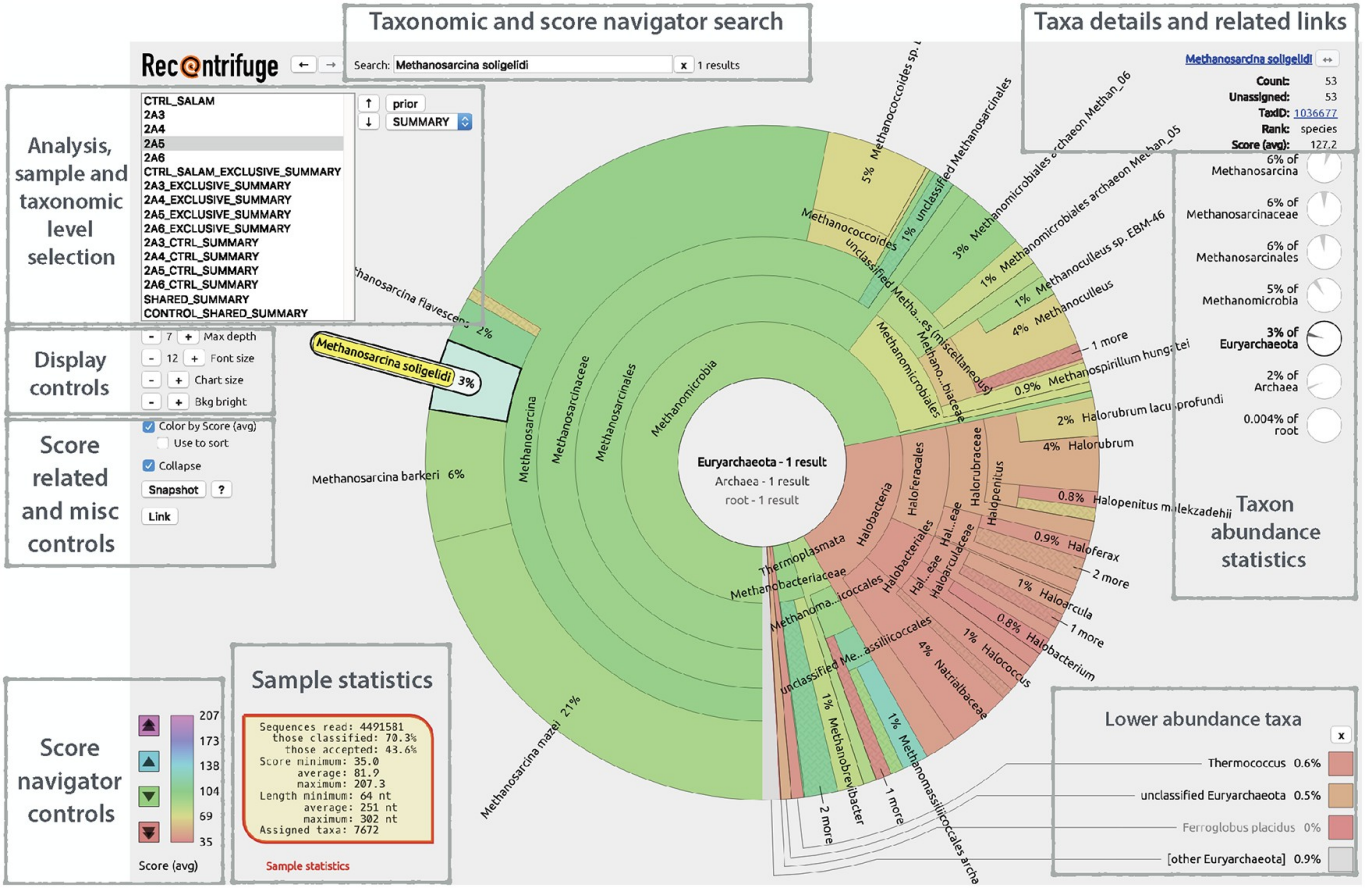
Layout of the Recentrifuge interface. This figure is an explained screenshot of the Recentrifuge web interface for an SMS study (see S1 Fig for details of the example). It highlights the principal parts of the interface, which are also labeled. The sample 2A5 was selected (see the sample selection box in the top left under the Recentrifuge logo), so the key statistics for this sample appeared in the bottom left of the view. In the center, there was the corresponding hierarchical pie chart, with zoom in the phylum Euryarchaeota. For each taxon, the background color reflected the average confidence level of the taxonomic classification following the scale plotted in the bottom left of the figure, where there were also buttons for the score navigator. Since the interface had the option disabled, Recentrifuge did not sort the taxa according to the average confidence level. In this particular case, the taxon *Methanosarcina soligelidi* was selected in the pie chart, thus prompting the display of taxon-related statistics and links in the top right of the figure. The current links were to Google Scholar and NCBI Taxonomic Browser. The statistics included: the number of reads assigned to this or lower taxonomic levels (Count) and their average confidence (Score —avg—), the number of reads just assigned to this level (Unassigned), the NCBI taxid (TaxID) and rank (Rank), and some information about relative frequencies.

To ensure the broadest portability for the interactive visualization of the results, the central outcome of Recentrifuge is a stand-alone HTML file which can be loaded by any JavaScript-enabled browser. Fig 5 shows a labeled screenshot of the corresponding Recentrifuge web interface for an example of SMS study (see S1 Fig). A vectorial screenshot in SVG format with the original font scheme is available for any sample using the “Screenshot” button of the user interface. The package also provides comprehensive statistics about the reads and their classification performance. Another Recentrifuge output is a spreadsheet collection detailing all the classification results in a compact way. This format is adequate for further data mining on the data, for example, as input for applications such as longitudinal (time or space) series analyzers like *Dynomics* (in development). Besides, the user can choose between different score visualization algorithms, some of which are more interesting for datasets containing variable length reads, for example, the ones generated by Oxford Nanopore sequencers.

Finally, some filters are available, like the minimum score threshold (minscore), which can be set independently for the control and real samples. The minscore filter can be used to generate different output sets from a single run of the classifier with a low minimum hit length (MHL) setting, saving computational resources. Other filters are mintaxa, described in the scored taxonomic trees subsection, and the lists of identifiers to exclude or include a taxon and all its children in the taxonomic tree.

The additional tools in the Recentrifuge package (see Fig 4) can generate further products and results. *Rextract* is a script which helps in extracting a subset of classified reads of interest from the single or paired-ends FASTQ input files. This set of reads can be used in any downstream application, such as genome visualization and assembling. *Remock* is a script for easily creating mock Centrifuge samples, which is useful not only for testing and validation purposes but also for introducing a list of previously known contaminants to be taken into account by the robust contamination removal algorithm. *Retest* is the code used for continuous integration (CI) testing and algorithm verification procedures (see Section 2 of S4 Appendix for further details and S10 Fig for its flowchart).

## Results and discussion

### Recentrifuge accurately removes cross-contamination

We developed a synthetic dataset carefully designed to challenge the Recentrifuge algorithms (see S13 Fig and Section 2.3 of S4 Appendix for details), thus enabling a quantitative assessment of the capability of the method to cope with different kinds of contaminants. We also devised this mock dataset in order to evaluate the ability of the method to deal with cross-contamination between samples. This feature of Recentrifuge is one of the advantages of this novel approach. In addition, this synthetic dataset serves the purpose of the continuous integration framework of the software, as the results of processing these data are compared with a standard to check the reliability of the method after any change in the source code.

**Fig 6:**
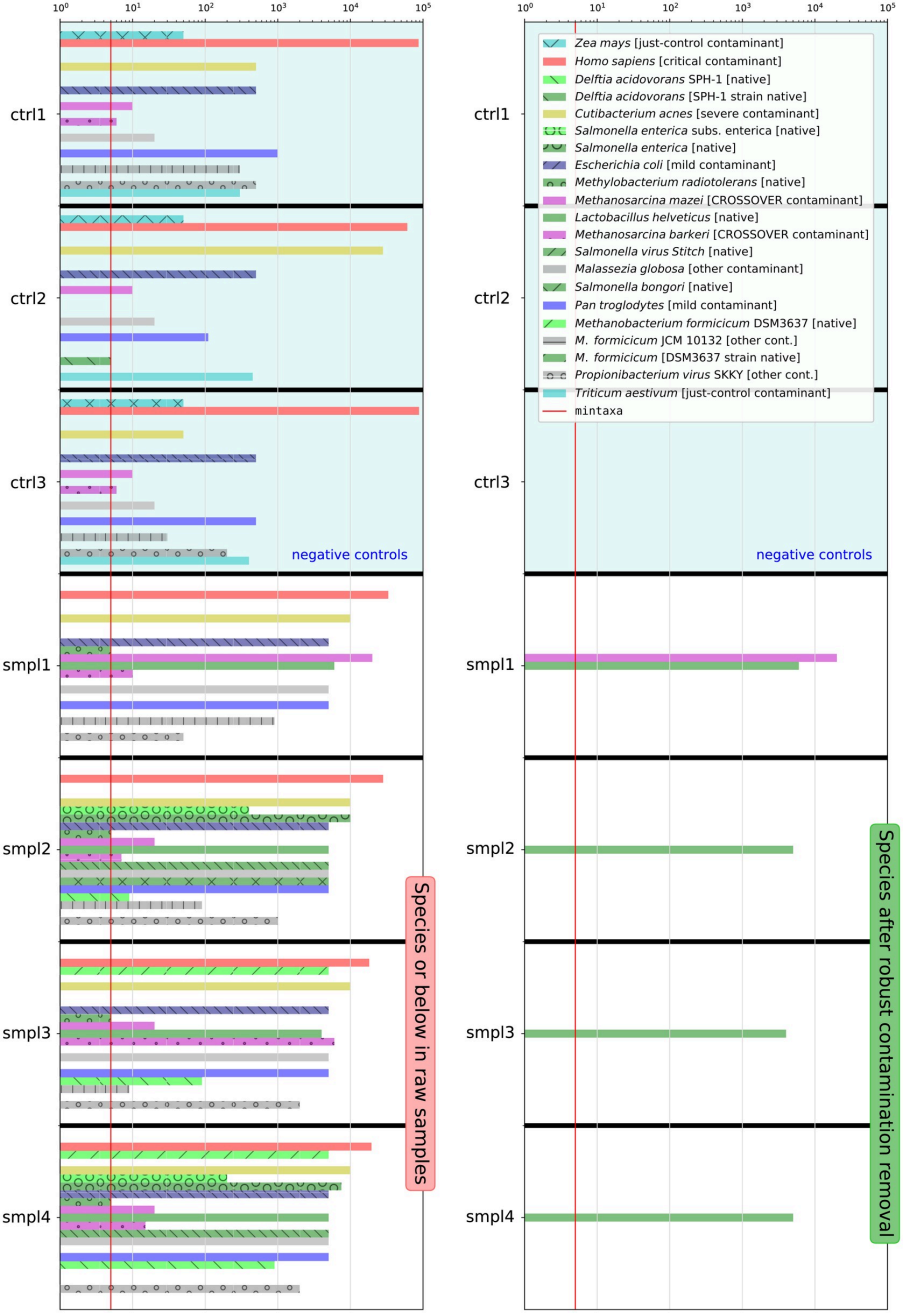
Comparison of abundance histograms for some taxa (species or below) in the synthetic dataset before (raw samples) and after the robust contamination removal (CTRL_species samples). Data shown for samples smpl1 to smpl4 and the negative control samples (ctrl1 to ctrl3), which were used by the contamination clearing process without modification. The legend of S13 Fig details the color code of the taxa. Here, the legend contains the name of each taxon followed by a note given in brackets; this remark is indicating either the type of contaminant, or which is the native strain of a species, or the native source for cross-contamination. Finally, the mintaxa level is drawn as a red line crossing all the samples.

Fig 6 shows a comparison of abundances of taxa included in the synthetic dataset before and after the Recentrifuge robust contamination removal algorithm. The taxa belong to species or below in the NCBI taxonomy. The left column of the figure shows the abundance histogram for seven raw samples: four real samples (smpl1 to smpl4) plus three negative control samples (ctrl1 to ctrl3). Similarly, the right column shows the results after the algorithm intervention for the species taxonomic level, i.e., the corresponding CTRL_species samples (see ‘Derived samples’ subsection in Design and implementation). Native taxa are green-colored, crossover contaminants are colored in purple, and other colors indicate different classes of contaminants. The legend of S13 Fig details the complete color code.

We see in Fig 6 that Recentrifuge cleared the CTRL_species samples of the different contaminants (species and below) found in the negative control samples while retaining the particular native taxa, which accumulated up to the species level (see ‘Scored taxonomic trees’ subsection in Design and implementation for details). Examples of important contaminants removed were human reads and those belonging to *Cutibacterium acnes*. The algorithm also deleted more subtle contamination, such as the reads assigned to *Malassezia globosa*. Crossover contamination requires special mention. On the one hand, *Methanosarcina mazei* was ubiquitous among the samples, but it was only native to smpl1 and a contaminant in the rest. On the other hand, *M. barkeri* was present in the four real samples despite being only native to smpl3, but it was scarce in the control samples, even missing from ctrl2. Recentrifuge accurately detected which were the source sample of both *Methanosarcina* species, thus keeping the native reads there and clearing the cross-contamination from the rest of the samples.

Furthermore, we included an additional sample (smplH) in the synthetic dataset containing the 241 species of a high-complexity dataset used as a gold standard for benchmarking metagenomic software [47]. As with the other samples, this specimen combined contaminants as additional taxa. In addition, we spiked the controls with low abundances of native taxa from this and the other real samples in order to simulate statistical noise in negative control samples such as low-frequency misclassifications and sequencing errors. We used the complete synthetic dataset to obtain different ROC (receiver operating characteristic) plots. S11 Fig shows the evolution of the sensitivity and specificity from the raw specimens to the CTRL_species samples. Basically, this ROC presented a transition from a scenario of very low specificity, on account of the contamination misidentified as native taxa, to a situation characterized by very high specificity, thanks to the correct detection of contaminants, including crossovers. For some samples, this came at the expense of a slight loss in the sensitivity. The reason for that small decline in the recall rate was the intentional introduction in the synthetic dataset of the archaea *Methanobacterium formicicum* with two different strains, one native to the samples (*M. formicicum* DSM 3637) and another a contaminant (*M. formicicum* JCM 10132). At the species level, once the cross-contamination situation was ruled out, Recentrifuge followed a conservative strategy and deemed the archaeal species as a contaminant and, therefore, the native strain of *M. formicicum* became a false negative thus decreasing the sensitivity. For samples smpl1 to smpl4 and smplH, S12 Fig shows the ROC as a function of the mintaxa parameter. Results of Fig 6, S11 and S12 Figs can be easily replicated using *retest* (see Section 2 of S4 Appendix).

### New biological insight into a highly contaminated plasma study

To confirm Recentrifuge’s ability to analyze complex metagenomes and provide new biological insight, we considered an ambitious but severely contaminated SMS study of RNA in plasma from individuals with Myalgic Encephalomyelitis/Chronic Fatigue Syndrome (ME/CFS), alternatively diagnosed chronic Lyme syndrome (ADCLS), and systemic Lupus erythematosus (SLE) [48]. This research suffered from large batch and contamination effects and was unable to find a positive association between the plasma microbial content of sick individuals, thus highlighting the relevance of technical controls in metagenomics. More than 240 giga-base-pairs of raw genomic data distributed in 67 samples with paired-ends sequences were downloaded and analyzed using Bowtie2 [49], Centrifuge [7], and SAMtools [50] (see S2 Appendix for the procedure details). Recentrifuge analyzed the different datasets in this study using stricter parameters than the default ones: sequencing of RNA required extra steps than sequencing of DNA, including the reverse transcription of RNA and further purifications [48], which were additional sources of artifacts and contamination. In this case, an increase in the matching length to 60 was advisable [7], so Recentrifuge filtered the Centrifuge output with minscore raised to an even stricter value of 75 (unless otherwise indicated).

To further illustrate the difficulty of the dataset of the SMS study of plasma in ME/CFS patients regarding the contamination, just a couple of results. First, affecting the sequencing batch one, Recentrifuge detected crossover contamination in the negative control samples with the source in the positive control, consisting of *human metapneumovirus* (hMPV). Second, Recentrifuge reported quite more different taxa in the negative controls than in the normal samples: 65% and 22% more on average, respectively, for the batch two and three. The presence of generalized crossover contamination complicates the removal of the contaminants in the samples by merely excluding the taxa present in the controls. Here it is when the robust contamination removal algorithm of Recentrifuge is of great help: it detects the crossover contaminants (hMPV and other taxa) and removes them from all the samples except for the inferred source. Therefore, the positive control is still positive for hMPV after the contamination removal, as expected (see S7 Fig).

The Recentrifuge analysis of the entire collection of 67 samples revealed the presence of ubiquitous contaminants able to spread over different sequencing batches and type of samples (see S8 Fig). Most of the contaminating bacteria are known contaminants belonging to the *kitome* [11]. Other pervasive pollutants belong to the fungi orders Eurotiales, Helotiales, Hypocreales, Pleosporales, and Saccharomycetales. The contamination by Apicomplexa, in general, and *Plasmodium vivax* and *Besnoitia*, in particular, can be linked to database contamination [15, 20] and seems a negative hallmark of SMS RNA studies related to body fluids [8]. An interesting complementary analysis consisted of retrieving those taxa that are contaminating the negative control samples exclusively. S9 Fig shows the genera contaminating all the control samples but no other specimen along the second batch, representing contaminants which entered the workflow in some procedure or material exclusive to the control samples.

**Fig 7:**
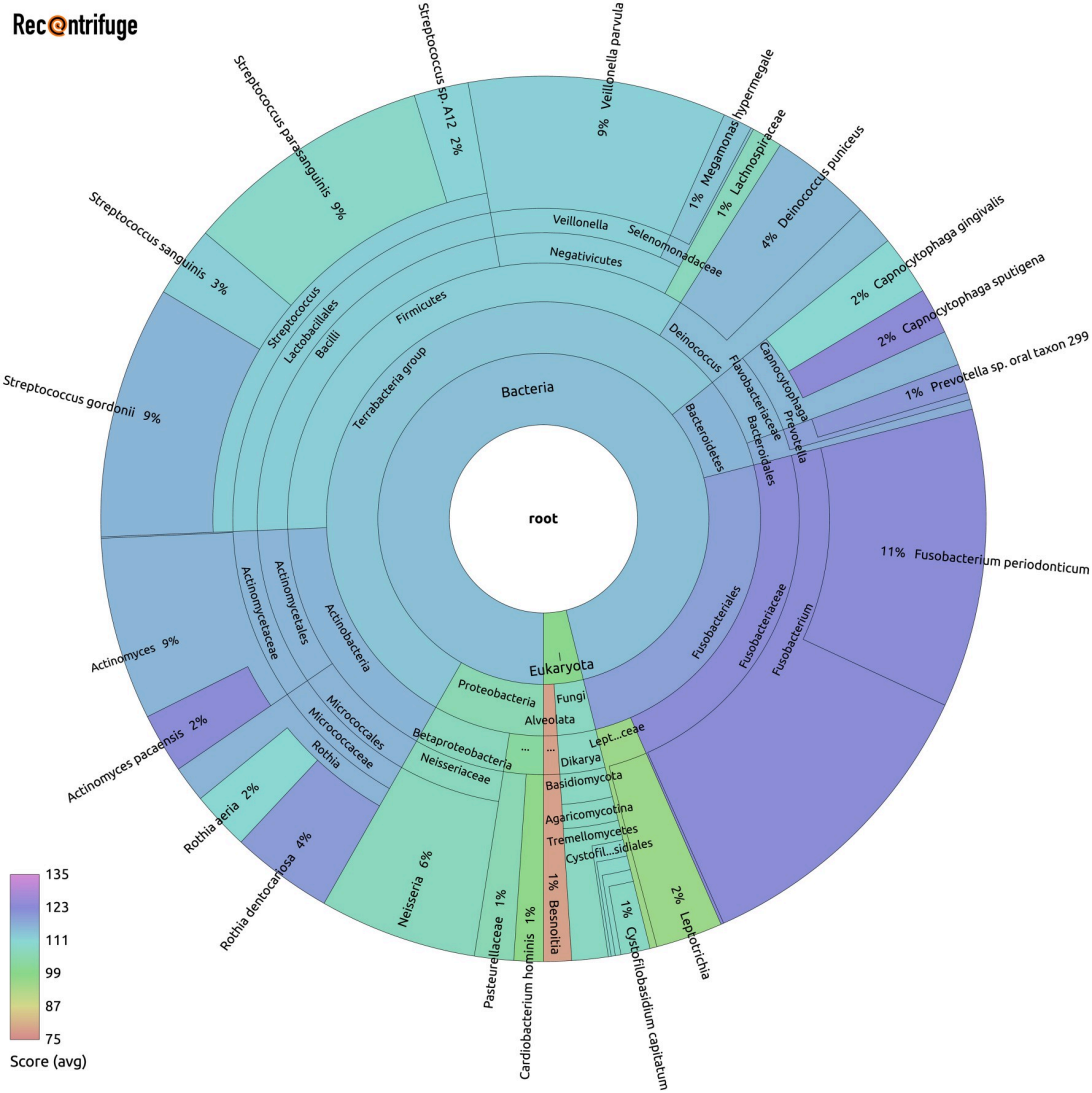
Taxa after contamination removal in a sample of the SMS study of plasma in ME/CFS patients. This is for an ADCLS patient (sample 56), showing a high average score (114) in the classification. The microbial distribution seems compatible with bacteria translocated from the oral cavity into blood, probably because of a chronic inflammatory polymicrobial disease.

In concordance with the main conclusion of the study of plasma in individuals with ME/CFS [48], Recentrifuge did not find shared taxa after control removal (CONTROL_SHARED empty) when analyzing the samples rearranged in different batches and pathology/healthy groups. Nevertheless, the individual analysis of the samples after contamination removal presents interesting features in a case-per-case review. That is the case of sample 56 in Fig 7, which belongs to an ADCLS patient. It shows a collection of taxa with a high average score (114) in the classification, implying that a majority of sequences mapped in both reads from the pair, except for the contaminant genus *Besnoitia*, the lowest-scored one. This set of microbes seems compatible with bacteria translocated from the buccopharyngeal cavity into blood, apparently because of an oral chronic inflammatory polymicrobial disease. However, the clinically relevant taxa in this study go far beyond those of sample 56 shown in Fig 7. S3 Appendix portrays other representative bacteria, viruses, and fungi, present in the samples.

Research in recent years is overturning the commonly accepted paradigm which stated that, in healthy individuals, the tissues and body fluids not in contact with the environment are sterile. Healthy organs once thought to be free of microbes are crawling with bacteria, archaea, viruses, and eukaryotes. The shift of paradigm has spread to more and more tissues and fluids, like the deepest layers of the skin [51], the placenta [52], the urine [53], the blood [54, 55], the breast milk, or others [54, 56, 57].

The plasma is the part of the blood with the lower proportion of bacterial DNA, only 0.03% [55]. In the reanalyzed study of plasma in individuals with ME/CFS [48], the intrinsic difficulties of ultra-low microbial biomass joined the handicap of an RNA sequencing technique prone to further artifacts and biases, which resulted in severe widespread contamination. With the results of the research, the classical paradigm might seem supported, that is, the idea of the absence of a plasma microbiota in healthy individuals. However, the authors of the study believed that the limitation of the current techniques prevented them from revealing the microbial component in human plasma. Indeed, with the *noise* in the same order of magnitude of the *signal*, a robust method for contamination removal was required to tackle this complex dataset. Despite all the difficulties, the analysis with Recentrifuge has unveiled a meaningful plasma microbiota in the samples (Fig 7 and S3 Appendix). The results are in line with the recent research in the field, which points out the gut, the oral cavity, and the genitourinary tract as the primary sources of the blood microbiome [55, 58, 59].

In conclusion, thanks to the robust contamination removal and the score-oriented comparative analysis of multiple samples in metagenomics, Recentrifuge can play a key role, firstly, in the study of oligotrophic microbes in environmental samples, as it did by showing that microbiomes of Arctic and Antartic solar panels display similar taxonomic profiles [60]; secondly, in the more reliable detection of minority organisms in clinical or forensic samples. The relevant organisms found with a high score in the SMS study of plasma in ME/CFS patients [48] after the robust contamination removal are good examples. Finally, the mock dataset confirmed the worthiness of the developed methods, which demonstrated a radical improvement in specificity while retaining high sensitivity rates even in the presence of cross-contaminants.

## Availability and future directions

Recentrifuge’s main website is www.recentrifuge.org. The data and source code are anonymously and freely available on GitHub at https://github.com/khyox/recentrifuge and PyPI at https://pypi.org/project/recentrifuge. The Recentrifuge computing code is licensed under the GNU Affero General Public License Version 3 (www.gnu.org/licenses/agpl.html). Recentrifuge’s continuous integration (CI) information is public on Travis CI at https://travis-ci.org/khyox/recentrifuge.

The wiki (https://github.com/khyox/recentrifuge/wiki) is the most extensive and updated source of documentation for Recentrifuge, including installation, testing, quick-start, and comprehensive use cases for the different taxonomic classification engines supported. In addition, Recentrifuge’s installation is explained in Section 1 of S4 Appendix, testing is detailed in Section 2 of S4 Appendix, and running Recentrifuge for Centrifuge, LMAT, CLARK flavors, Kraken, and other taxonomic classifiers are subsections of Section 3 of S4 Appendix. Similarly, Sections 4 and 5 of S4 Appendix describe running Rextract and the Recentrifuge command line, respectively. Finally, Section 6 of S4 Appendix includes troubleshooting subsections.

The full Centrifuge output and the detailed Recentrifuge results for the SMS study of plasma in individuals with ME/CFS are publicly available at som1.uv.es/plasmaCFS.

Just as the biochemical profile and cell count are currently usual blood tests, metagenomic analysis of the blood will probably become a standard in a few years. The methods that will pave the way for a well-established clinical practice of metagenomics are still to come. As an open-source project, the participation of the computational biology and clinical metagenomics community will determine the future of Recentrifuge considerably.

An important extension to Recentrifuge is under active development and will be released soon. It is “Regentrifuge”, the counterpart of Recentrifuge in the area of metagenomic functional analysis.

## Supporting information

S1 Fig Example of longitudinal SMS study. In longitudinal metagenomics, scientists retrieve and analyze sets of sequences belonging to microbial communities from different sources, times, patients, or body sites to unravel spatial, temporal or clinical patterns in the microbiota. This figure is an example outlining the problem of comparing different but related samples in a longitudinal SMS study. The sample named 2A is subdivided longitudinally into six subsamples whose DNA/RNA is extracted along with negative control samples. The purified DNA/RNA is then sequenced, and the generated sequencing reads are processed through a metagenomics analysis pipeline, such as the one detailed in S2 Fig. A collection of different datasets are finally produced, which should be adequately compared to elucidate lengthwise patterns in the microbiota within the 2A sample.
(PDF)

S2 Fig Typical steps of an SMS study. A longitudinal study involving SMS, like the one illustrated by S1 Fig, spans in some stages to obtain valuable field-domain information starting from the original samples. For each specimen, the researcher extracts DNA/RNA using a commercial kit, a custom protocol optimized for the type of sample, or a combination of both. Next, a technician prepares a library matching the target sequencing technology with the purified DNA/RNA, which is then sequenced. A bioinformatics pipeline processes the reads that the sequencer provides. We could roughly separate such process in three consecutive steps. First, in the pre-analysis, codes like FastQC (Babraham Bioinformatics, 2016) and MultiQC [61] quality-check the reads. Second, in the analysis stage, the most computationally intensive one, software packages like LMAT [21], Kraken [41], CLARK [39], Centrifuge [7], and CLARK-S [40] (see S3 Fig for details) classify the reads taxonomically or functionally. Finally, in the post-analysis step, different tools like Krona [42], Pavian [62], or Recentrifuge further process the results to enable more in-depth analysis and improved visualization.
(PDF)

S3 Fig Computational analysis in an SMS study. The core phase of a metagenomics analysis pipeline (see S1 and S2 Figs for the outline of the bioinformatic phases) is carried out by high performance computing software. These are intensive codes in both CPU and memory (sometimes, they are input/output intensive too), such as LMAT [21], Kraken [41] and, more recently, CLARK-S [40] and Centrifuge [7]. All these tools are performing taxonomic classification and abundance estimation, whereas LMAT is also able to annotate genes. For the taxonomic classification, both LMAT and Kraken use an exact k-mer matching algorithm with large databases (~100 GiB) while Centrifuge use compression algorithms to reduce the databases size (~10 GiB) but at some speed expense. CLARK-S use discriminative spaced k-mers to improve the sensitivity but with a toll on the performance. The most complete LMAT database is approaching half terabyte of required memory while the Centrifuge database generated in-house in March 2018 from the NCBI Nucleotide [63]*nt* database (~170 GiB) occupied just 105 GiB. The equivalent spaced k-mers database of CLARK-S generated in May 2018 took 267 GiB of disk space.
(PDF)

S4 Fig Summary of advantages of Recentrifuge. This figure summarizes the immediate benefits of applying Recentrifuge to a study involving SMS of different but related samples, including negative controls (see S1 Fig). Recentrifuge generates four different sets of scored charts for each taxonomic level of interest in addition to the scored plots for the raw samples: samples with the control taxa subtracted, the exclusive taxa per sample and the shared taxa with and without control taxa subtracted. This battery of analysis and plots permits robust comparative analysis of multiple samples in low microbial biomass metagenomic studies.
(PDF)

S5 Fig Hierarchy of the NCBI Taxonomy. All the 32 ranks are supported by Recentrifuge. This illustration is based on the 9-rank hierarchy publicly released by Peter Halasz.
(PDF)

S6 Fig UML class diagram of Recentrifuge. This graph summarizes the relationships between developed classes in the Recentrifuge core package. The classes in the figure with a colored border are the parent classes from which the Recentrifuge ones derive. Those belong to the Python Standard Library (red border) and BioPython (green border).
(PDF)

S7 Fig Positive control with human metapneumovirus (hMPV). Screenshots of the Recentrifuge web interface showing results for samples with paired-ends sequences for the batch 1 of the SMS study of plasma in ME/CFS patients [48]. The 1^st^ batch samples with paired-ends sequences are two negative controls (samples S018 and S036) and the positive control (sample S008) with hMPV. In the figure, the top chart plots the control sample S018 (Ctrl1_S018_B1_Neg), highlighting the hMPV contamination. The bottom chart shows the summary sample for the positive control after the robust contamination removal (S008_B1_MPV_CTRL_SUMMARY), which kept the hMPV reads although hMPV contaminates the negative control S018. The filtering parameters for Recentrifuge were selected for allowing only high-scored taxa, with minscore of 75.
(PDF)

S8 Fig Global shared taxa. This scored pie chart shows the taxa shared between the 67 samples with paired-ends sequences of the ME/CFS plasma study [48]: those are ubiquitous contaminants able to spread over different sequencing batches and type of samples. For this analysis, Recentrifuge ran with minscore set to 25 in order to provide a more comprehensive detection of contaminants.
(PNG)

S9 Fig Control samples exclusive taxa. This plot shows the taxa (contaminants) exclusive to the four negative control samples of the 2^nd^ sequencing batch of the ME/CFS plasma study, at genus level, compared with the 28 non-control samples of the batch. These genera contaminate all the control samples but no other sample in the batch, so they should have been introduced in some step exclusive to the negative control samples. Ordered by score, we found the following bacterial genera with both score over 60 and relative frequency over 1%: *Aureimonas, Caulobacter, Kytococcus, Lawsonella, Gramella, Dermacoccus, Duganella, Dickeya, Exiguobacterium, Altererythrobacter, Citrobacter, and Rhodobacter*. In the eukaryotic domain, the melanized meristematic fungus *Pseudotaeniolina globosa* stood out because of its high average score. Recentrifuge ran with minscore set to 25 in order to provide a more precise detection of the exclusive contaminants of the control samples.
(PNG)

S10 Fig *Retest* flowchart diagram. The dotted lines indicate procedures previously completed to prepare the standard needed for comparisons in some stages of the testing workflow. The dashed lines denote optional procedures that are detailed in Section 2.2.2 of S4 Appendix.
(PDF)

S11 Fig ROC generated by *retest* showing the impact of the robust contamination removal. The ROC (receiver operating characteristic) plot is using the test results of Recentrifuge and the information in the mock dataset to calculate the evolution of the sensitivity and specificity from the raw specimens to the CTRL_species samples.
(PDF)

S12 Fig ROC generated by *retest* in order to study the dependence on the mintaxa parameter. The ROC (receiver operating characteristic) plot is using the test results of many executions of Recentrifuge and the information in the mock dataset to follow the evolution of the sensitivity and specificity of the CTRL_species samples when forcing different mintaxa values, some of them indicated by the numbers at the base of the arrows.
(PDF)

S13 Fig Rationale and design of the mock community. The synthetic community contains diverse contaminant and native taxa, whose precise role is indicated by the characteristic background color shown in the legend. For example, green background characterizes native taxa, while purple background indicates crossover contaminants (those contaminating the samples except the source sample, where they are native). Such color code is also observed by the detailed output of the robust contamination removal algorithm. The taxa are mainly species or below, but there are also taxa belonging to other more general levels. Spread over different orders of magnitude, the abundances are fine-tuned to challenge Recentrifuge algorithms and easily detect any problem during the testing. In addition, the sample smplH includes the 241 species and proportions of a high-complexity dataset used as a gold standard for benchmarking metagenomic software [47]. In the spreadsheet, the black rectangles surround the areas simulating statistical noise in negative control samples such as low-frequency misclassifications and sequencing errors. The constants shown in the legend are contamination classification parameters of the robust contamination removal algorithm. *Retest* triggers the parsing of these worksheets by *remock* to create the mock dataset that *rcf* analyzes during its testing.
(PNG)

S1 Appendix Computing kernel implementation details. (PDF)

S2 Appendix Bioinformatics in the ME/CFS plasma study before Recentrifuge. (PDF)

S3 Appendix Other microbial taxa found with possible sources of translocation into the blood in the SMS study of plasma for ME/CFS patients [48]. This appendix contains the most probable sources of translocation into the blood of other microbial taxa found [64–68].
(PDF)

S4 Appendix Recentrifuge user manual. (PDF)
