# Correction: Recentrifuge: Robust comparative analysis and contamination removal for metagenomics

**DOI:** 10.1371/journal.pcbi.1007131

**Published:** 2019-06-17

**Authors:** Jose Manuel Martí

Fig 6 is missing information due to a bug in the software used to rotate the vectorial figure generated by Recentrifuge’s *retest* script. The author apologizes for missing this error. A corrected version is provided here.

**Fig 6:**
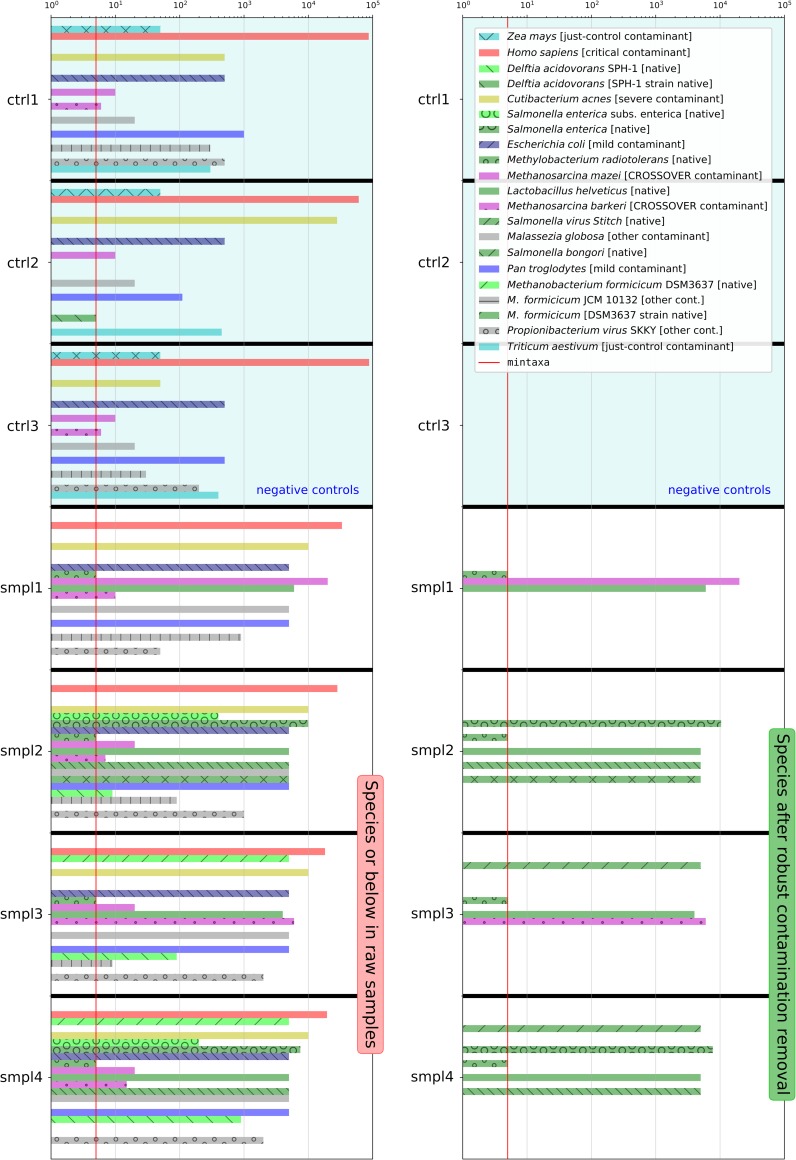
Comparison of abundance histograms for some taxa (species or below) in the synthetic dataset before (raw samples) and after the robust contamination removal (CTRL_species samples). Data shown for samples smpl1 to smpl4 and the negative control samples (ctrl1 to ctrl3), which were used by the contamination clearing process without modification. The legend of S13 Fig details the color code of the taxa. Here, the legend contains the name of each taxon followed by a note given in brackets; this remark is indicating either the type of contaminant, or which is the native strain of a species, or the native source for cross-contamination. Finally, the mintaxa level is drawn as a red line crossing all the samples.
