# Supplementary figures and images for: Recentrifuge: Robust comparative analysis and contamination removal for metagenomics
Source: PLoS Comput Biol. 2019 Apr 8;15(4):e1006967. doi: 10.1371/journal.pcbi.1006967 (PMC6472834; doi:10.1371/journal.pcbi.1006967)

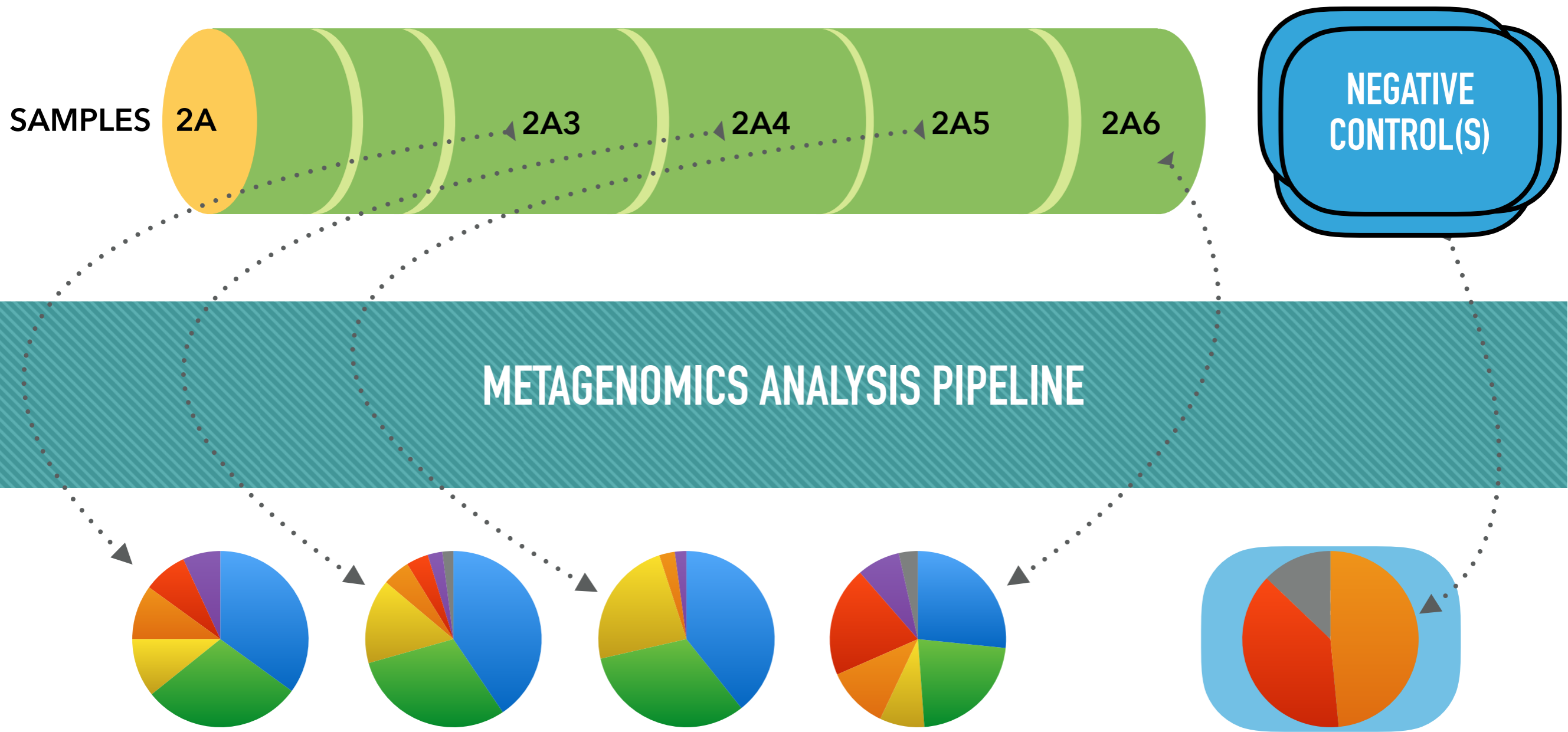

Supplement: S1 Fig — In longitudinal metagenomics, scientists retrieve and analyze sets of sequences belonging to microbial communities from different sources, times, patients, or body sites to unravel spatial, temporal or clinical patterns in the microbiota. This figure is an example outlining the problem of comparing different but related samples in a longitudinal SMS study. The sample named 2A is subdivided longitudinally into six subsamples whose DNA/RNA is extracted along with negative control samples. The purified DNA/RNA is then sequenced, and the generated sequencing reads are processed through a metagenomics analysis pipeline, such as the one detailed in S2 Fig. A collection of different datasets are finally produced, which should be adequately compared to elucidate lengthwise patterns in the microbiota within the 2A sample. (PDF) [file pcbi.1006967.s001.pdf]

# NCBI taxonomy

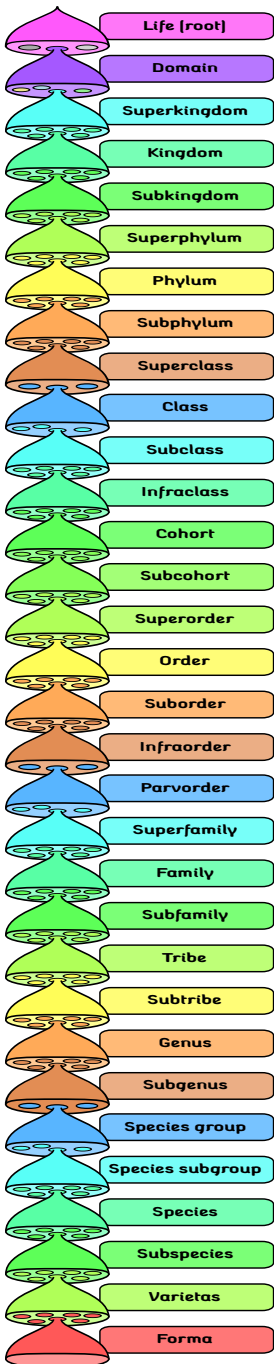

Supplement: S5 Fig — All the 32 ranks are supported by Recentrifuge. This illustration is based on the 9-rank hierarchy publicly released by Peter Halasz. (PDF) [file pcbi.1006967.s005.pdf]

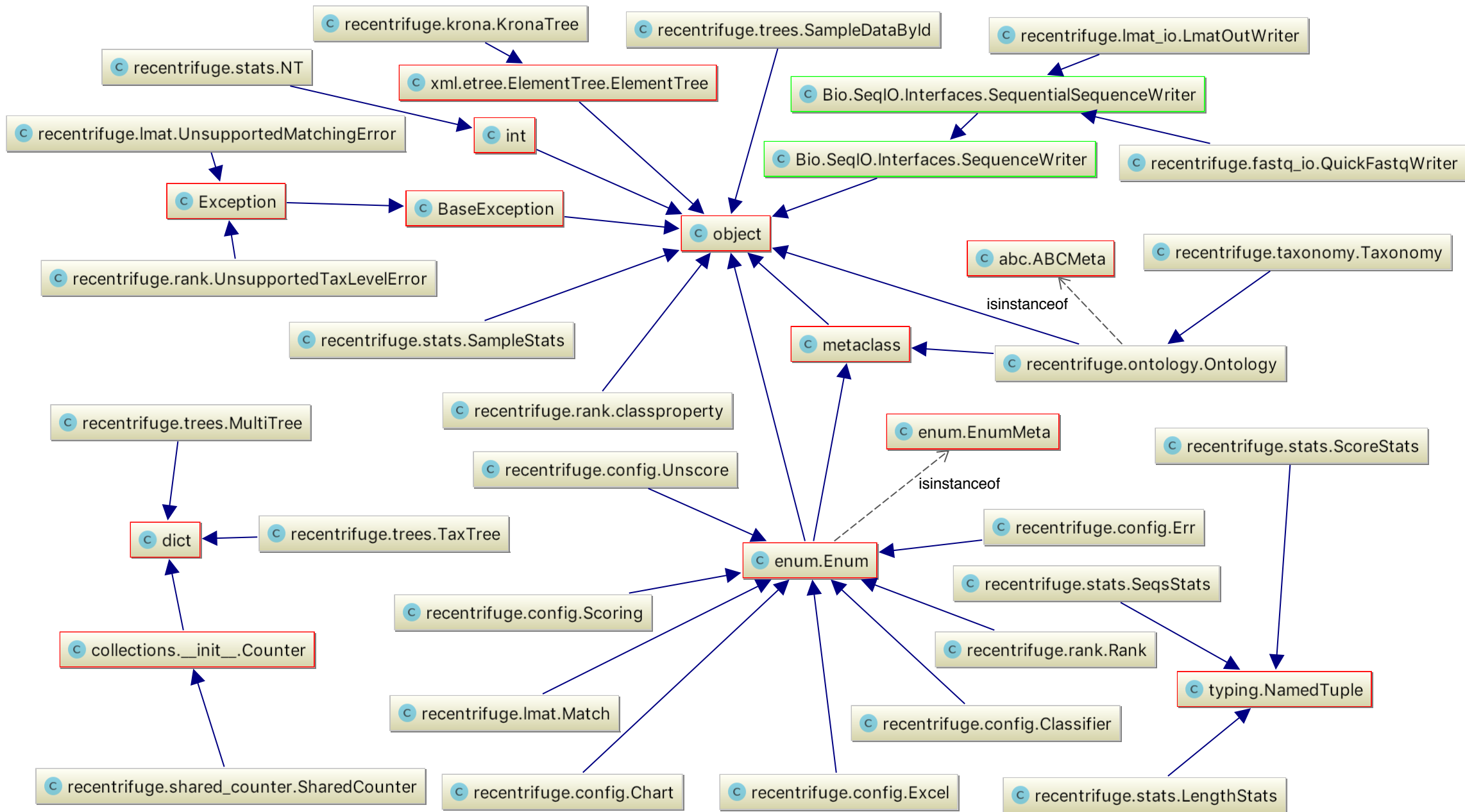

Supplement: S6 Fig — This graph summarizes the relationships between developed classes in the Recentrifuge core package. The classes in the figure with a colored border are the parent classes from which the Recentrifuge ones derive. Those belong to the Python Standard Library (red border) and BioPython (green border). (PDF) [file pcbi.1006967.s006.pdf]

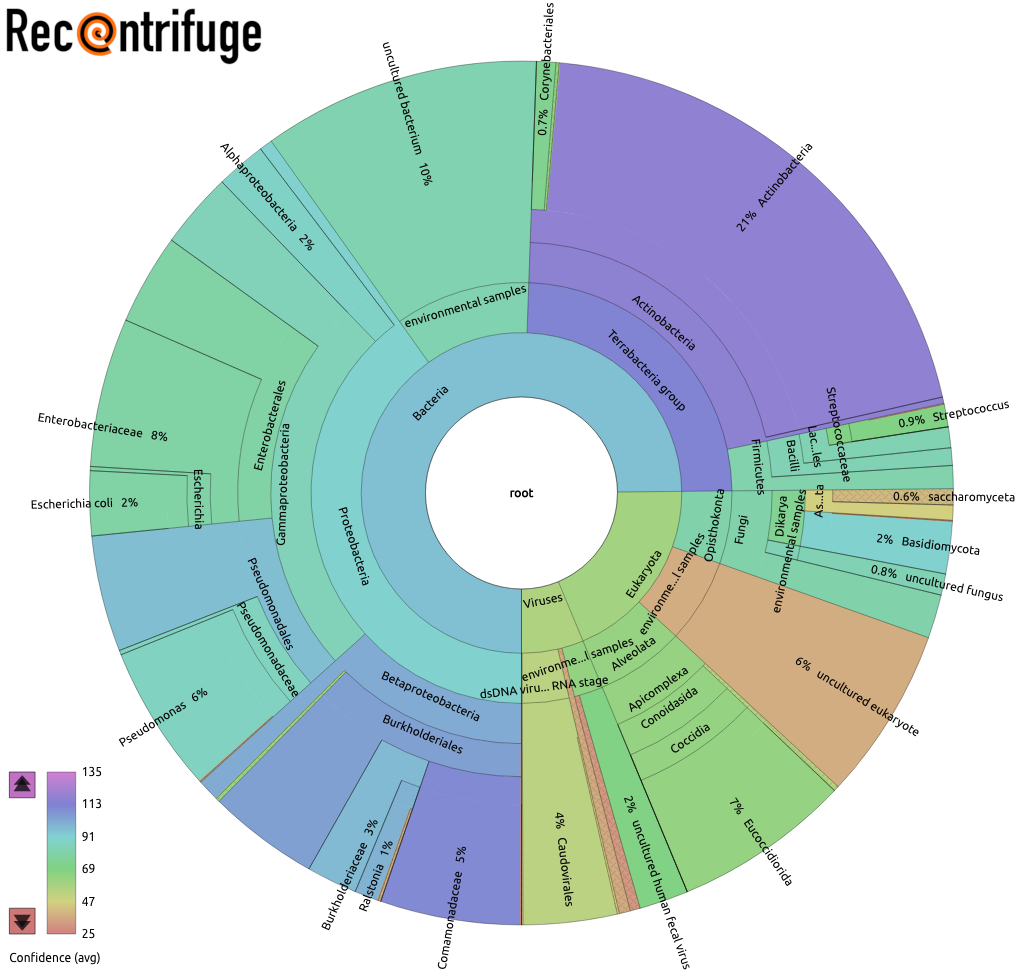

Supplement: S8 Fig — This scored pie chart shows the taxa shared between the 67 samples with paired-ends sequences of the ME/CFS plasma study [48]: those are ubiquitous contaminants able to spread over different sequencing batches and type of samples. For this analysis, Recentrifuge ran with minscore set to 25 in order to provide a more comprehensive detection of contaminants. (PNG) [file pcbi.1006967.s008.png]

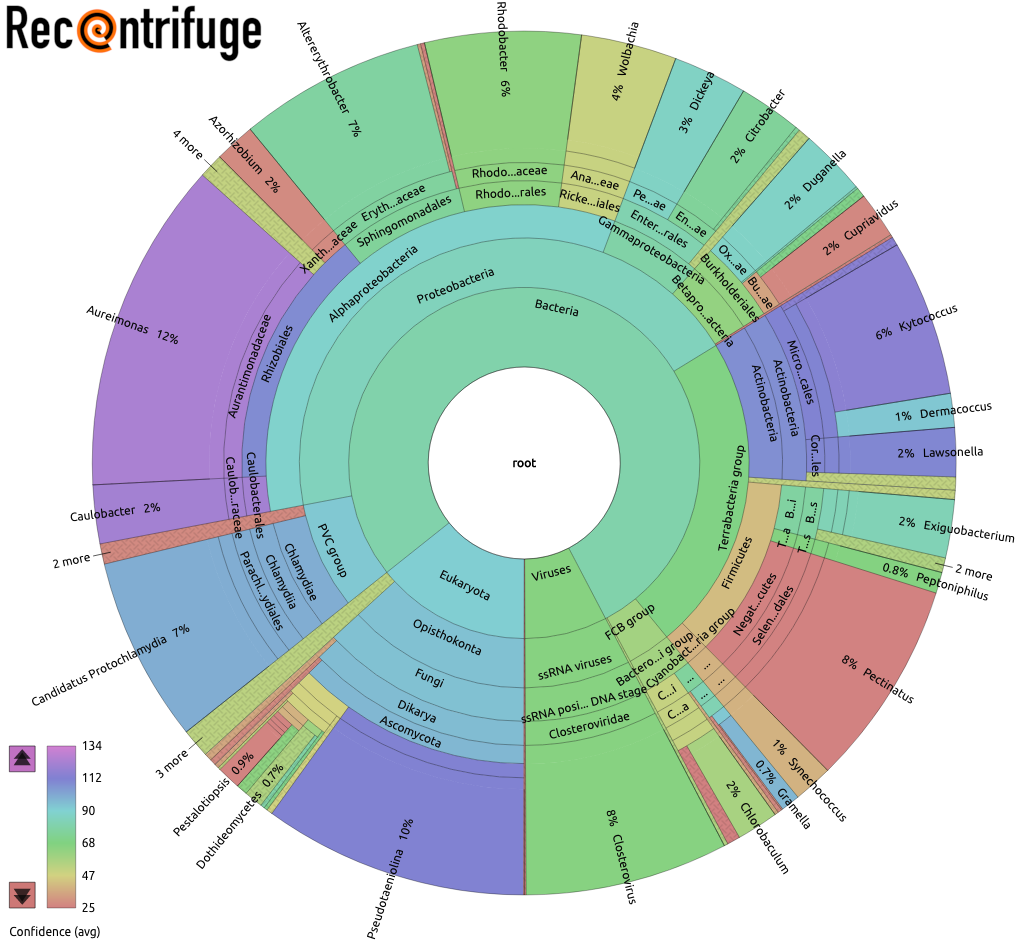

Supplement: S9 Fig — This plot shows the taxa (contaminants) exclusive to the four negative control samples of the 2nd sequencing batch of the ME/CFS plasma study, at genus level, compared with the 28 non-control samples of the batch. These genera contaminate all the control samples but no other sample in the batch, so they should have been introduced in some step exclusive to the negative control samples. Ordered by score, we found the following bacterial genera with both score over 60 and relative frequency over 1%: Aureimonas, Caulobacter, Kytococcus, Lawsonella, Gramella, Dermacoccus, Duganella, Dickeya, Exiguobacterium, Altererythrobacter, Citrobacter, and Rhodobacter. In the eukaryotic domain, the melanized meristematic fungus Pseudotaeniolina globosa stood out because of its high average score. Recentrifuge ran with minscore set to 25 in order to provide a more precise detection of the exclusive contaminants of the control samples. (PNG) [file pcbi.1006967.s009.png]

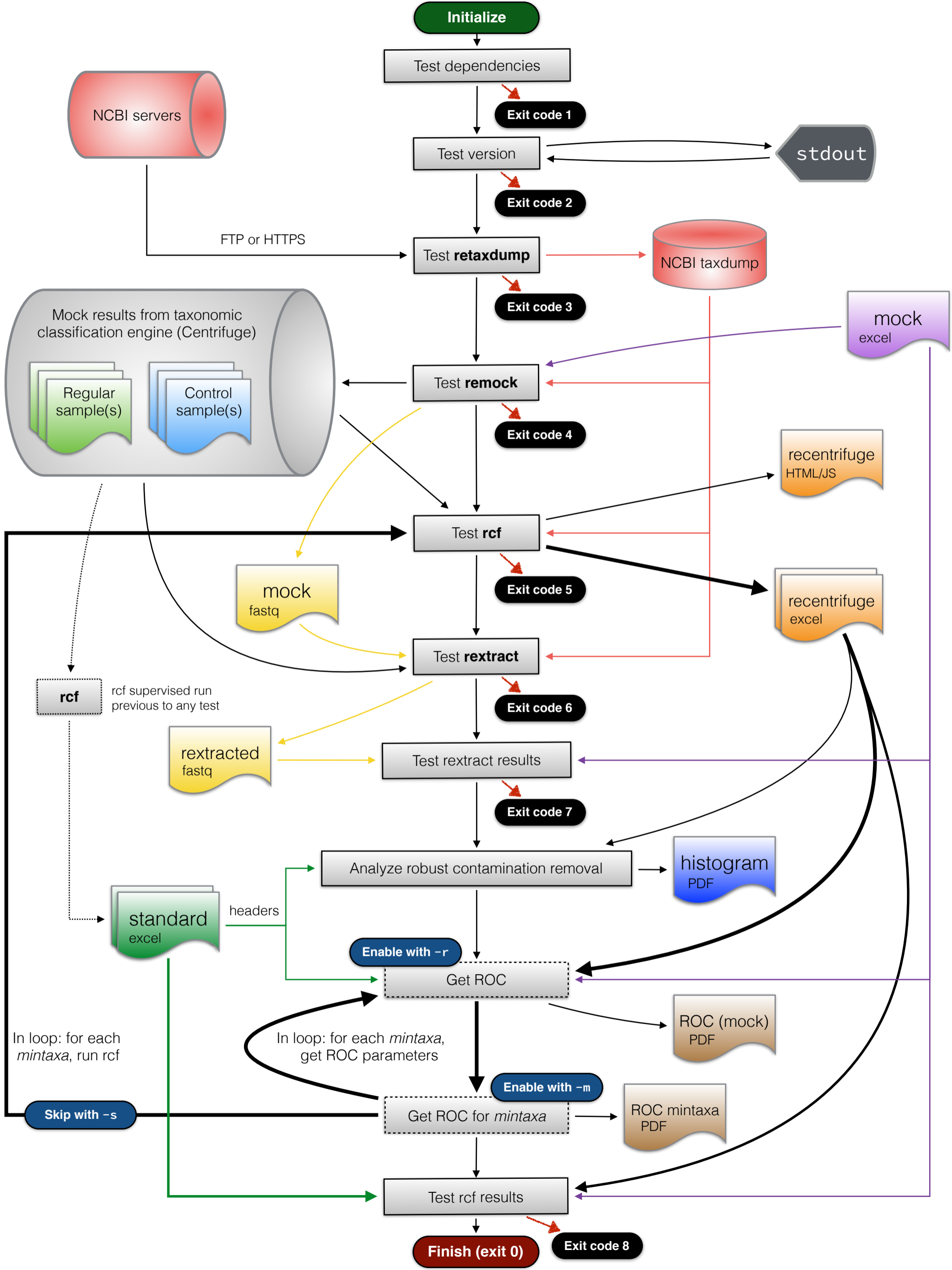

Supplement: S10 Fig — The dotted lines indicate procedures previously completed to prepare the standard needed for comparisons in some stages of the testing workflow. The dashed lines denote optional procedures that are detailed in Section 2.2.2 of S4 Appendix. (PDF) [file pcbi.1006967.s010.pdf]

Evolution from raw samples to CTRL\_species samples

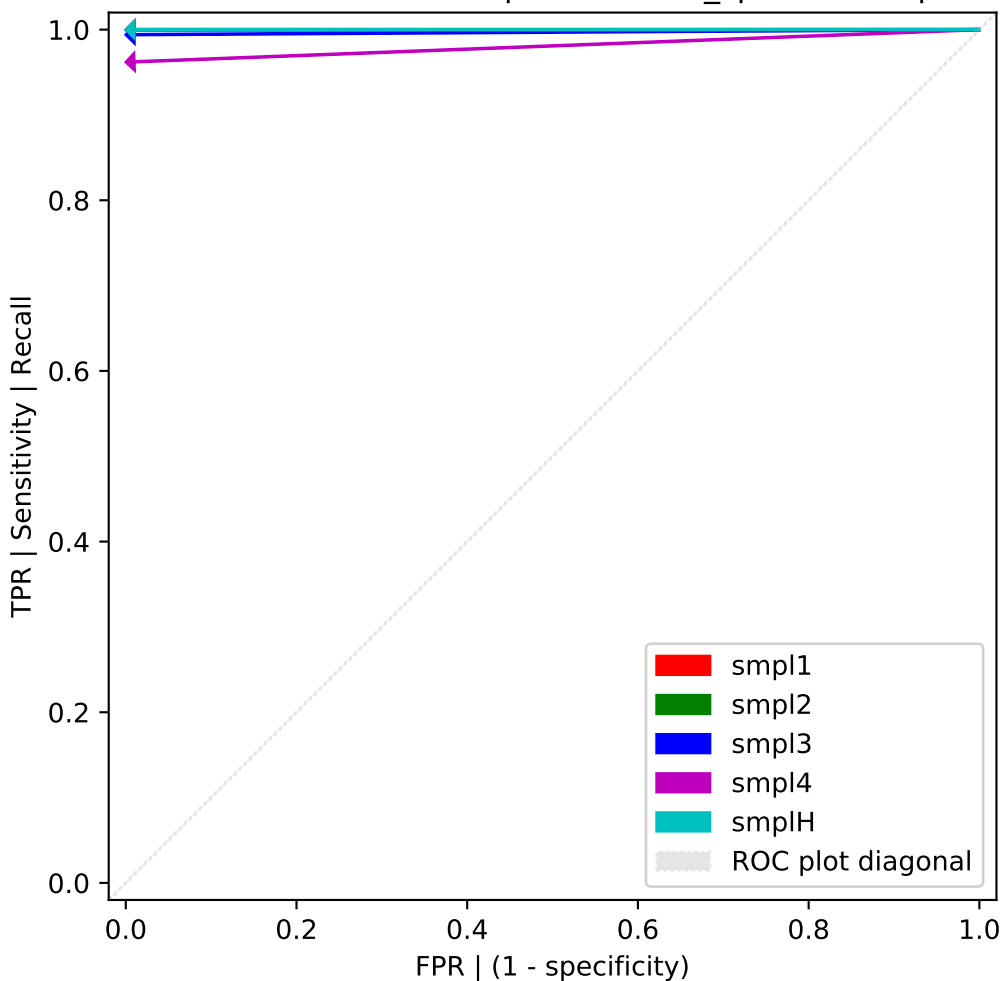

Supplement: S11 Fig — The ROC (receiver operating characteristic) plot is using the test results of Recentrifuge and the information in the mock dataset to calculate the evolution of the sensitivity and specificity from the raw specimens to the CTRL_species samples. (PDF) [file pcbi.1006967.s011.pdf]

# Evolution of ROC with mintaxa variation

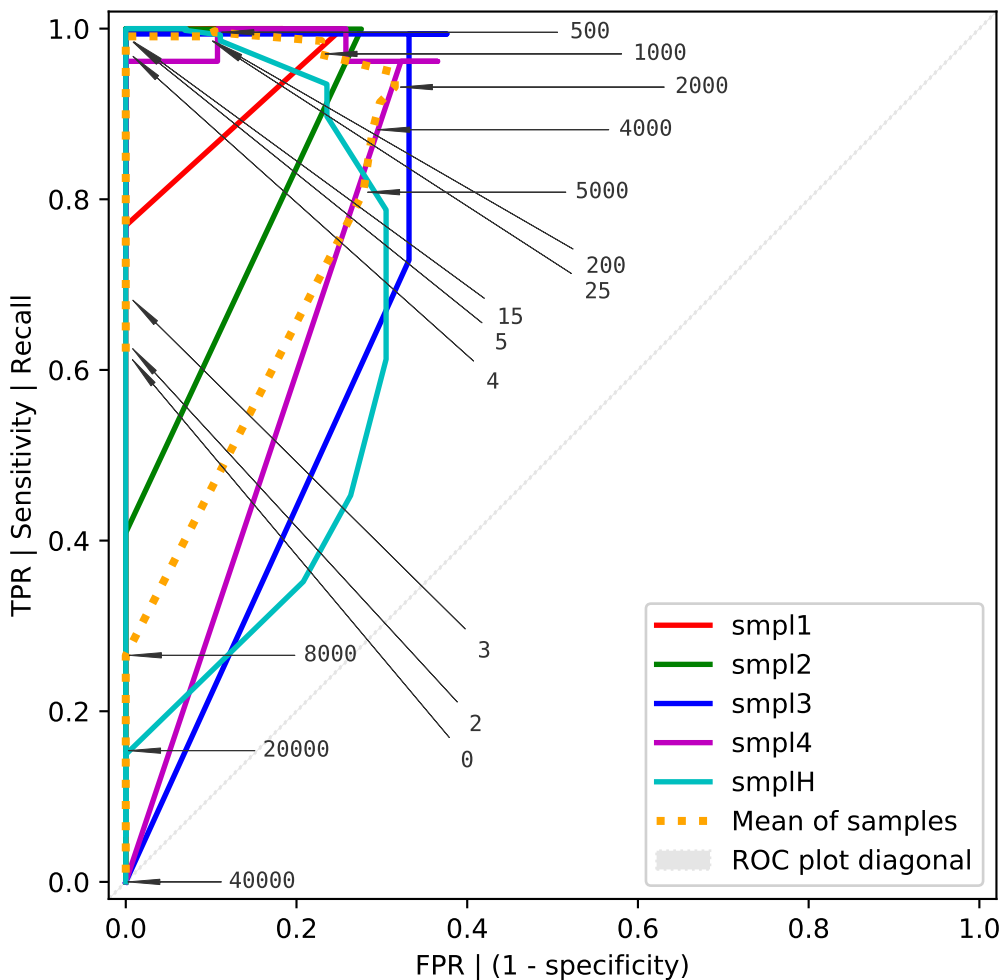

Supplement: S12 Fig — The ROC (receiver operating characteristic) plot is using the test results of many executions of Recentrifuge and the information in the mock dataset to follow the evolution of the sensitivity and specificity of the CTRL_species samples when forcing different mintaxa values, some of them indicated by the numbers at the base of the arrows. (PDF) [file pcbi.1006967.s012.pdf]

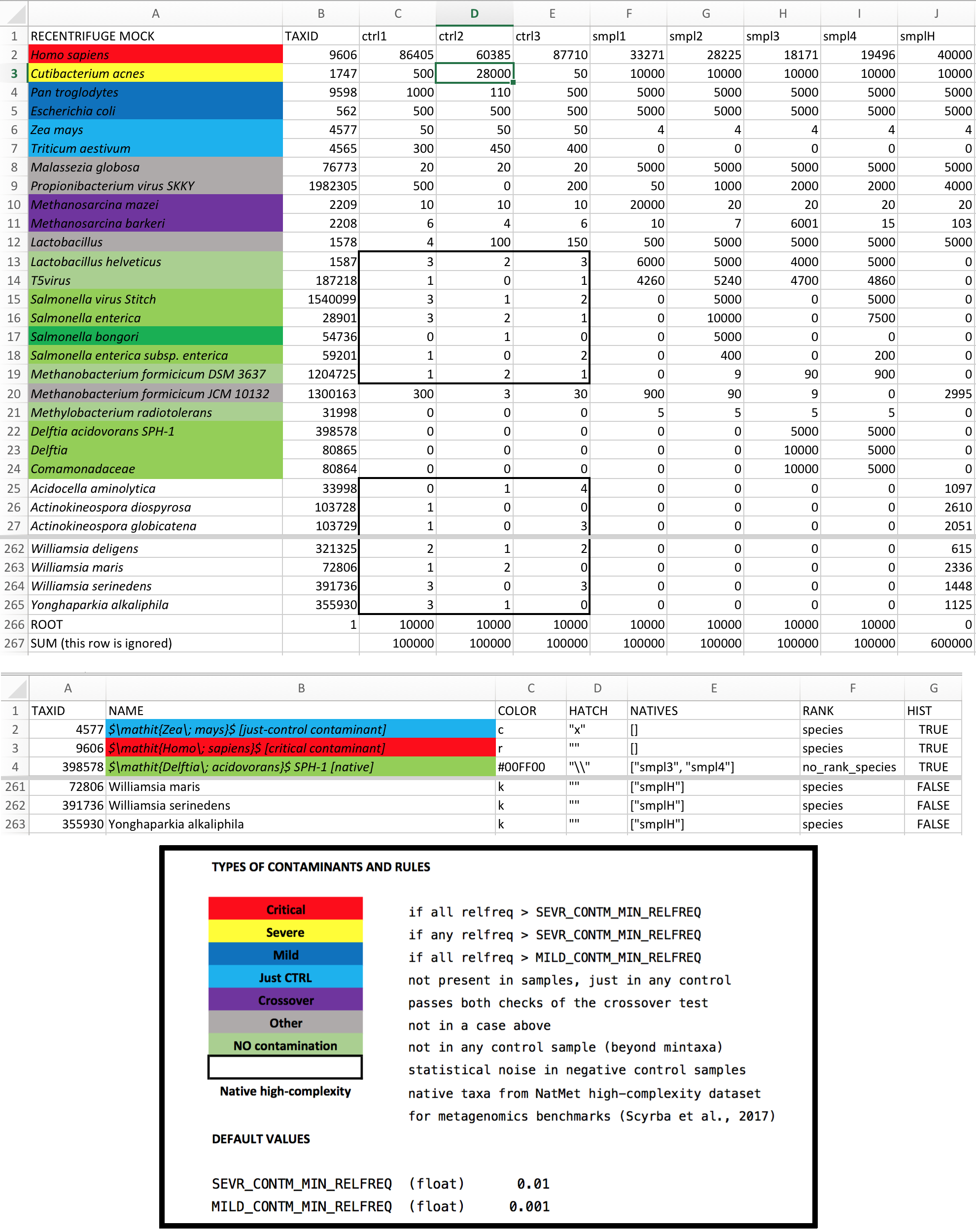

Supplement: S13 Fig — The synthetic community contains diverse contaminant and native taxa, whose precise role is indicated by the characteristic background color shown in the legend. For example, green background characterizes native taxa, while purple background indicates crossover contaminants (those contaminating the samples except the source sample, where they are native). Such color code is also observed by the detailed output of the robust contamination removal algorithm. The taxa are mainly species or below, but there are also taxa belonging to other more general levels. Spread over different orders of magnitude, the abundances are fine-tuned to challenge Recentrifuge algorithms and easily detect any problem during the testing. In addition, the sample smplH includes the 241 species and proportions of a high-complexity dataset used as a gold standard for benchmarking metagenomic software [47]. In the spreadsheet, the black rectangles surround the areas simulating statistical noise in negative control samples such as low-frequency misclassifications and sequencing errors. The constants shown in the legend are contamination classification parameters of the robust contamination removal algorithm. Retest triggers the parsing of these worksheets by remock to create the mock dataset that rcf analyzes during its testing. (PNG) [file pcbi.1006967.s013.png]
