# Supplementary material for: Recentrifuge: Robust comparative analysis and contamination removal for metagenomics
Source: PLoS Comput Biol. 2019 Apr 8;15(4):e1006967. doi: 10.1371/journal.pcbi.1006967 (PMC6472834; doi:10.1371/journal.pcbi.1006967)
Supplement: S2 Fig — A longitudinal study involving SMS, like the one illustrated by S1 Fig, spans in some stages to obtain valuable field-domain information starting from the original samples. For each specimen, the researcher extracts DNA/RNA using a commercial kit, a custom protocol optimized for the type of sample, or a combination of both. Next, a technician prepares a library matching the target sequencing technology with the purified DNA/RNA, which is then sequenced. A bioinformatics pipeline processes the reads that the sequencer provides. We could roughly separate such process in three consecutive steps. First, in the pre-analysis, codes like FastQC (Babraham Bioinformatics, 2016) and MultiQC [61] quality-check the reads. Second, in the analysis stage, the most computationally intensive one, software packages like LMAT [21], Kraken [41], CLARK [39], Centrifuge [7], and CLARK-S [40] (see S3 Fig for details) classify the reads taxonomically or functionally. Finally, in the post-analysis step, different tools like Krona [42], Pavian [62], or Recentrifuge further process the results to enable more in-depth analysis and improved visualization. (PDF) [file pcbi.1006967.s002.pdf]

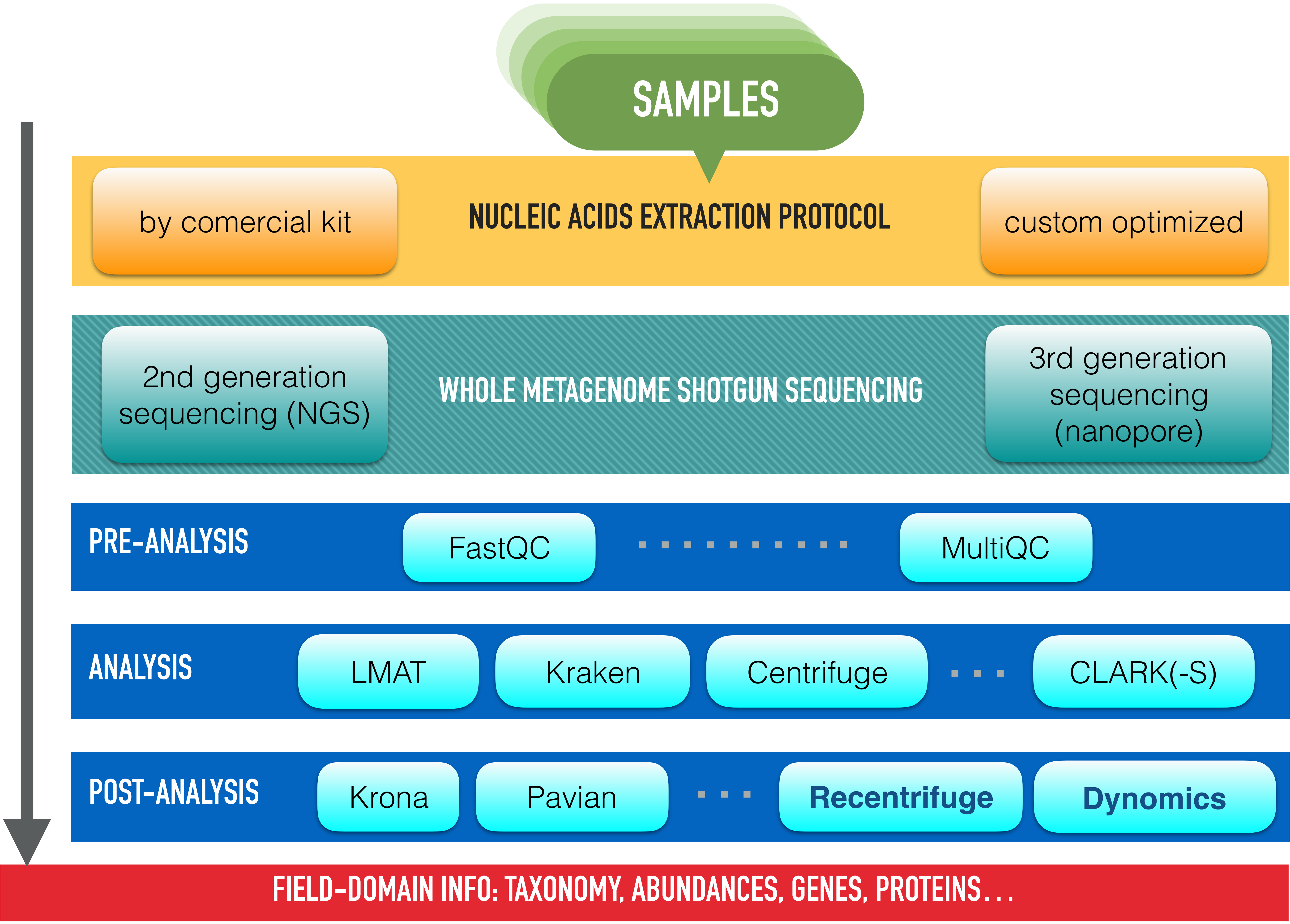
SAMPLES
by comercial kit
NUCLEIC ACIDS EXTRACTION PROTOCOL
custom optimized
2nd generation sequencing (NGS)
WHOLE METAGENOME SHOTGUN SEQUENCING
3rd generation sequencing (nanopore)
PRE-ANALYSIS
FastQC
MultiQC
ANALYSIS
LMAT
Kraken
Centrifuge
CLARK(-S)
POST-ANALYSIS
Krona
Pavian
Recentrifuge
Dynomics
FIELD-DOMAIN INFO: TAXONOMY, ABUNDANCES, GENES, PROTEINS…
