# Supplementary material for: Recentrifuge: Robust comparative analysis and contamination removal for metagenomics
Source: PLoS Comput Biol. 2019 Apr 8;15(4):e1006967. doi: 10.1371/journal.pcbi.1006967 (PMC6472834; doi:10.1371/journal.pcbi.1006967)
Supplement: S3 Fig — The core phase of a metagenomics analysis pipeline (see S1 and S2 Figs for the outline of the bioinformatic phases) is carried out by high performance computing software. These are intensive codes in both CPU and memory (sometimes, they are input/output intensive too), such as LMAT [21], Kraken [41] and, more recently, CLARK-S [40] and Centrifuge [7]. All these tools are performing taxonomic classification and abundance estimation, whereas LMAT is also able to annotate genes. For the taxonomic classification, both LMAT and Kraken use an exact k-mer matching algorithm with large databases (~100 GiB) while Centrifuge use compression algorithms to reduce the databases size (~10 GiB) but at some speed expense. CLARK-S use discriminative spaced k-mers to improve the sensitivity but with a toll on the performance. The most complete LMAT database is approaching half terabyte of required memory while the Centrifuge database generated in-house in March 2018 from the NCBI Nucleotide [63]nt database (~170 GiB) occupied just 105 GiB. The equivalent spaced k-mers database of CLARK-S generated in May 2018 took 267 GiB of disk space. (PDF) [file pcbi.1006967.s003.pdf]

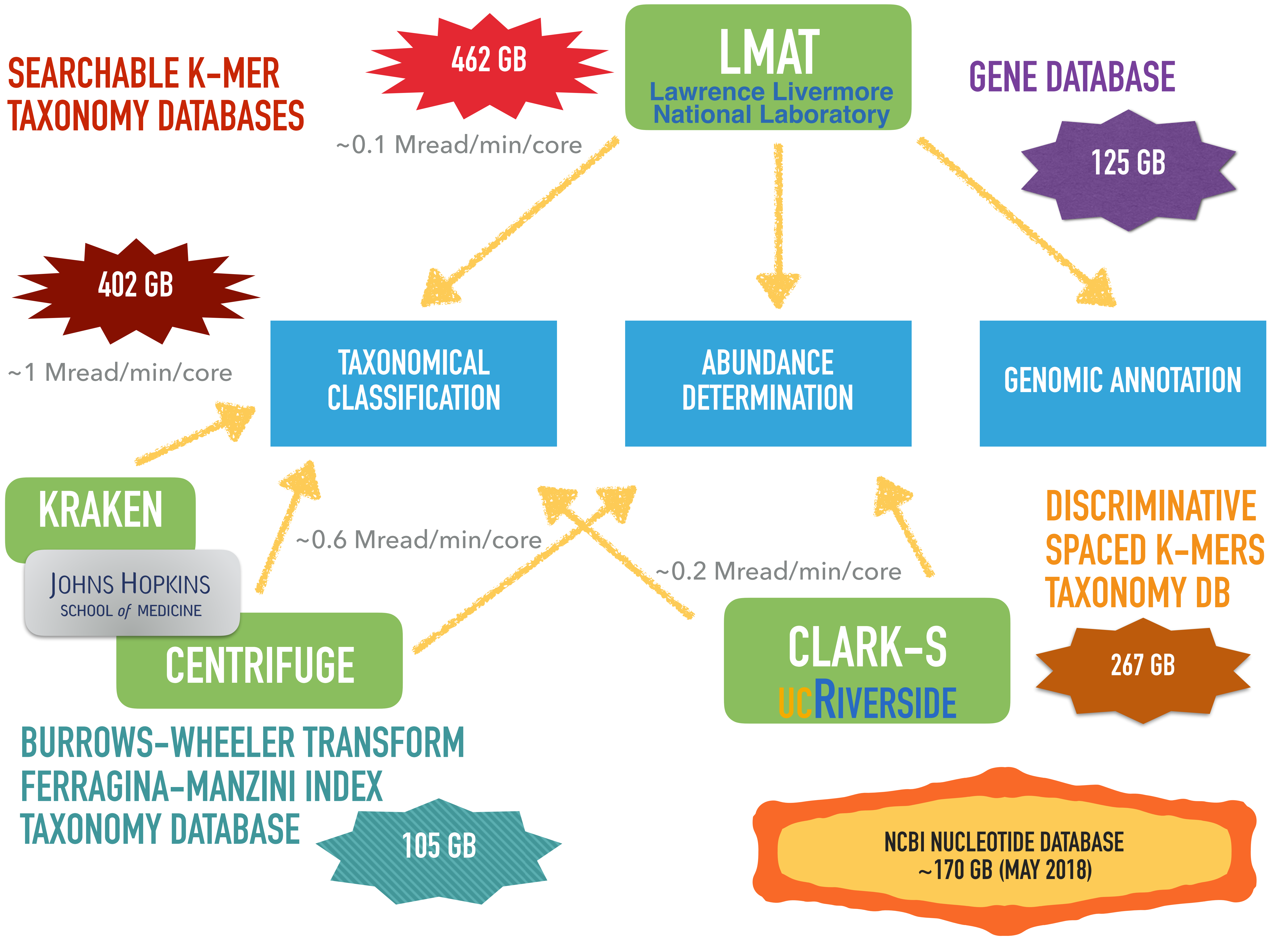
SEARCHABLE K-MER
TAXONOMY DATABASES
462 GB
~0.1 Mread/min/core
LMAT
Lawrence Livermore
National Laboratory
GENE DATABASE
125 GB
402 GB
~1 Mread/min/core
TAXONOMICAL
CLASSIFICATION
ABUNDANCE
DETERMINATION
GENOMIC ANNOTATION
KRAKEN
JOHNS HOPKINS
SCHOOL of MEDICINE
~0.6 Mread/min/core
~0.2 Mread/min/core
DISCRIMINATIVE
SPACED K-MERS
TAXONOMY DB
CENTRIFUGE
CLARK-S
UCRIVERSIDE
267 GB
BURROWS-WHEELER TRANSFORM
FERRAGINA-MANZINI INDEX
TAXONOMY DATABASE
105 GB
NCBI NUCLEOTIDE DATABASE
~170 GB (MAY 2018)
