# Supplementary material for: Recentrifuge: Robust comparative analysis and contamination removal for metagenomics
Source: PLoS Comput Biol. 2019 Apr 8;15(4):e1006967. doi: 10.1371/journal.pcbi.1006967 (PMC6472834; doi:10.1371/journal.pcbi.1006967)
Supplement: S4 Fig — This figure summarizes the immediate benefits of applying Recentrifuge to a study involving SMS of different but related samples, including negative controls (see S1 Fig). Recentrifuge generates four different sets of scored charts for each taxonomic level of interest in addition to the scored plots for the raw samples: samples with the control taxa subtracted, the exclusive taxa per sample and the shared taxa with and without control taxa subtracted. This battery of analysis and plots permits robust comparative analysis of multiple samples in low microbial biomass metagenomic studies. (PDF) [file pcbi.1006967.s004.pdf]

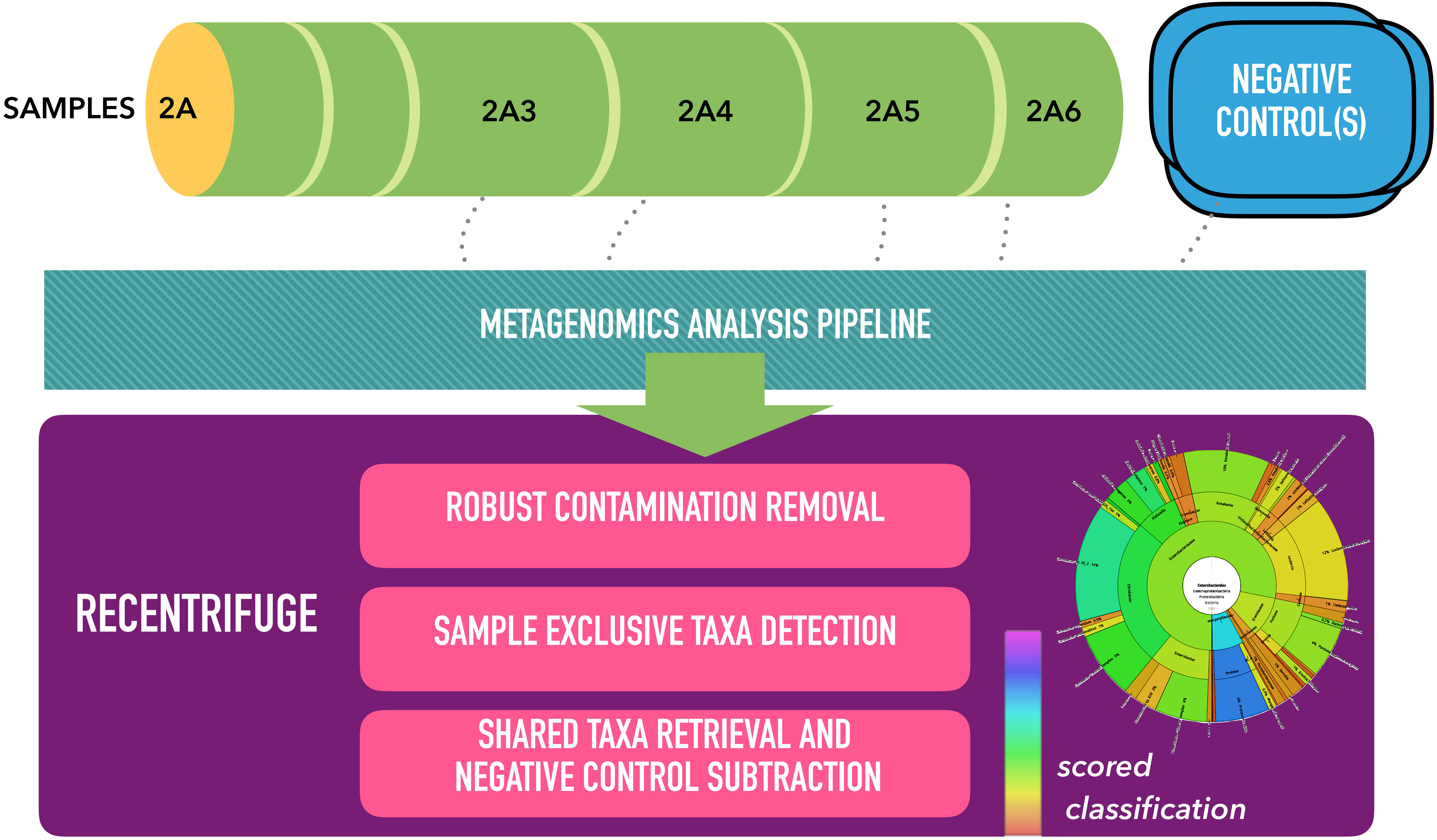
SAMPLES
2A
2A3
2A4
2A5
2A6
NEGATIVE CONTROL(S)
METAGENOMICS ANALYSIS PIPELINE
RECENTRIFUGE
ROBUST CONTAMINATION REMOVAL
SAMPLE EXCLUSIVE TAXA DETECTION
SHARED TAXA RETRIEVAL AND NEGATIVE CONTROL SUBTRACTION
scored classification
