# Supplementary material for: Recentrifuge: Robust comparative analysis and contamination removal for metagenomics
Source: PLoS Comput Biol. 2019 Apr 8;15(4):e1006967. doi: 10.1371/journal.pcbi.1006967 (PMC6472834; doi:10.1371/journal.pcbi.1006967)
Supplement: S7 Fig — Screenshots of the Recentrifuge web interface showing results for samples with paired-ends sequences for the batch 1 of the SMS study of plasma in ME/CFS patients [48]. The 1st batch samples with paired-ends sequences are two negative controls (samples S018 and S036) and the positive control (sample S008) with hMPV. In the figure, the top chart plots the control sample S018 (Ctrl1_S018_B1_Neg), highlighting the hMPV contamination. The bottom chart shows the summary sample for the positive control after the robust contamination removal (S008_B1_MPV_CTRL_SUMMARY), which kept the hMPV reads although hMPV contaminates the negative control S018. The filtering parameters for Recentrifuge were selected for allowing only high-scored taxa, with minscore of 75. (PDF) [file pcbi.1006967.s007.pdf]

Negative control sample S018 (Ctrl1_S018_B1_Neg)

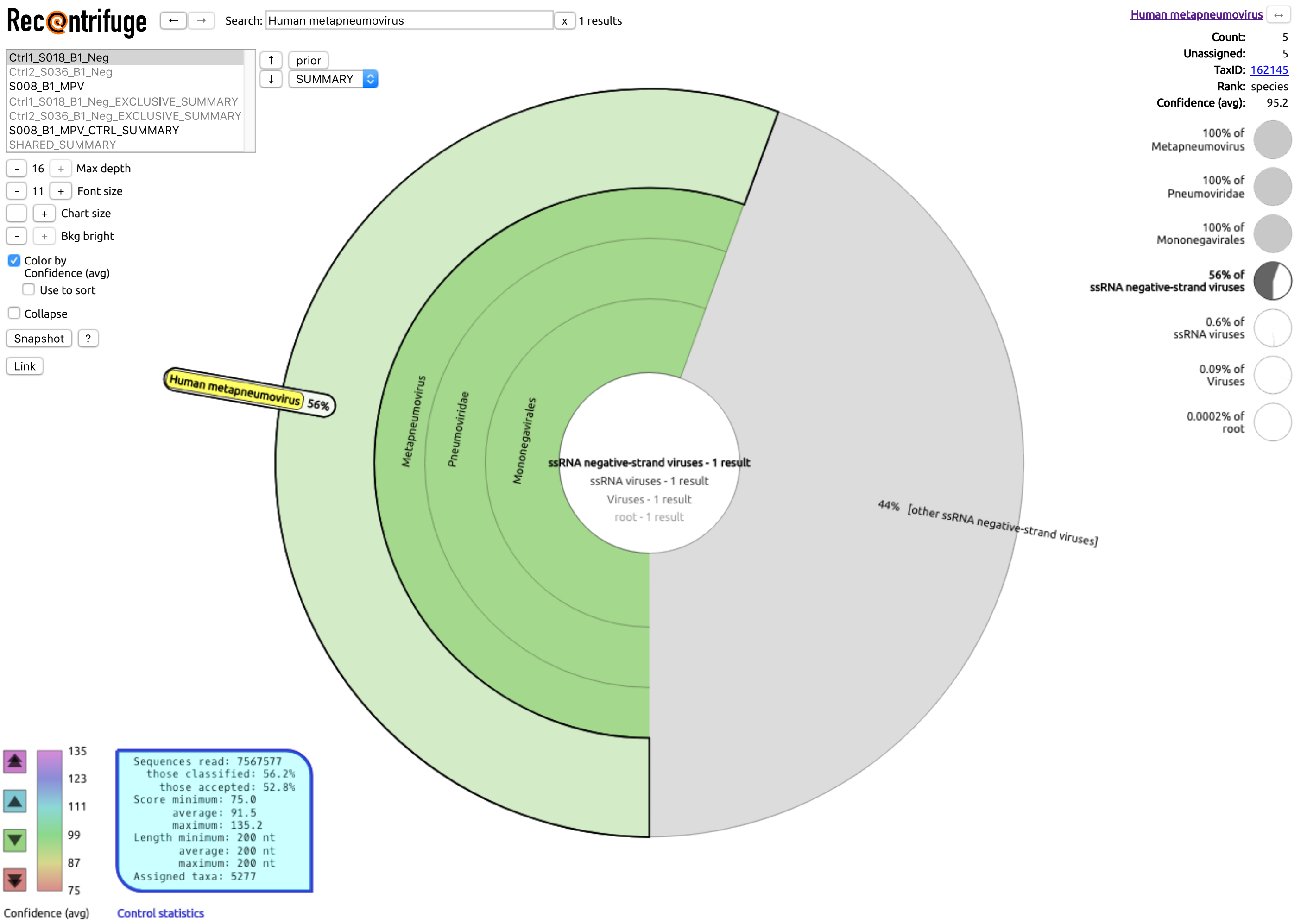

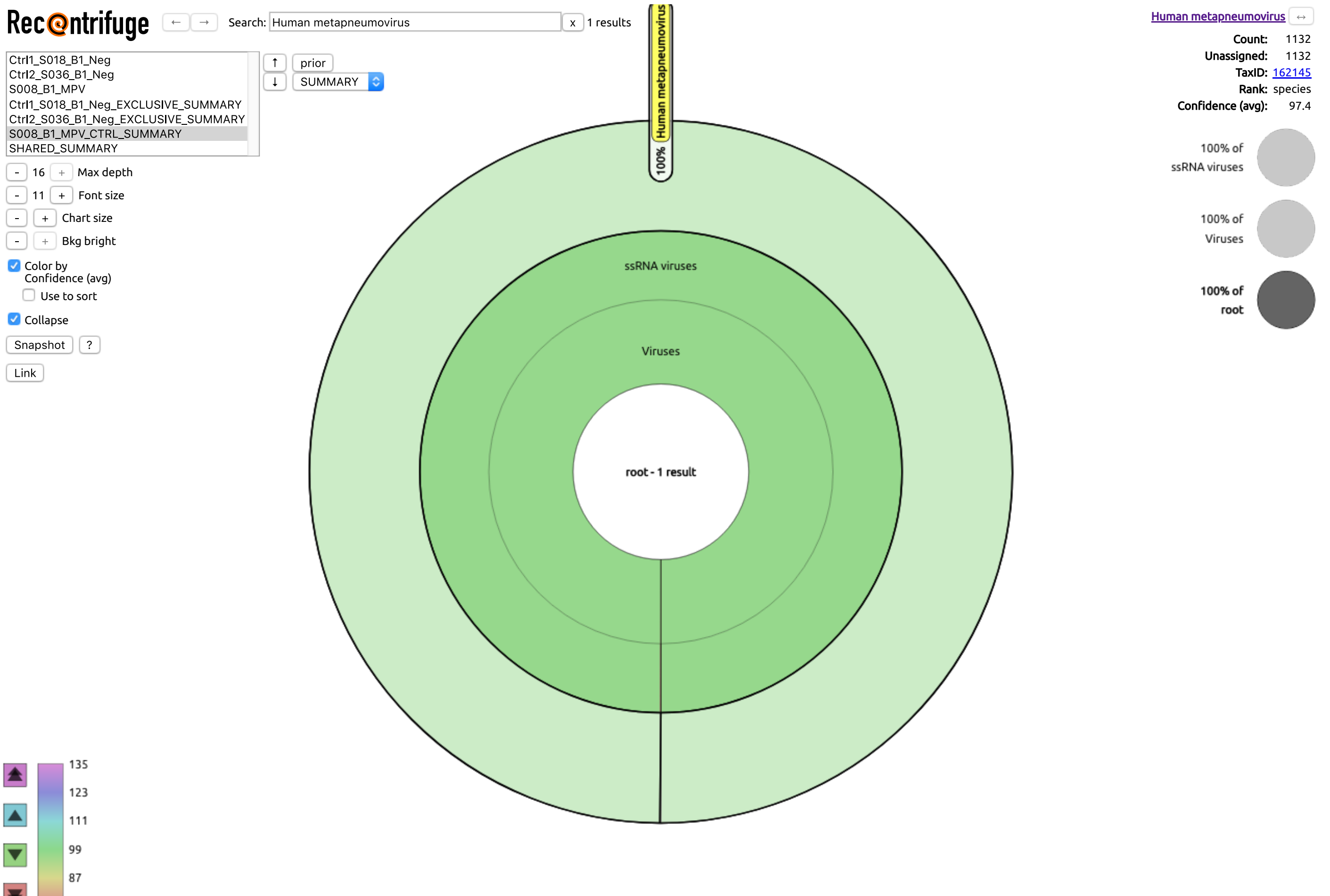

Positive control sample S018 after robust contamination removal (S008_B1_MPV_CTRL_SUMMARY)
