# Supplementary material for: Recentrifuge: Robust comparative analysis and contamination removal for metagenomics
Source: PLoS Comput Biol. 2019 Apr 8;15(4):e1006967. doi: 10.1371/journal.pcbi.1006967 (PMC6472834; doi:10.1371/journal.pcbi.1006967)
Supplement: S1 Appendix — (PDF) [file pcbi.1006967.s014.pdf]

Recentrifuge S1 Appendix

Computing kernel implementation details

Jose Manuel Martí*

March 24, 2019

The following list abridges some implementation details concerning the Recentrifuge computing kernel:

1. Coded using python multi-platform parallelization to reduce the elapsed time when dealing with massive datasets. Depending on the algorithm, the code parallelizes by input file, by taxonomic level or by derived sample —in the summary generation step.

2. Intensive use of recursive methods to cope with tree-arithmetics, which enables robust comparison between taxonomic trees at any rank. The code recognizes the 32 different taxonomic levels used in the NCBI taxonomy (see **S5 Fig**) [1]. Recentrifuge can adequately deal with arbitrary taxonomic levels (`NO_RANK`) in between the named NCBI taxonomic ranks, as happens with some complex eukaryotic taxa.

3. Software engineered with robustness as one of the leading targets. It is a full statically annotated Python 3.6 code. PEP-8 [2], PEP-484 [3], and PEP-526 [4] compliant. Written following the Google python style guide [5]. Checked with mypy and Pylint, Recentrifuge v0.28.7 code has been rated at 9.8/10.

4. Implemented under an object-oriented paradigm to ease future extensions targeting new or improved uses, Recentrifuge can be easily extended to understand additional input formats and other taxonomies different from NCBI —by direct support extending the base class or indirectly by using a

* Contact: jose.m.marti@uv.es

mapping software like CrossClassify [6]. **S6 Fig** shows a summarized UML (Unified Modeling Language) class diagram of Recentrifuge that exposes the main classes developed and currently used in the package.

It is worth to mention that the code allows applying a different parameter set to the control samples, including `mintaxa` and `minscore`. This feature is helpful when the control samples are too different from the ordinary ones and thus require unique values for the parameters that define how Recentrifuge treats the sample data. S1App-1 Table shows basic code layout statistics about Recentrifuge (source files, lines, and number of code lines equivalent in third generation languages) provided by cloc (v.1.76).

S1App-1 Table. Recentrifuge code layout. Basic code layout statistics about Recentrifuge v0.28 (source files, lines, and number of code lines equivalent in third generation languages) provided by cloc (v.1.76)

Language	files	blank	comment	code	×	scale	=	3rd gen. equiv
Python	26	612	1040	5288	×	4.20	=	22209.60
JavaScript	1	930	532	5116	×	1.48	=	7571.68
SUM	27	1542	1572	10404	×	2.86	=	29781.28

References

1. National Center for Biotechnology Information (1988) Taxonomy. [Internet]. National Library of Medicine (Bethesda, MD, USA). URL: https://www.ncbi.nlm.nih.gov/taxonomy/.
2. Rossum GV, Warsaw B, Coghlan N (2013) PEP-8: Style Guide for Python Code. [Internet]. Python Software Foundation. URL: https://www.python.org/dev/peps/pep-0008/.
3. Rosum GV, Lehtosalo J, Langa L (2015) PEP-484: Type Hints. [Internet]. Python Software Foundation. URL: https://www.python.org/dev/peps/pep-0484/.
4. González R, House P, Levkivskyi I, Roach L, Rosum GV (2016) PEP-526: Syntax for Variable Annotations. [Internet]. Python Software Foundation. URL: https://www.python.org/dev/peps/pep-0526/.

5. Patel A, Picard A, Jhong E, Hylton J, Smart M, Shields M (2017) Google Python Style Guide. [Internet]. Version 2.59. Google. URL: https://google.github.io/styleguide/pyguide.html.

6. Balvočiūtė M, Huson DH (2017) SILVA, RDP, Greengenes, NCBI and OTT — how do these taxonomies compare? BMC Genomics 18. ID: Balvočiūtė2017: 114. ISSN: 1471-2164. DOI: 10.1186/s12864-017-3501-4. URL: https://doi.org/10.1186/s12864-017-3501-4.
