# Supplementary material for: Recentrifuge: Robust comparative analysis and contamination removal for metagenomics
Source: PLoS Comput Biol. 2019 Apr 8;15(4):e1006967. doi: 10.1371/journal.pcbi.1006967 (PMC6472834; doi:10.1371/journal.pcbi.1006967)
Supplement: S2 Appendix — (PDF) [file pcbi.1006967.s015.pdf]

Recentrifuge S2 Appendix

Bioinformatics in the ME/CFS plasma study before Recentrifuge

Jose Manuel Martí *

March 24, 2019

We downloaded the paired-ends sequences from the NCBI SRA, 841 GiB in FASTQ files, which constituted 240 Gbp (giga-base-pairs) in 2.4 billion reads of raw genomic data distributed in 67 samples (see S2App-1 Table for details). Host subtraction was performed using bowtie2 v2.3.4.1 with –very-sensitive flag [1] against a superset of the *hg19* human reference, adding human mtRNA and human RNA. The unmapped reads were extracted using SAMtools [2] v1.7-3 with SAM filter flag to 13 and using HTSlib v1.7-6. They compounded 95 Gbp of raw genomic data, so 39% of the initial data.

Centrifuge [3] —release 1.0.3, Feb 2018— performed the taxonomic classification of the paired-end unmapped sequences of the 67 samples. The database for Centrifuge was generated using the procedure detailed in the Centrifuge-nt page of the Recentrifuge wiki (NCBI *nt* downloaded in March 2018) with the addition of the viral sequences from the NCBI WGS database [4] downloaded in March 2018, for a total database size of 170 GiB. The centrifuge-build step took more than 42 hours using 64 IBM Power9 cores at 3.1 GHz of an IBM fat-node equipped with one tebibyte shared memory, which was filled to a maximum of 93%. The Centrifuge database size generated had 105 GiB. Centrifuge run using the 32 cores of a Bull HPC shared-memory node equipped with Intel Haswell-based Xeon processors at 2.3 GHz, sharing half a tebibyte of DRAM memory. This fat-node stored the Centrifuge database on a PCIe SSD card (1.1 GiB of NVRAM). The flags passed to Centrifuge were **--min-hitlen 20** and **-k 1**, for an LCA (lowest common ancestor) strategy.

* Contact: jose.m.marti@uv.es

The output from Centrifuge was redistributed in datasets according to the sequencing batch and groups of illnesses and controls [5], as shown in S2App-1 Table.

S2App-1 Table. Samples in the SMS study of plasma in individuals with ME/CFS [5].

Group	N	N (single)	N (paired)	N (batch 2)	N (batch 3)	Declared	Repetitions	N
Lupus	13	6	7	5	2	11	2	13
CFS	32	7	25	11	14	25	7	32
Lyme	15	12	3	1	2	13	2	15
Healthy	34	10	24	11	13	25	9	34
MPV	1	0	1	0	0	1	0	1
Negative	7	0	7	4	1	7	0	7
SUM	102	35	**67**	32	32	82	20	102

References

1. Langmead B, Trapnell C, Pop M, Salzberg SL (2009) Ultrafast and memory-efficient alignment of short DNA sequences to the human genome. Genome biology 10: R25.
2. Li H, Handsaker B, Wysoker A, Fennell T, Ruan J, Homer N, Marth G, Abecasis G, Durbin R (2009) The sequence alignment/map format and SAMtools. Bioinformatics 25: 2078–2079.
3. Kim D, Song L, Breitwieser FP, Salzberg SL (Dec. 2016) Centrifuge: rapid and sensitive classification of metagenomic sequences. English. Genome Research 26: 1721–1729. ISSN: 1088-9051. DOI: 10.1101/gr.210641.116. URL: http://genome.cshlp.org/content/26/12/1721.
4. Wheeler DL, Barrett T, Benson DA, Bryant SH, Canese K, Chetvernin V, Church DM, DiCuccio M, Edgar R, Federhen S, Geer LY, Kapustin Y, Khovayko O, Landsman D, Lipman DJ, Madden TL, Maglott DR, Ostell J, Miller V, Pruitt KD, Schuler GD, Sequeira E, Sherry ST, Sirotkin K, Souvorov A, Starchenko G, Tatusov RL, Tatusova TA, Wagner L, Yaschenko E (2007) Database resources of the National Center

for Biotechnology Information. English. Nucleic acids research 35: D12. ISSN: 0305-1048. DOI: 10.1093/nar/gkl1031. URL: http://www.ncbi.nlm.nih.gov.

5. Miller RR, Uyaguari-Diaz M, McCabe MN, Montoya V, Gardy JL, Parker S, Steiner T, Hsiao W, Nesbitt MJ, Tang P, Patrick DM (2016) Metagenomic Investigation of Plasma in Individuals with ME/CFS Highlights the Importance of Technical Controls to Elucidate Contamination and Batch Effects. English. PLoS One 11: e0165691. ISSN: 1932-6203. DOI: 10.1371/journal.pone.0165691.
