# Supplementary material for: Recentrifuge: Robust comparative analysis and contamination removal for metagenomics
Source: PLoS Comput Biol. 2019 Apr 8;15(4):e1006967. doi: 10.1371/journal.pcbi.1006967 (PMC6472834; doi:10.1371/journal.pcbi.1006967)
Supplement: S3 Appendix — This appendix contains the most probable sources of translocation into the blood of other microbial taxa found [64–68]. (PDF) [file pcbi.1006967.s016.pdf]

Recentrifuge S3 Appendix

Other microbial taxa found with possible sources of translocation into the blood in the SMS study of plasma for ME/CFS patients [1]

Jose Manuel Martí*

March 24, 2019

S3App-1 Table. Other microbial taxa found with different possible sources of translocation into the blood.

**Phylum/Group**	**Taxa**	**Samples**	**Most probable source of translocation into blood**
Actinobacteria	*Rothia dentocariosa*	56/Lym<br>70/Hea<br>74/Hea	The buccal microbiota [2]
	Gardnerella vaginalis	51/Hea<br>90/Hea<br>101/Lup	Genitourinary microbiota [3]
Firmicutes	*Clostridium perfringens*	101/Lup (excl.)	Gut microbiota [4]
ssRNA virus	*Pegivirus C*	64/Hea<br>93/Hea	Refers to GBV-C/HGV in the genus *Pegivirus* of persistent GB viruses [5]
Basidiomycota	*Rhodosporidiobolus*	53/Lup (excl.)	The oral mycobiota [6]

In the samples column, those listed are the most representative in relative frequency. Besides, exclusiveness is indicated in parentheses, and the specimen group is after the slash: *Hea* for healthy individuals, *CFS* for ME/CFS patients, *Lym* for those suffering ADCLS, and *Lup* for those afflicted by SLE.

* Contact: jose.m.marti@uv.es

References

1. Miller RR, Uyaguari-Diaz M, McCabe MN, Montoya V, Gardy JL, Parker S, Steiner T, Hsiao W, Nesbitt MJ, Tang P, Patrick DM (2016) Metagenomic Investigation of Plasma in Individuals with ME/CFS Highlights the Importance of Technical Controls to Elucidate Contamination and Batch Effects. English. PLoS One 11: e0165691. ISSN: 1932-6203. DOI: 10.1371/journal.pone.0165691.
2. Jenkinson HF (2011) Beyond the oral microbiome. Environmental microbiology 13: 3077–3087. ISSN: 1462-2912. DOI: 10.1111/j.1462-2920.2011.02573.x.
3. Hardy L, Jespers V, Bulck Van den, Buyze J, Mwambarangwe L, Musengamana V, Vaneechoutte M, Crucitti T (2017) The presence of the putative *Gardnerella vaginalis* sialidase A gene in vaginal specimens is associated with bacterial vaginosis biofilm. Plos One 12: e0172522. DOI: 10.1371/journal.pone.0172522.
4. Rumah KR, Linden J, Fischetti VA, Vartanian T (2013) Isolation of *Clostridium perfringens* Type B in an Individual at First Clinical Presentation of Multiple Sclerosis Provides Clues for Environmental Triggers of the Disease. PLoS One 8: e76359. ISSN: 1932-6203. DOI: 10.1371/journal.pone.0076359.
5. Stapleton JT, Foung S, Muerhoff AS, Bukh J, Simmonds P (2011) The GB viruses: a review and proposed classification of GBV-A, GBV-C (HGV), and GBV-D in genus *Pegivirus* within the family *Flaviviridae*. Journal of General Virology 92: 233–246. DOI: //doi.org/10.1099/vir.0.027490-0.
6. Diaz PI, Hong BY, Dupuy AK, Strausbaugh LD (2017) Mining the oral mycobiome: Methods, components, and meaning. Virulence 8: 313–11. ISSN: 2150-5594. DOI: 10.1080/21505594.2016.1252015.
