# Supplementary material for: Recentrifuge: Robust comparative analysis and contamination removal for metagenomics
Source: PLoS Comput Biol. 2019 Apr 8;15(4):e1006967. doi: 10.1371/journal.pcbi.1006967 (PMC6472834; doi:10.1371/journal.pcbi.1006967)
Supplement: S4 Appendix — (PDF) [file pcbi.1006967.s017.pdf]

Recentrifuge S4 Appendix
Recentrifuge User Manual

Jose Manuel Martí*

March 24, 2019

Contents

1 Installation of Recentrifuge **5**

1.1 Step-by-step installation 5

1.1.1 Basic requirements 5

1.1.2 Getting the code 5

1.1.3 Getting the databases 6

1.1.4 Testing (optional step) 6

1.1.5 Ready! 6

1.1.6 Interactive visualization 6

1.1.7 Numeric results for downstream applications 7

1.2 Notes 7

1.2.1 Python version 7

1.2.2 Support for LMAT plasmids classification 7

2 Testing Recentrifuge **8**

2.1 Introduction 8

* Contact: jose.m.marti@uv.es

2.2 Automatic testing procedure . . . 8
2.2.1 Results from robust contamination removal . . . 8
2.2.2 Optional advanced procedures . . . 10
2.2.3 Other useful flags . . . 10
2.2.4 Test used for continuous integration (CI) . . . 10
2.3 The mock community . . . 10
2.4 Advanced: Step-by-step procedure . . . 11
2.4.1 Prerequisites . . . 11
2.4.2 Executing Remock . . . 12
A text file per sample . . . 12
An excel with all the samples . . . 13
2.4.3 Executing Recentrifuge . . . 14
2.4.4 Validation . . . 14
2.5 Understanding the messages of the robust contamination removal algorithm . . . 15
2.5.1 Format and meaning of the messages . . . 15
2.5.2 Application to the test dataset . . . 16

3 Running Recentrifuge **21**
3.1 Running Recentrifuge for Centrifuge . . . 21
3.1.1 Quick start . . . 21
3.1.2 File format . . . 21
3.1.3 Scoring schemes . . . 21
3.1.4 Advanced example . . . 22
3.2 Running Recentrifuge for LMAT . . . 23
3.2.1 Quick start . . . 23
3.2.2 Automatic detection of LMAT multiple output . . . 23
3.2.3 File format . . . 24
3.2.4 More about plasmids . . . 24
3.2.5 Scoring scheme . . . 25
3.2.6 Advanced example . . . 25

3.3 Recentrifuge for CLARK 26
3.3.1 Quick start 26
3.3.2 File format 26
3.3.3 Scoring schemes 27
3.3.4 Advanced example 28
3.4 Recentrifuge for Kraken 28
3.4.1 Quick start 28
3.4.2 File format 29
3.4.3 Scoring schemes 29
3.4.4 Advanced example 30
3.5 Recentrifuge for a generic classifier 31
3.5.1 Taxonomic classifiers with direct support 31
3.5.2 Requisites for generic support 31
3.5.3 Format for generic support: Overview 31
3.5.4 Format for generic support: Details 32
3.5.5 Format for generic support: Examples 32
CSV files 32
TSV files 33
SSV files 33
3.5.6 Quick start running 33
3.5.7 Scoring schemes 33
3.5.8 Advanced example 34

4 Running Rextract **35**
4.1 Overview 35
4.2 Details 35

5 Recentrifuge command line **37**
5.1 Command layout 37
5.2 Groups of options and flags 37

5.2.1 Input . . . 37
5.2.2 Output . . . 38
5.2.3 Tuning . . . 39
5.2.4 Fine tuning . . . 39
5.2.5 Advanced . . . 40
5.2.6 Other . . . 40

6 Troubleshooting 41

6.1 Mismatch between NCBI database versions . . . 41
6.2 Number of total accumulated reads is less than the accepted reads . . . 42
6.3 Where have those taxa with scarce reads assigned gone? . . . 42
6.4 Issues with LMAT plasmids . . . 43

List of Figures 44

References 44

1 Installation of Recentrifuge

1.1 Step-by-step installation

1.1.1 Basic requirements

Python 3.6 is required. If you need help with the installation and setup of Python 3.6 or higher, please consult Python Setup and Usage. No modules beyond Python Standard Library ones are used by Recentrifuge with the exception of *biopython* and, optionally:

- *pandas* for exporting results to CSV or TSV as extra files or for testing Recentrifuge.
- *openpyxl* package is also required, additionally, for *pandas* to export results in Excel format.
- *matplotlib* and *xlrd* are needed in addition to the previous packages for comprehensive testing the Recentrifuge package.

1.1.2 Getting the code

- **Option 1 (recommended)**: Get the Recentrifuge PyPI package.

```
$ pip install recentrifuge
```

To test Recentrifuge or to be able to export results to CSV, TSV, or Excel, you will also need:

```
$ pip install pandas openpyxl xlrd matplotlib
```

- **Option 2**: Clone the Recentrifuge repository on GitHub. Please see cloning a repository in GitHub for help on this. You will need `git` installed on your system, in case it is not, please check installing Git.
  - In case you need help with *biopython* installation, have a look at biopython installation instructions.
  - Should you need help installing *pandas* or *openpyxl*, please check pandas installation instructions or openpyxl installation instructions.

1.1.3 Getting the databases

In the cloning dir, execute `retaxdump.py`. It will download and unzip the required local databases from NCBI servers under the subdirectory `taxdump`. For the importance of keeping the NCBI database updated, please check S4 Appendix Section 6.1. For LMAT plasmids support please read S4 Appendix Section 1.2.2.

1.1.4 Testing (optional step)

Recentrifuge development is tested by an automatic continuous integration system (check Recentrifuge's Travis CI page for details). Please see comprehensive instructions about testing and validating your Recentrifuge installation in S4 Appendix Section 2.

1.1.5 Ready!

At this point, **Recentrifuge is ready** to analyze your samples:

- if you have results from Centrifuge, please see running Recentrifuge for Centrifuge at S4 Appendix Section 3.1
- in case you have LMAT outputs, your choice is running Recentrifuge for LMAT at S4 Appendix Section 3.2,
- if you have full-mode CLARK, CLARK-l, or CLARK-S results, please enter running Recentrifuge for CLARK flavors at S4 Appendix Section 3.3,
- in case you have data from Kraken, please check running Recentrifuge for Kraken at S4 Appendix Section 3.4,
- if you use any other taxonomic classifier, please see running Recentrifuge for a generic classifier at S4 Appendix Section 3.5.

1.1.6 Interactive visualization

Just open the HTML file generated by Recentrifuge with any JavaScript-enabled browser. Firefox or Chrome are recommended.

1.1.7 Numeric results for downstream applications

Recentrifuge generates CSV/TSV extra files or an Excel file with various sheets containing diverse statistics and detailed numeric results useful for downstream applications.

1.2 Notes

1.2.1 Python version

Python version under 3.6 is no supported as Recentrifuge uses new syntax features of Python 3.6, like syntax for variable annotations, PEP 526 [1] and formatted string literals PEP 498 [2]. The syntax for type annotations was introduced in Python 3.5 PEP 484 [3], but it is with Python 3.6 when it has achieved maturity for variable annotations. Powerful tools for static type analysis in Python have evolved along with these standards. The development of Recentrifuge includes checks with pylint and mypy. A code whose aim is to perform robust comparative metagenomic analysis is a very good candidate for robust coding.

1.2.2 Support for LMAT plasmids classification

One of the most interesting but still quite unknown features of the LMAT software is its ability to properly classify over 4000 plasmids. These plasmids are assigned a taxonomical id (`taxid`) beyond the NCBI system. Recentrifuge offers support for this extended classification, but requires the LMAT provided file `plasmid.names.txt` located in the same directory as the NCBI nodes information files. This location is controlled by the flag **-n/--nodespath**.

If Recentrifuge finds the `plasmid.names.txt` file, it will parse the plasmids taxid and name using ad hoc regular expressions in order to present the user with a meaningful name for the very diverse plasmids. Recentrifuge is also doing a check to assure that every plasmid in the file is compatible with the NCBI taxonomy used, so that only those passing are added. Further details in S4 Appendix Section 3.2.4.

2 Testing Recentrifuge

2.1 Introduction

A code like Recentrifuge that emphasizes robustness in coding and results requires a robust testing procedure. Recentrifuge includes an automatic testing utility called `retest` that tests the other components of the package: `retaxdump`, `remock`, recentrifuge (`rcf`), and `rextract`. The test dataset included with Recentrifuge is designed to efficiently check and challenge various algorithms in the code, such as the robust contamination removal and the comparative metagenomics analysis. This dataset also triggers crossover contamination detection and removal. The results of the testing are straightforwardly interpreted.

2.2 Automatic testing procedure

After the installation of Recentrifuge from GitHub or PyPI, the automatic testing procedure is simply invoked with:

```
$ retest
```

with '$' indicating the correspondent shell prompt. In typical use, the program performs different tests on the components of Recentrifuge package. If all the tests are passed, retest exits with code 0. An exit code different from 0 indicates the number of the testing stage that has failed. The flowchart of S4App-1 Fig shows the main workflow of `retest`, including the relation of the different exit values to the testing steps.

2.2.1 Results from robust contamination removal

Beyond the testing of every component of Recentrifuge, `retest` analyzes the performance of Recentrifuge's robust contamination removal algorithm using specific mock data optimized for this purpose. This test produces ROC (receiver operating characteristic) plots (see below) and also a comparison of abundance histograms (see Fig 6 of the main manuscript), similar to the following:

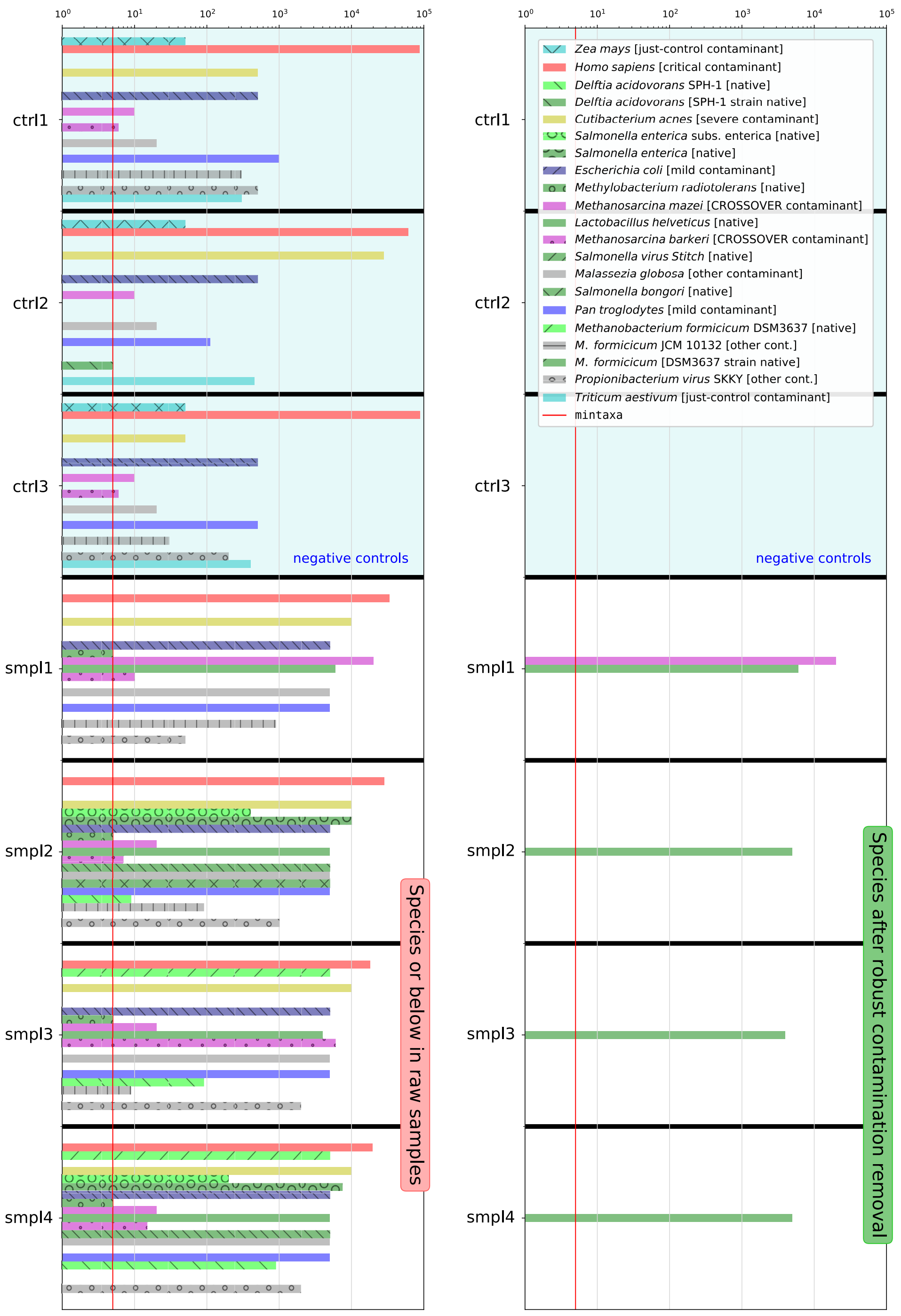

The left column of the figure shows the abundance histogram for some taxa in the dataset (7 raw samples: 4 samples plus 3 negative control samples) before the robust contamination removal algorithm goes into action. The right column shows the results after the algorithm intervention. Native taxa are green-colored, and crossover contaminants are colored in purple. The complete color code is detailed below, in S4 Appendix Section 2.5.1.

2.2.2 Optional advanced procedures

Additionally, `retest` includes some advanced procedures to obtain some graphics resulting from the check on specific features of the package:

- If the flag **-r** or **--roc** is activated, `retest` performs additional tests and generates a ROC figure such as S4App-2 Fig.
- The flag **-m** or **--mintaxa** enables additional tests for analyzing the dependency on the `mintaxa` parameter. This study is time-consuming since it runs `rcf` in a loop with many different (forced) values of `mintaxa`. The flag **-s** (or **--skip**) permits to reuse the results from a previous complete execution of `retest` with the **-m** flag, thus directly loading and analyzing the multiple results previously generated by `rcf`. This analysis produces a ROC plot like the shown in S4App-3 Fig.

2.2.3 Other useful flags

In `retest`:

- If the **-d** or **--debug** flag is enabled, `retest` produces a more verbose output.
- If the **-i** or **--ignore** option is active, the code continues testing even if errors arise.
- If the **-l** or **--local** flag is enabled, `retest` checks the Recentrifuge scripts in the local directory (instead of the pip installed ones).

2.2.4 Test used for continuous integration (CI)

Recentrifuge uses Travis CI to ensure that any new change or improvement in the code do not break it or inadvertently change its behavior or results. Please see the Recentrifuge's Travis CI page. Travis CI runs the following command for each new commit or tag:

```
./retest -d -l -r
```

2.3 The mock community

S4App-4 Fig details the design of the mock community, which has been carefully devised to challenge the robust contamination removal algorithm of Recentrifuge. The community contains diverse contaminant and

native taxa. The role is indicated by a characteristic background color as shown in the legend of S4App-4 Fig. That color code is also observed by the detailed output of the robust contamination removal algorithm. Red, yellow and navy blue indicate, respectively, critical, severe, and mild contaminants. A purple background indicates crossover contaminants (those contaminating the samples except the source sample, where they are native). A green background characterizes native taxa, while the grey one stands for other contaminants. Spread over different orders of magnitude, the abundances are fine-tuned to challenge Recentrifuge algorithms and easily detect any problem during the testing. In the top worksheet in S4App-4 Fig, the black rectangles surround the areas covering the control specimens with native taxa spiked by low abundances to simulate statistical noise in negative control samples such as low-frequency misclassifications and sequencing errors. The synthetic dataset includes an additional sample (`smplH`) that contains the 241 species and proportions of a high-complexity dataset used as a gold standard for benchmarking metagenomic software [4]. As with other samples, this specimen combined contaminants as additional taxa. The NCBI TaxId numbers of the 242 species were automatically retrieved using Entrez [5].

The taxa of the mock community cover the different domains of life and are mainly located at the taxonomic level of species or below, but there are also taxa belonging to other more general levels. Some taxa are intentionally introduced together to check Recentrifuge performance under difficult conditions. For instance, two strains of the archaea *Methanobacterium formicicum* were introduced: one native to the samples (*M. formicicum* DSM 3637) and another a contaminant (*M. formicicum* JCM 10132).

`Retest` triggers the parsing of the data in S4App-4 Fig by `remock` to create the mock dataset that `rcf` analyzes during its testing stage.

2.4 Advanced: Step-by-step procedure

Beyond `retest`, we encourage you to play with this manual procedure as we consider it is a good approach to get a deep insight into Recentrifuge's novel methods. Additionally, these steps are also useful for preparing lists of known contaminants (like a list of laboratory contaminants) for Recentrifuge to be aware.

2.4.1 Prerequisites

Let's start taking for granted that you have installed the code in `~/recentrifuge`, you have already used `retaxdump` to populate `./taxdump` and now you would like to test Recentrifuge. We will use `remock` to

generate the test samples with desired taxa and abundances, as previously determined.

2.4.2 Executing Remock

Remock works in two separate modes of input and two distinct modes of generating datasets, for a total of four different combined modes. The inputs could be either a text file per sample belonging to the dataset or an excel with the complete information for the dataset. The generation of the dataset can be with a random score or based on a specific file (a Centrifuge output file). Now, we will detail the two modes of remock covering random score generation. Test datasets are available as both text files and an excel file.

NOTE: Although either option works to perform this step of the testing, for the sake of compactness, we will continue the testing procedure with the dataset contained in the excel file.

A text file per sample In this example, remock will read the taxa and abundances from a collection of text files and use a random score generated with a minimum hit length (MHL) of 35. The text files should have an NCBI taxid, one per line, and its absolute abundance (counts) separated from the taxid by a tabulator. Comments are allowed with # at the beginning of the line. The following is an example of the beginning of a real file:

ctr1.mck
```
# CONTROL 1 MOCK LAYOUT FILE
# Homo sapiens
9606	600
# Cutibacterium acnes
1747	250
# E. coli
562 50
# Zea mays
4577	25
# Triticum aestivum
4565	3
(...)
```

The name of these files could be given to remock on a one-by-one basis, but for complex samples, it is more convenient just to pass a directory name to remock; remock will read all the *.mck files in the directory and generate the correspondent *.out (Centrifuge output) files. We will use the latter approach, and issue:

```
~/recentrifuge/remock -m ~/recentrifuge/test/ -r 35 -d
```

After loading the NCBI nodes and names files and populating internal data structures, remock will list and load the different mock files found in the directory provided after the **-m** argument. With the debugging

-d argument in the command line, for each file, `remock` will write to the console details about the data loaded:

```
Processing ~/recentrifuge/test/ctrl1.mck file:
600     reads for taxid  9606   ( Homo sapiens )
250     reads for taxid  1747   ( Cutibacterium acnes )
50  reads for taxid  562    ( Escherichia coli )
25  reads for taxid  4577   ( Zea mays )
(...)
Generating ~/recentrifuge/test/ctrl1.out file... 1000 reads OK!
```

An excel with all the samples In this case, `remock` will read the taxa and abundances from an excel file and also use a random score generated with a minimum hit length (MHL) of 35. The excel file has a first row with the labels of the samples. The first column is merely informative, and its label should be `RECENTRIFUGE MOCK`. The second column contains the different taxids, and its label is `TAXID`. The last row is discarded and could contain useful info for the validation, such as the accumulated absolute frequencies per sample. This required format is more evident with a real example:

	A	B	C	D	E	F	G	H	I
1	RECENTRIFUGE MOCK	TAXID	ctrl1	ctrl2	ctrl3	smpl1	smpl2	smpl3	smpl4
2	*Homo sapiens*	9606	87130	60576	87130	34171	28324	18270	20396
3	*Cutibacterium acnes*	1747	500	28000	50	10000	10000	10000	10000
4	*Pan troglodytes*	9598	1000	110	500	5000	5000	5000	5000
5	*Escherichia coli*	562	500	500	500	5000	5000	5000	5000
6	*Zea mays*	4577	50	50	50	4	4	4	4
7	*Triticum aestivum*	4565	300	450	400	0	0	0	0
8	*Malassezia globosa CBS 7966*	425265	0	200	1000	5000	5000	5000	5000
9	*Propionibacterium phage SKKY*	1655020	500	0	200	50	1000	2000	2000
10	*Methanosarcina mazei*	2209	10	10	10	20000	20	20	20
11	*Methanosarcina barkeri*	2208	6	4	6	10	7	6001	15
12	*Lactobacillus*	1578	4	100	150	500	5000	5000	5000
13	*Lactobacillus helveticus*	1587	0	0	4	6000	5000	4000	5000
14	*T5virus*	187218	0	0	0	4260	5240	4700	4860
15	*Salmonella virus Stitch*	1540099	0	0	0	0	5000	0	5000
16	*Salmonella enterica*	28901	0	0	0	0	10000	0	7500
17	*Salmonella bongori*	54736	0	0	0	0	5000	0	0
18	*Salmonella enterica subsp. enterica*	59201	0	0	0	0	400	0	200
19	*Methylobacterium radiotolerans*	31998	0	0	0	5	5	5	5
20	*Delftia acidovorans SPH-1*	398578	0	0	0	0	0	5000	5000
21	*Delftia*	80865	0	0	0	0	0	10000	5000
22	*Comamonadaceae*	80864	0	0	0	0	0	10000	5000
23	*Root*	1	10000	10000	10000	10000	10000	10000	10000
24	SUM (this row is ignored)		100000	100000	100000	100000	100000	100000	100000
25									

In this case, to ease the testing, the background colors of each taxon are related to the colors used in the debugging messages of the robust contamination removal algorithm. The name of the excel files is passed to `remock` using the **-x** argument:

```
~/recentrifuge/remock -x ~/recentrifuge/test/mock.xlsx -r 35 -d
```

Again, after loading the NCBI nodes and names files and populating internal data structures, since the debugging **-d** argument is present in the command line, `remock` will detail the data loaded:

```
         ctrl1  ctrl2  ctrl3  smpl1  smpl2  smpl3  smpl4
TAXID
9606     87130  60576  87130  34171  28324  18270  20396
1747       500  28000     50  10000  10000  10000  10000
9598      1000    110    500   5000   5000   5000   5000
562        500    500    500   5000   5000   5000   5000
(...)
1        10000  10000  10000  10000  10000  10000  10000
Generating ~/recentrifuge/test/ctrl1.out file... 100000 reads OK!
Generating ~/recentrifuge/test/ctrl2.out file... 100000 reads OK!
(...)
Generating ~/recentrifuge/test/smpl4.out file... 100000 reads OK!
```

2.4.3 Executing Recentrifuge

Recentrifuge keeps the relative path of the samples, which is convenient in large repositories of metagenomic datasets. In this case, we will obtain the results relative to the test path, so we move to the testing directory and issue the following commands:

```
cd ~/recentrifuge/test
~/recentrifuge/rcf -f . -o myTEST.rcf.html -c 3 -y 35 -d
```

The **-y** argument (`minscore`) is optional, but we choose it here to be the value of the **-r** argument of `remock`, i.e., the MHL of the randomly generated score. If such value is higher than the chosen MHL, Recentrifuge will filter the reads with less score. We can use the **-m** argument (`mintaxa`) to disregard the automatic setting and force a specific value for the minimum taxa to avoid collapsing one level to the parent one. Please, see the Recentrifuge main paper for further details and examples of both `minscore` and `mintaxa`.

2.4.4 Validation

The execution of Recentrifuge with the above command generates an HTML file (`myTEST.rcf.html`), an excel file (`myTEST.rcf.xlsx`), and detailed console output. The validation

- Interactively, loading the HTML file in a JavaScript-enabled browser, and comparing the results in the hierarchical pie plots with the validation HTML file,

- Numerically, loading the excel file in a spreadsheet program and comparing with the results contained in the validation Excel file,
- Procedurally, by directly comparing the console output to the validation console output. For this to be feasible, the **-d** argument should be present in the command line, so that `recentrifuge` will print details and debugging information. Please see the next subsection for hints to understand and validate the detailed output of the robust contamination removal algorithm.

2.5 Understanding the messages of the robust contamination removal algorithm

2.5.1 Format and meaning of the messages

The **-d** in the `recentrifuge` command line triggers detailed output and debugging information to the console. This is also true for the contamination removal algorithm, which prints details for the specific taxa selected as contaminants. For each taxon, this information can include the type of contaminant, the values of the thresholds of the crossover tests (`lims`), the relative frequency of the taxon in each sample of the study (`relfrec`), and the detection of crossovers with the source samples, if any.

In text consoles supporting color, the output is colored with different meanings, for example, the name of the taxon is colored according to the type of contaminant. The colors, types of contaminants and rules are the following:

Critical	`if all relfreq > SEVR_CONTM_MIN_RELFREQ`
Severe	`if any relfreq > SEVR_CONTM_MIN_RELFREQ`
Mild	`if all relfreq > MILD_CONTM_MIN_RELFREQ`
Just CTRL	`not present in samples, just in any control`
Crossover	`passes both checks of the crossover test`
Other	`not in a case above`
NO contamination	`not in any control sample (beyond mintaxa)`

where the default values for the parameters are:

```
SEVR_CONTM_MIN_RELFREQ: float = 0.01
MILD_CONTM_MIN_RELFREQ: float = 0.001
```

These and other parameters of the robust contamination removal algorithm are loaded from the file `recentrifuge/params.py` so that the user can easily modify their values from their defaults.

The two values after `lims` are the limits imposed on the taxon relative frequency by both tests of the crossover check (colored in green): the first is from the outlier test, and the latter is from the order of magnitude test. After, the taxon relative frequency for the controls and other samples is shown.

Please see the Recentrifuge paper for a detailed discussion of the robust contamination removal algorithm, including the crossover check.

2.5.2 Application to the test dataset

In our test dataset, for the case of the taxonomic level of "species" we have something like (the precise output depends on the specific version of the mock dataset used):

```
Analysis for taxonomic rank "species":
(...)
Robust contamination removal: Searching for contaminants...
critical:    9606 Homo sapiens relfreq: [0.9, 0.6, 0.9][0.3, 0.3, 0.2, 0.2]
other cont:  1982305 Propionibacterium virus SKKY lims: [0.09]<[5] relfreq: [0.005, 0, 0.002][0.0005, 0.01, 0.02, 0.02]
mild cont:   562 Escherichia coli relfreq: [0.005, 0.005, 0.005][0.05, 0.05, 0.05, 0.05]
mild cont:   9598 Pan troglodytes relfreq: [0.01, 0.001, 0.005][0.05, 0.05, 0.05, 0.05]
crossover:   2209 Methanosarcina mazei lims: [0.002]<[0.1] relfreq: [0.0001, 0.0001, 0.0001][0.2, 0.0002, 0.0002, 0.0002]
             crossover: [T, F, F, F]
-> Include 2209 just in: smpl1
just-ctrl:   4565 Triticum aestivum relfreq: [0.003, 0.004, 0.004][0, 0, 0, 0]
other cont:  76773 Malassezia globosa lims: [0.08]<[1e+01] relfreq: [0, 0.002, 0.01][0.05, 0.05, 0.05, 0.05]
crossover:   2208 Methanosarcina barkeri lims: [0.0009]<[0.06] relfreq: [6e-05, 0, 6e-05][0.0001, 7e-05, 0.06, 0.0001]
             crossover: [F, F, T, F]
-> Include 2208 just in: smpl3
just-ctrl:   4577 Zea mays relfreq: [0.0005, 0.0005, 0.0005][0, 0, 0, 0]
severe:      1747 Cutibacterium acnes relfreq: [0.005, 0.3, 0.0005][0.1, 0.1, 0.1, 0.1]
```

In a console supporting color, the output is colored (the precise output depends on the specific version of the mock dataset used):

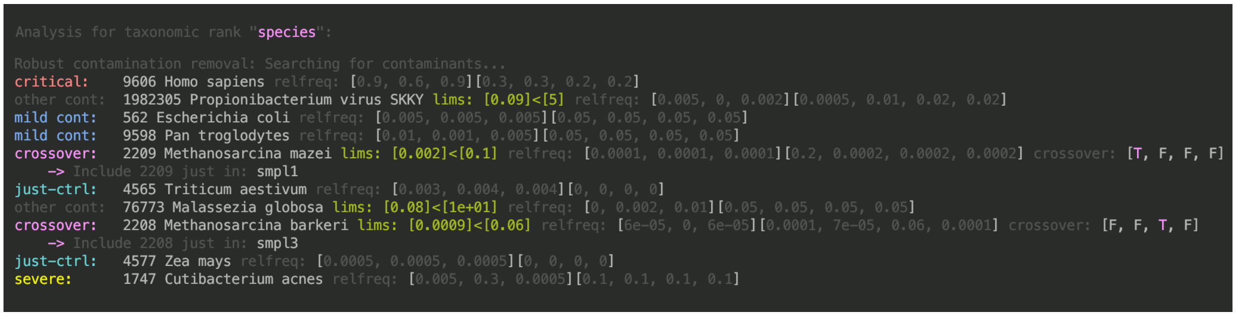

The taxa and colors of the detected contaminants should match the ones in the excel file of the test dataset, so we finally have:

- Critical contamination (in red): *Homo sapiens*.
- Severe contamination (in yellow): *Cutibacterium acnes*
- Mild contamination (in blue): *Escherichia coli* and *Pan troglodites*
- Contamination exclusive to controls (in cyan): *Triticum aestivum* and *Zea mays*
- Crossover contamination (in purple):
  - *Methanosarcina mazei*, with sample `smpl1` as source, and contaminating all the samples
  - *M. barkeri*, with sample `smpl3` as source, and contaminating all the samples except `ctrl2`.
- Other contamination (in grey): *Propionibacterium virus SKKY* and *Malassezia globosa*

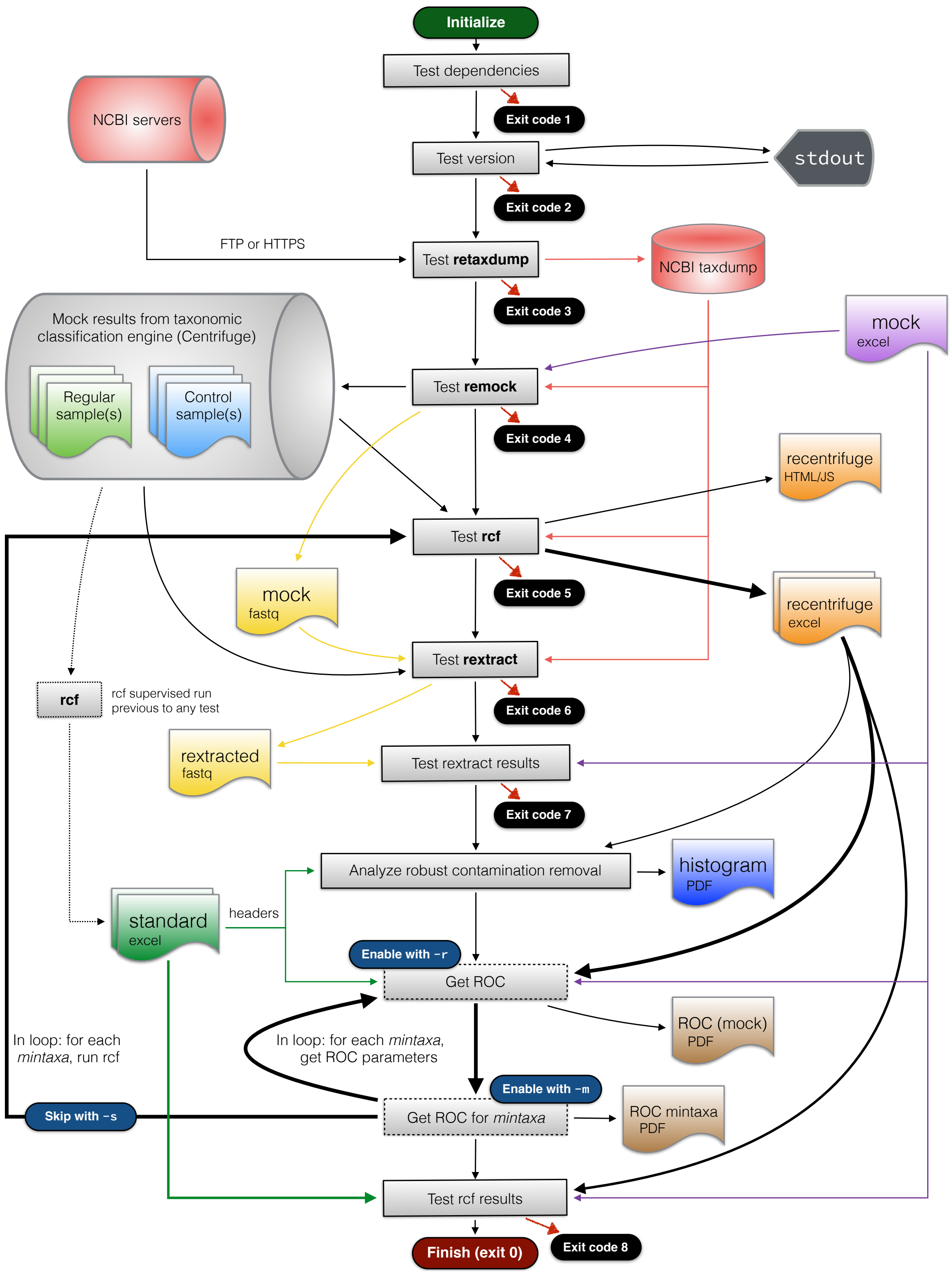

S4App-1 Fig. `Retest` **flowchart diagram.** The dotted lines indicate procedures previously completed to prepare the standard needed for comparisons in some stages of the testing workflow. The dashed lines denote optional procedures that are detailed in S4 Appendix Section 2.2.2.

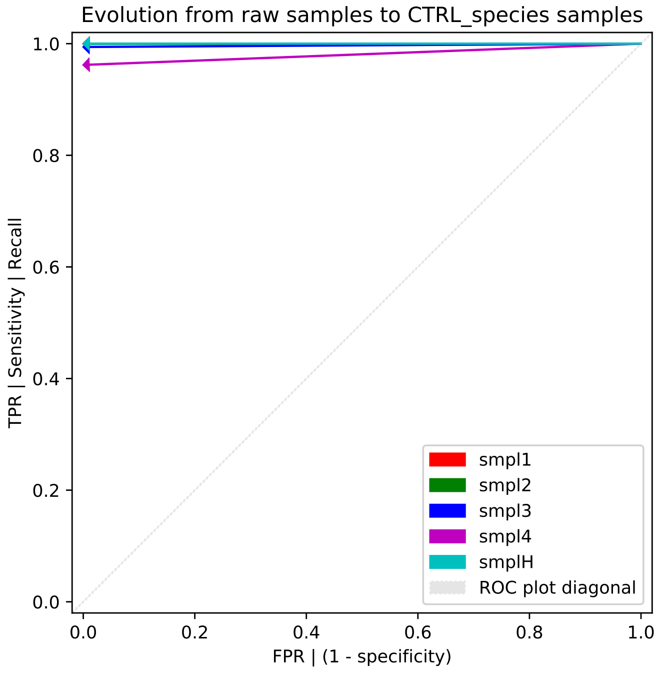

S4App-2 Fig. ROC generated by `retest` showing the impact of the robust contamination removal. The ROC (receiver operating characteristic) plot is using the test results of Recentrifuge and the information in the mock dataset to calculate the evolution of the sensitivity and specificity from the raw specimens to the CTRL_species samples.

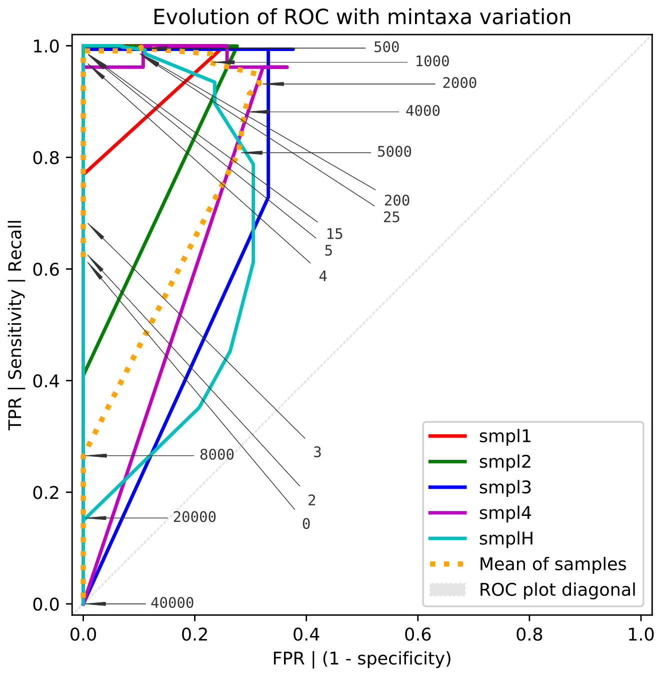

S4App-3 Fig. ROC generated by `retest` in order to study the dependence on the `mintaxa` parameter. The ROC (receiver operating characteristic) plot is using the test results of many executions of Recentrifuge and the information in the mock dataset to follow the evolution of the sensitivity and specificity of the CTRL_species samples when forcing different `mintaxa` values, some of them indicated by the numbers at the base of the arrows.

	A	B	C	D	E	F	G	H	I	J
1	RECENTRIFUGE MOCK	TAXID	ctrl1	ctrl2	ctrl3	smpl1	smpl2	smpl3	smpl4	smplH
2	*Homo sapiens*	9606	86405	60385	87710	33271	28225	18171	19496	40000
3	*Cutibacterium acnes*	1747	500	28000	50	10000	10000	10000	10000	10000
4	*Pan troglodytes*	9598	1000	110	500	5000	5000	5000	5000	5000
5	*Escherichia coli*	562	500	500	500	5000	5000	5000	5000	5000
6	*Zea mays*	4577	50	50	50	4	4	4	4	4
7	*Triticum aestivum*	4565	300	450	400	0	0	0	0	0
8	*Malassezia globosa*	76773	20	20	20	5000	5000	5000	5000	5000
9	*Propionibacterium virus SKKY*	1982305	500	0	200	50	1000	2000	2000	4000
10	*Methanosarcina mazei*	2209	10	10	10	20000	20	20	20	20
11	*Methanosarcina barkeri*	2208	6	4	6	10	7	6001	15	103
12	*Lactobacillus*	1578	4	100	150	500	5000	5000	5000	5000
13	*Lactobacillus helveticus*	1587	3	2	3	6000	5000	4000	5000	0
14	*T5virus*	187218	1	0	1	4260	5240	4700	4860	0
15	*Salmonella virus Stitch*	1540099	3	1	2	0	5000	0	5000	0
16	*Salmonella enterica*	28901	3	2	1	0	10000	0	7500	0
17	*Salmonella bongori*	54736	0	1	0	0	5000	0	0	0
18	*Salmonella enterica subsp. enterica*	59201	1	0	2	0	400	0	200	0
19	*Methanobacterium formicicum DSM 3637*	1204725	1	2	1	0	9	90	900	0
20	*Methanobacterium formicicum JCM 10132*	1300163	300	3	30	900	90	9	0	2995
21	*Methylobacterium radiotolerans*	31998	0	0	0	5	5	5	5	0
22	*Delftia acidovorans SPH-1*	398578	0	0	0	0	0	5000	5000	0
23	*Delftia*	80865	0	0	0	0	0	10000	5000	0
24	*Comamonadaceae*	80864	0	0	0	0	0	10000	5000	0
25	*Acidocella aminolytica*	33998	0	1	4	0	0	0	0	1097
26	*Actinokineospora diospyrosa*	103728	1	0	0	0	0	0	0	2610
27	*Actinokineospora globicatena*	103729	1	0	3	0	0	0	0	2051
262	*Williamsia deligens*	321325	2	1	2	0	0	0	0	615
263	*Williamsia maris*	72806	1	2	0	0	0	0	0	2336
264	*Williamsia serinedens*	391736	3	0	3	0	0	0	0	1448
265	*Yonghaparkia alkaliphila*	355930	3	1	0	0	0	0	0	1125
266	ROOT	1	10000	10000	10000	10000	10000	10000	10000	0
267	SUM (this row is ignored)		100000	100000	100000	100000	100000	100000	100000	600000

	A	B	C	D	E	F	G
1	TAXID	NAME	COLOR	HATCH	NATIVES	RANK	HIST
2	4577	*$\mathit{Zea\; mays}$ [just-control contaminant]*	c	"x"	[]	species	TRUE
3	9606	*$\mathit{Homo\; sapiens}$ [critical contaminant]*	r	""	[]	species	TRUE
4	398578	*$\mathit{Delftia\; acidovorans}$ SPH-1 [native]*	#00FF00	"\\"	["smpl3", "smpl4"]	no_rank_species	TRUE
261	72806	Williamsia maris	k	""	["smplH"]	species	FALSE
262	391736	Williamsia serinedens	k	""	["smplH"]	species	FALSE
263	355930	Yonghaparkia alkaliphila	k	""	["smplH"]	species	FALSE

TYPES OF CONTAMINANTS AND RULES

**Critical**	`if all relfreq > SEVR_CONTM_MIN_RELFREQ`
**Severe**	`if any relfreq > SEVR_CONTM_MIN_RELFREQ`
**Mild**	`if all relfreq > MILD_CONTM_MIN_RELFREQ`
**Just CTRL**	`not present in samples, just in any control`
**Crossover**	`passes both checks of the crossover test`
**Other**	`not in a case above`
**NO contamination**	`not in any control sample (beyond mintaxa)`
(black rectangle)	`statistical noise in negative control samples`
**Native high-complexity**	`native taxa from NatMet high-complexity dataset for metagenomics benchmarks (Scyrba et al., 2017)`

DEFAULT VALUES

```
SEVR_CONTM_MIN_RELFREQ  (float)     0.01
MILD_CONTM_MIN_RELFREQ  (float)    0.001
```

S4App-4 Fig. Rationale and design of the mock community. The synthetic community contains diverse contaminant and native taxa, whose precise role is indicated by the characteristic background color shown in the legend. For example, green background characterizes native taxa, while purple background indicates crossover contaminants (those contaminating the samples except the source sample, where they are native). Such color code is also observed by the detailed output of the robust contamination removal algorithm. The taxa are mainly species or below, but there are also taxa belonging to other more general levels. Spread over different orders of magnitude, the abundances are fine-tuned to challenge Recentrifuge algorithms and easily detect any problem during the testing. In addition, the sample `smplH` includes the 241 species and proportions of a high-complexity dataset used as a gold standard for benchmarking metagenomic software [4]. In the spreadsheet, the black rectangles surround the areas simulating statistical noise in negative control samples such as low-frequency misclassifications and sequencing errors. The constants shown in the legend are classification parameters of the robust contamination removal algorithm. `Retest` triggers the parsing of these worksheets by `remock` to create the mock dataset that `rcf` analyzes during its testing.

3 Running Recentrifuge

3.1 Running Recentrifuge for Centrifuge

3.1.1 Quick start

Let's suppose you have installed Recentrifuge with `pip` or cloned the repo in `~/recentrifuge`, and you would like to analyze and compare the Centrifuge output from samples *S1*, *S2* and *S3*, for instance. Provided you have already used `retaxdump` to populate `./taxdump`, the command would be:

```
~/recentrifuge/rcf -f S1.out -f S2.out -f S3.out
```

where you should exclude the `~/recentrifuge/` part for `pip` installations in this and future command-lines.

Sometimes you have a lot of samples and you just want to "recentrifuge" all of them. If they are in the directory `my_outputs_dir`, you achieve that with the following line:

```
~/recentrifuge/rcf -f my_outputs_dir
```

3.1.2 File format

Recentrifuge reads the direct output from Centrifuge and shows diverse descriptive statistics. For example, for a rare environmental sample "centrifuged" with minimum hit length (MHL) set to 35 and "recentrifuged" with MHL filtered to 50, the Recentrifuge console output is:

```
Loading output file EnvSample_mhl35_k1_cf.out... OK!
Seqs read: 2_828_017  [698.21 Mnt]
Seqs clas: 468_295    (83.44% unclassified)
Seqs pass: 314_510    (32.84% rejected)
Scores: min = 50.0, max = 207.3, avr = 105.4
Length: min = 80 nt, max = 302 nt, avr = 258 nt
2796 taxa with assigned reads
Building from raw data... EnvSample_mhl35_k1_cf sample OK!
Load elapsed time: 3.6 sec
```

3.1.3 Scoring schemes

There are different options to score the reads classified by Centrifuge, which could be selected with the option *-s/–scoring*. Recentrifuge supports the following scoring schemes for Centrifuge:

- **SHEL** (*Single Hit Equivalent Length*): This is a score value roughly equivalent to the length in pair bases of a single hit to the database. It is calculated as the square root of the Centrifuge score, plus 15. This is currently the default scoring scheme for Centrifuge data in Recentrifuge.
- **LENGTH**: The score of a read will be its length (or the combined length of mate pairs).
- **LOGLENGTH**: Logarithm (base 10) of the length score.
- **NORMA**: This score is the normalized score SHEL/LENGTH, so it takes into account both the assignment quality and the length of the read.

The last three scoring schemes are very useful when there are reads with a diverse order of magnitude in length, like in nanopore sequencing.

For every scoring scheme, the `minscore` parameter works for the calculated SHEL score of the read. So, for example, a `minscore` of 35 (indicated with the **-y 35** option) will filter the same reads independently of the scoring scheme selected.

3.1.4 Advanced example

Let's review a more complex example in depth: to analyze the Centrifuge output:

- from:
  1. Sample X1 (file `X1.nt_mhl30_k1_cf.out`)
  2. Sample X2 (file `X2.nt_mhl30_k1_cf.out`)
  3. Sample X3 (file `X3.nt_mhl30_k1_cf.out`)
- with ONE negative control (file `CTRL.nt_mhl30_k1_cf.out`),
- but excluding taxa assigned to Chordata (taxid 7711), Unclassified sequences (taxid 12908) and Other sequences (taxid 28384),
- with the taxonomy files downloaded to `/my/tax/dir`,
- and saving the output to `Xsamples.rcf.html` file (and to `Xsamples.rcf.xls`),

the command would be:

```
~/recentrifuge/rcf -n /my/tax/dir -c 1 -f CTRL.nt_mhl30_k1_cf.out -f X1.nt_mhl30_k1_cf.out
    -f X2.nt_mhl30_k1_cf.out -f X3.nt_mhl30_k1_cf.out -x 7711 -x 12908 -x 28384 -o Xsamples.rcf.html
```

3.2 Running Recentrifuge for LMAT

3.2.1 Quick start

Let's suppose you have installed Recentrifuge with `pip` or cloned the repo in `~/recentrifuge`, and you would like to analyze and compare the LMAT output from several samples. Provided you have used `retaxdump` to populate `./taxdump` and you have each sample in one different subdirectory under the current one, you can put Recentrifuge to work with just the following line:

```
~/recentrifuge/rcf -l .
```

where you should exclude the `~/recentrifuge/` part for `pip` installations in this and future command-lines.

If you would like to include LMAT plasmids classifications in your results, please add the LMAT file `plasmid.names.txt` to the `taxdump` directory as detailed in S4 Appendix Section 1.2.2.

3.2.2 Automatic detection of LMAT multiple output

The above-stated solution is Recentrifuge LMAT automatic mode: it searches for directories not starting with a dot under the current one and, in each of them, it will suppose that a set of LMAT output files resides (typically `Long.file.name.ending.in_output??.out`, where the `??` goes from 0 to the number of cores used by LMAT minus one). Then, Recentrifuge will load each sample in parallel, parsing sequentially the output files under each of those subdirectories. Obviously, this is the most convenient mode of operation of Recentrifuge for large sets of samples analyzed by LMAT, to avoid specifying them one by one.

In case you want to be more specific about the place of the samples to be processed, you can specify the directory that contains the output files for each sample. So, if the samples *S1*, *S2* and *S3*, for instance, are in the directories `./setA/S1`, `./setA/S2`, `./setB/S3`, respectively, the command would be:

```
~/recentrifuge/rcf -l ./setA/S1 -l ./setA/S2 -l ./setB/S3
```

Finally, if you happen to have several samples analyzed by LMAT in the same directory, you can select them one by one. For example, if the samples *S10*, *S11* and *S12* are together in the directory `./all_samples`, the command would be:

```
~/recentrifuge/rcf -l ./all_samples/S10 -l ./all_samples/S11 -l ./all_samples/S12
```

You can mix the two latter approaches, as Recentrifuge will detect if, for the LMAT output files to be parsed, you are selecting a whole directory or just a file prefix to be used inside a directory.

3.2.3 File format

Recentrifuge reads the direct outputs from LMAT and shows diverse descriptive statistics both about the reads and the LMAT classification process. Note that the output from LMAT is quite verbose and, in the case of large datasets, it could take some minutes to be processed by Recentrifuge. For example, for the LMAT analysis of the SRR829867 dataset using 32 cores and filtering reads classified with a LMAT score under -1, the Recentrifuge console output is:

```
Loading output file ~/SRR829867/SRR829867.fasta.lmat-4-14.20mer.db.lo.rl_output0.out...OK!
Loading output file ~/SRR829867/SRR829867.fasta.lmat-4-14.20mer.db.lo.rl_output1.out...OK!
(...)
Loading output file ~/SRR829867/SRR829867.fasta.lmat-4-14.20mer.db.lo.rl_output31.out...OK!
Seqs read: 23_025_264   [2.33 Gnt]
Seqs clas: 22_870_103   (0.67% unclassified)
Seqs pass: 7_245_243    (68.32% rejected)
DB Matching: Multi = 77.0%  Direct = 22.3%  ReadTooShort = 0.0%  LowScore = 0.0%  NoDbHits = 0.7%
Scores: min = -1.0, max = 2.8, avr = -0.5
Length: min = 101 nt, max = 101 nt, avr = 101 nt
4345 taxa with assigned reads
/Users/martijm/local/lmat-scripts/SRR829867 sample OK!
Load elapsed time: 347 sec
```

3.2.4 More about plasmids

If you would like to include LMAT plasmids classifications in your results, please add the LMAT plasmids file `plasmid.names.txt` to the `taxdump` directory as detailed in S4 Appendix Section 1.2.2. Recentrifuge will detect it and proceed to parse the plasmid data using regular expressions. Recentrifuge will output a summary for this parsing and for a sanity check of the plasmids data found:

```
Loading LMAT plasmids... OK!
Plasmid sanity check:  rejected (taxid error) = 286  rejected (parent error) = 2
Plasmid pattern matching:  1st type = 4066   2nd type = 284   other = 0
```

If the debug flag is active (**-d**), Recentrifuge will give details:

```
Plasmid taxid ERROR! Taxid=294 already a NCBI taxid. Declared parent is 294 but NCBI parent is 136843.
Plasmid details:  Pseudomonas fluorescens strain PC20 plasmid pNAH20,
                  complete sequence[sequence_id 814379][seq_data_id 7244447][tax_node_id 294]
Plasmid taxid ERROR! Taxid=1318 already a NCBI taxid.
                  Declared parent is 1318 but NCBI parent is 1301.
Plasmid details:  Streptococcus parasanguinis plasmid pFW213,
                  complete sequence[sequence_id 806688][seq_data_id 7037937][tax_node_id 1318]
(...)
```

```
Plasmid parent taxid ERROR! Taxid=2521 and parent=2521.
Plasmid details:  Plasmid pADB201 (from Mycoplasma mycoides),
                  complete genome[sequence_id 829559][seq_data_id 8131462][tax_node_id 2521]
(...)
```

3.2.5 Scoring scheme

Recentrifuge supports the specific scoring scheme of LMAT, which is the default and only choice for LMAT outputs. The **--minscore** parameter works for the final LMAT score of the read. LMAT classification scores between 0 and −1 are considered not very reliable, and under −1 the classifications should be used with caution.

3.2.6 Advanced example

As a more complete example, to analyse the the LMAT output:

- with the taxonomy files downloaded to `/my/tax/dir`,
- from samples X1 (in directory `./A/X1`), X2 (in directory `./B/X1`), X3 (in directory `./C/X3`) and X4 (in directory `./C/X4`),
- with ONE negative control (in directory `./CTRL`),
- excluding reads with a LMAT classification score under −1.0,
- excluding taxa (un)assigned to Cellular organisms (taxid 131567) and assigned to Chordata (taxid 7711),
- with the output Excel in the format of `Dynomics` (*in preparation*),
- and saving the output to `Xsamples.rcf.html` file,

the command would be:

```
~/recentrifuge/rcf -n /my/tax/dir -l ./CTRL -l ./A/X1 -l ./B/X2 -l ./C/X3 -l ./D/X4 -c 1
      -y ”-1.0” -x 131567 -x 7711 -e DYNOMICS -o Xsamples.rcf.html
```

3.3 Recentrifuge for CLARK

3.3.1 Quick start

Suppose you have installed Recentrifuge with `pip` and have used `retaxdump` to populate `./taxdump`. Now, you would like to analyze and compare the CLARK **full-mode** output from samples *S1*, *S2* and *S3*. In this case, the command would be:

```
rcf -r S1.csv -r S2.csv -r S3.csv
```

Often you have a dataset with a lot of samples and you just want to "recentrifuge" all of them. If they are in the directory `my_CLARK_outputs_dir`, you do that with the following line:

```
rcf -r my_CLARK_outputs_dir
```

3.3.2 File format

Recentrifuge currently supports **full-mode** output of CLARK, CLARK-l and CLARK-S, the spaced k-mers version of CLARK. As stated by the author, "*The full mode (i.e.,* **-m 0***) loads all discriminative k-mers in RAM and provides confidence score for all assignments. Thus, it offers high sensitivity.*"

CLARK **full-mode** results are in CSV format with 8 columns, including various scoring indexes: hit counts, gamma, and confidence. Recentrifuge shows statistics about all these CLARK scores for every analyzed sample, for example:

```
Loading output file Specimen_1.csv... OK!
  Seqs read: 20_183_483 [4.04 Gnt]
  Seqs clas: 18_405_324 (8.81% unclassified)
  Seqs pass: 17_412_044 (5.40% rejected)
  Hit (score): min = 1.0, max = 420.0, avr = 97.7
  Conf. score: min = 0.3, max = 1.0, avr = 0.7
  Gamma score: min = 0.0, max = 2.5, avr = 0.9
  Read length: min = 200 nt, max = 200 nt, avr = 200 nt
  4305 taxa with assigned reads
Building from raw data... Specimen_1 sample OK!
Load elapsed time: 97 sec
```

In order to analyze your CLARK results with Recentrifuge, please run your flavor of CLARK with the **-m 0** flag to select **full-mode**, thus enabling higher sensitivity and the scoring of the assignments needed by Recentrifuge to continuously evaluate the classifications confidence.

3.3.3 Scoring schemes

There are different options to score the reads classified by CLARK. Recentrifuge supports the following specific scoring schemes for CLARK, which could be selected with the option **-s/–scoring**.:

- **SHEL** (*Single Hit Equivalent Length*): This is a score value in pair bases roughly equivalent to a single hit to the database. In CLARK, between the two assignments, this is the hit count score result of the top or the classified one. This is currently the default scoring scheme for CLARK data in Recentrifuge.

- **CLARK_C**: This scoring scheme is only available for CLARK-S output. It takes the confidence score $\widehat{C}$ as the score for a read so that:
$$\widehat{C} = C = \frac{h1}{h1 + h2}$$
if the 1st assignment is classified, or:
$$\widehat{C} = 1 - C = \frac{h2}{h1 + h2}$$
in case the majority of a read is not classified (1st assignment unclassified). See CLARK's `README` file for details on how $h1$ and $h2$ are calculated. If you use this scoring, you will probably want to filter to a minimum of 0.5 (**-y 0.5**) or beyond, as under 0.5 the assignments have very low confidence.

- **CLARK_G**: This scheme scores every read with its CLARK gamma score, so it is only available for this classifier.

For each of those scoring schemes, the `minscore` parameter works for the statistics selected as the score: hit counts, confidence, and gamma, respectively. So, for example, a `minscore` of 1 (indicated with the **-y 1** option) for a *SHEL* scoring will not filter a single read, while for *CLARK_C* scoring no read will pass the filter.

Recentrifuge also supports the following generic scoring schemes for CLARK, which are useful when there are reads with a diverse order of magnitude in length, like in nanopore sequencing:

- **LENGTH**: The score of a read will be its length (or the combined length of mate pairs).

- **LOGLENGTH**: Logarithm (base 10) of the length score.

- **NORMA**: This score is the normalized score SHEL/LENGTH, so it takes into account both the assignment quality and the length of the read. Very useful when both the score assignments and lengths are variable among the reads.

3.3.4 Advanced example

Let's see a more complex example in detail. In order to analyze the **full-mode** output of any flavor of CLARK:

- with the taxonomy files downloaded to `/my/tax/dir`,
- from samples X1 (file `X1.csv`), X2 (file `X2.csv`) and X3 (file `X3.csv`),
- with two negative controls (files `CTRL1.csv` and `CTRL2.csv`),
- saving the output to `Xsamples.rcf.html` file,
- with the scoring referred to the confidence score per read (*CLARK_C*) calculated by CLARK's **full-mode**,
- filtering reads with 0.3 as a minimum value for such confidence,
- for the general samples (except negative controls), with 10 as the minimum number of reads assigned to one clade to avoid collapsing it to the parent clade,
- with 5 for this last parameter but referred to the control samples,
- and excluding the reads assigned to humans (taxid 9606),

the command would be:

```
rcf -n /my/tax/dir -r CTRL1.csv -r CTRL2.csv -r X1.csv -r X2.csv -r X3.csv -c 2
       -o Xsamples.rcf.html -s CLARK_C -y 0.3 -m 10 -w 5 -x 9606
```

3.4 Recentrifuge for Kraken

3.4.1 Quick start

Suppose you have installed Recentrifuge with `pip` and have used `retaxdump` to populate `./taxdump`. Now, you would like to analyze and compare the Kraken output from samples *S1*, *S2* and *S3*. In this case, the command would be:

```
rcf -k S1.krk -k S2.krk -k S3.krk
```

Often you have a dataset with a lot of samples and you just want to "recentrifuge" all of them. If they are in the directory `my_Kraken_outputs_dir`, you do that with the following line:

```
rcf -k my_Kraken_outputs_dir
```

3.4.2 File format

Recentrifuge supports Kraken version 1 and version 2 output for both single- and paired-end datasets. If you use the **-k** option to indicate a directory with the samples instead of individual samples, Recentrifuge will suppose that the Kraken samples are those files there ending with `.krk`. Please rename your samples ending with `.krk` if you plan to use the Recentrifuge autodetection of Kraken samples in a directory.

3.4.3 Scoring schemes

There are different options to score the reads classified by Kraken. Recentrifuge supports the following specific scoring schemes for Kraken, which could be selected with the option **-s/--scoring**.:

- **SHEL** (*Single Hit Equivalent Length*): This is a score value in pair bases roughly equivalent to a single hit to the database. In Kraken, this is calculated as the k-mer hit count of the top assignment, plus the default k-mer length in Kraken (35).
- **KRAKEN**: This scoring scheme is only available for this classifier. It divides the k-mer hit count of the top assignment by the total k-mers in the read and multiplies the result by 100 to give a percentage of coverage (the fraction of the read k-mers covered by k-mers belonging to the read final assignment). This is the default scoring scheme for Kraken samples, and it supports the mixing of samples with different read length.

For each of those scoring schemes, the `minscore` parameter works for the statistics selected as the score: SHEL or k-mer coverage. So, for example, a `minscore` of 40 (indicated with the **-y 40** option) for a *SHEL* scoring would filter those reads not hitting 40 nt, while for *KRAKEN* scoring would filter those reads with less than 40% k-mer coverage for the top assignment.

Recentrifuge also supports the following generic scoring schemes for Kraken, which are especially useful when there are reads with a diverse order of magnitude in length, like in nanopore sequencing:

- **LENGTH**: The score of a read will be its length (or the combined length of mate pairs).
- **LOGLENGTH**: Logarithm (base 10) of the length score.
- **NORMA**: This score is the normalized score SHEL / LENGTH, so it takes into account both the assignment quality and the length of the read. Very useful when both the score assignments and lengths are variable among the reads.

For these three scoring schemes, the `minscore` parameter works for the calculated SHEL score of the read. So, for example, a minscore of 35 (indicated with the **-y 35** option) will filter the same reads independently of the scoring scheme selected among these three.

3.4.4 Advanced example

Let's see a more complex example in detail. In order to analyze the Kraken output:

- with the taxonomy files downloaded to `/my/tax/dir`,
- from samples X1 (file `X1.krk`), X2 (file `X2.krk`) and X3 (file `X3.krk`),
- with two negative controls (files `CTRL1.krk` and `CTRL2.krk`),
- saving the output to `Xsamples.rcf.html` file,
- with the scoring referred to the hit k-mer coverage percentage (*KRAKEN*),
- filtering reads with 25% as a minimum value for such confidence,
- and excluding the reads assigned to humans (taxid 9606),

the command would be:

```
rcf -n /my/tax/dir -k CTRL1.krk -k CTRL2.krk -k X1.krk -k X2.krk -k X3.krk -c 2
       -o Xsamples.rcf.html -s KRAKEN -y 25 -x 9606
```

3.5 Recentrifuge for a generic classifier

3.5.1 Taxonomic classifiers with direct support

Recentrifuge includes direct support for results from Centrifuge, LMAT, CLARK, and Kraken. If you have data from these classifiers, please proceed, respectively to:

- S4 Appendix Section 3.1 for Centrifuge results.
- S4 Appendix Section 3.2 for LMAT results.
- S4 Appendix Section 3.3 for CLARK results.
- S4 Appendix Section 3.4 for Kraken results.

In any other case, proceed to the next subsection.

3.5.2 Requisites for generic support

One of the cornerstones of Recentrifuge's robust approach is to consider and propagate the confidence level of taxonomic assignments throughout the analysis. A well-known principle in Physics says "*every measurement with its uncertainty*," and Recentrifuge honors this. The confidence level of a taxonomic assignment scores it, thus indicating us how reliable it is. If we don't have a score, we lack a direct quantitative measure of the reliability that any taxon is really present in our samples.

In practice, this means that Recentrifuge only supports data from taxonomic classifiers providing any kind of confidence level for their assignments. Beyond that, as Recentrifuge works on a read-by-read basis, it requires primary information available for each read analyzed.

3.5.3 Format for generic support: Overview

Recentrifuge can parse files with comma-separated values (CSV), tab-separated values (TSV), or space-separated values (SSV), with a line per each sequenced read processed in a sample of the dataset. Per read/line Recentrifuge requires at least the following data:

- The NCBI taxonomic identifier if the assignment is positive, or any code in case of unclassified read.
- Any confidence level for the assignment.
- The length of the read.

3.5.4 Format for generic support: Details

The format of the data files is passed to Recentrifuge using the **--format** command-line option followed by a string, such as in **--format "TYP:TSV, TID:2, LEN:4, SCO:5, UNC:0"**. The string is composed for the next comma-separated fields with 3 letters indicating the name of the field and the value of the field separated by a colon. The fields are:

- The TYPe of file (TYP), and the accepted values are CSV, TSV or SSV.
- The column number for the NCBI Taxonomic IDentifier (TID).
- The column number for the LENgth (LEN) of the read in nucleotides, as an integer number.
- The column number for the SCOre (SCO) given to the assignments, which could be an integer or a real number.
- The code in the TID column that indicated that a read is UNClassified (UNC).

For the column numbers, the accepted values are positive integers, thus starting with 1. The order of the fields in the format string is arbitrary. If there are repeated fields, Recentrifuge attends to the last value. Spaces around commas and colons in the format string are tolerated.

When parsing the data files, Recentrifuge will remove double-quotes or spaces around the data, which is typically useful for some CSV formats. The values for the LENgth of reads are expected to be integers with the read length in nucleotides, while the SCOres given to the assignments can be integers or real numbers, even negative (as in LMAT). In any case, Recentrifuge assumes that lower values of score indicate lower confidence in the assignments.

3.5.5 Format for generic support: Examples

CSV files Example of TYPe of file CSV with data enclosed in double-quotes and header, Taxonomic IDs in the 3rd col, LENgth of reads in the 2nd col, SCOre of assignments in the 4th col, and the UNClassified code is `NA`. File head:

```
#label,length,taxid,score,read-type
"read01","200","9606","30","paired-end"
"read02","200","9606","31","paired-end"
"read03","200","9606","32","paired-end"
"read12","200","NA","-","paired-end"
```

The correspondent format string is **"TYP:CSV,TID:3,LEN:2,SCO:4,UNC:NA"** or equivalent (for instance, the string **"UNC: NA, TYP: csv, LEN: 2, TID: 3,SCO: 4"** is equivalent).

TSV files Example of TYPe of file TSV with no header, Taxonomic IDs in the 2nd col, LENgth of reads in the 4th col, SCOre of assignments in the 5th col, and the UNClassified code is `0`. File head:

```
RR01	9606	SEQEX2	111 50
RR02	9606	SEQEX2	111 51
RR03	0	SEQEX2	150 -
RR04	9606	SEQEX2	111 52
```

The correspondent format string is **"TYP:TSV,TID:2,LEN:4,SCO:5,UNC:0"** or equivalent.

SSV files Example of TYPe of file SSV with header, Taxonomic IDs in the 2nd col, LENgth of reads in the 4th col, SCOre of assignments in the 5th col, and the UNClassified code is `*`. File head:

```
label tid sequencer len score
READ01 9606 NANOPORE 1589 50
READ02 * - 244 -
READ03 9606 NANOPORE 2106 51
READ04 9606 NANOPORE 849 52
```

The correspondent format string is **"TYP:ssv,TID:2,LEN:4,SCO:5,UNC:*"** or equivalent.

3.5.6 Quick start running

Suppose you have installed Recentrifuge with `pip` and have used `retaxdump` to populate `./taxdump`. Now, you would like to analyze and compare the output from samples *S1*, *S2* and *S3* from a classifier that generates data with the format of the TSV example above. In such case, the command would be:

```
rcf --format "TYP:TSV,TID:2,LEN:4,SCO:5,UNC:0" -g S1.any -g S2.any -g S3.any
```

3.5.7 Scoring schemes

There are different options to score the reads classified by a generic taxonomic classifier. Recentrifuge supports the following scoring schemes for a generic classifier, which could be selected with the option **-s/--scoring.**:

- **GENERIC**: This scoring scheme is the default scheme used for a generic classifier and is not available for specific classifiers. It directly uses the scores given to the assignments in the data files, which can be integers or real numbers. Recentrifuge takes for granted that lower values of score indicate lower confidence in the taxonomic assignments.

Recentrifuge also supports the following general scoring schemes, which are especially useful when there are reads with a diverse order of magnitude in length, such as in nanopore sequencing:

- **LENGTH**: The score of a read will be its length (or the combined length of mate pairs).
- **LOGLENGTH**: Logarithm (base 10) of the length score.
- **NORMA**: This score is the normalized score GENERIC / LENGTH in percentage, so it takes into account both the assignment quality and the length of the read. Very useful when both the score assignments and lengths are variable among the reads.

For all these scoring schemes, the `minscore` parameter works for the direct score of the read assigned by the taxonomic classifier. So, for example, a minscore of 35 (indicated with the **-y 35** option) will filter the same reads independently of the scoring scheme selected.

3.5.8 Advanced example

Let's see a more complex example in detail. In order to analyze the Kraken output:

- with the taxonomy files downloaded to `/my/tax/dir`,
- of a taxonomic classifier of nanopore sequences with the SSV format of the example above,
- from samples X1 (file `X1.nnp`), X2 (file `X2.nnp`) and X3 (file `X3.nnp`),
- with two negative controls (files `CTRL1.nnp` and `CTRL2.nnp`),
- saving the output to `Xsamples.rcf.html` file,
- with the scoring referred to the normalized score by length percentage (*NORMA*),
- filtering reads with 20 as a minimum value for the score,
- and excluding the reads assigned to humans (taxid 9606),

the command would be:

```
rcf -n /my/tax/dir --format "TYP:ssv,TID:2,LEN:4,SCO:5,UNC:*" -g CTRL1.nnp -g CTRL2.nnp
      -g X1.nnp -g X2.nnp -g X3.nnp -c 2 -o nanoporeXsamples.rcf.html -s NORMA -y 20 -x 9606
```

4 Running Rextract

4.1 Overview

Rextract cherry-picks reads in the fastq input files in accordance with Centrifuge classification. If you have cloned the repo in ~/recentrifuge and, from your paired-ends FASTQ input files for sample *S1* (S1_R1.fastq and S1_R2.fastq), you would like to extract reads assigned to the phylum Cyanobacteria (NCBI taxId 1117) or below, the command would be:

```
~/recentrifuge/rextract -f S1.out -i 1117 -1 S1_R1.fastq -2 S1_R2.fastq
```

4.2 Details

The layout of the rextract command is:

```
usage: rextract [-h] [-V] [-d] -f FILE [-l NUMBER] [-m NUMBER] [-n PATH]
                [-i TAXID] [-x TAXID] [-y NUMBER] (-q FILE | -1 FILE)
                [-2 FILE]
```

with the following optional arguments:

```
  -h, --help            show this help message and exit
  -V, --version         show program's version number and exit
  -d, --debug           increase output verbosity and perform additional
                        checks
  -f FILE, --file FILE  Centrifuge output file.
  -l NUMBER, --limit NUMBER
                        Limit of FASTQ reads to extract. Default: no limit
  -m NUMBER, --maxreads NUMBER
                        Maximum number of FASTQ reads to search for the taxa.
                        Default: no maximum
  -n PATH, --nodespath PATH
                        path for the nodes information files (nodes.dmp and
```

```
                        names.dmp from NCBI
  -i TAXID, --include TAXID
                        NCBI taxid code to include a taxon and all underneath
                        (multiple -i is available to include several taxid).
                        By default all the taxa is considered for inclusion.
  -x TAXID, --exclude TAXID
                        NCBI taxid code to exclude a taxon and all underneath
                        (multiple -x is available to exclude several taxid)
  -y NUMBER, --minscore NUMBER
                        minimum score/confidence of the classification of a
                        read to pass the quality filter; all pass by default
  -q FILE, --fastq FILE
                        Single FASTQ file (no paired-ends)
  -1 FILE, --mate1 FILE
                        Paired-ends FASTQ file for mate 1s (filename usually
                        includes _1)
  -2 FILE, --mate2 FILE
                        Paired-ends FASTQ file for mate 2s (filename usually
                        includes _2)
```

In case of a single fastq file (not paired-ends) and with the taxonomy files downloaded to `/my/tax/dir`, the command to issue in the example of the overview section would be:

```
~/recentrifuge/rextract -n /my/tax/dir -f S1.out -i 1117 -q S1.fastq
```

5 Recentrifuge command line

5.1 Command layout

The layout of the Recentrifuge (rcf) command (ver. 0.28) is:

```
usage: rcf [-h] [-V] [-n PATH] [--format GENERIC_FORMAT]
           (-f FILE | -g FILE | -l FILE | -r FILE | -k FILE) [-o FILE]
           [-e OUTPUT_TYPE] [-c CONTROLS_NUMBER] [-s SCORING] [-y NUMBER]
           [-m INT] [-x TAXID] [-i TAXID] [-a] [-z NUMBER] [-w INT]
           [-u OPTION] [-t] [--nokollapse] [-d] [--sequential]
```

5.2 Groups of options and flags

5.2.1 Input

Define Recentrifuge input files and formats:

```
  -n PATH, --nodespath PATH
                        path for the nodes information files (nodes.dmp and
                        names.dmp from NCBI)
  --format GENERIC_FORMAT
                        Format of the output files from a generic classifier
                        included with the option -g. It is a string like
                        ”TYP:csv,TID:1,LEN:3,SCO:6,UNC:0” where valid file
                        TYPes are csv/tsv/ssv, and the rest of fields indicate
                        the number of column used (starting in 1) for the
                        TaxIDs assigned, the LENgth of the read, the SCOre
                        given to the assignment, and the taxid code used for
                        UNClassified reads
  -f FILE, --file FILE  Centrifuge output files. If a single directory is
                        entered, every .out file inside will be taken as a
                        different sample. Multiple -f is available to include
```

```
                        several Centrifuge samples.
  -g FILE, --generic FILE
                        Output file from a generic classifier. It requires the
                        flag --format (see such option for details). Multiple
                        -g is available to include several generic samples.
  -l FILE, --lmat FILE  LMAT output dir or file prefix. If just "." is
                        entered, every subdirectory under the current
                        directory will be taken as a sample and scanned
                        looking for LMAT output files. Multiple -l is
                        available to include several samples.
  -r FILE, --clark FILE
                        CLARK full-mode output files. If a single directory is
                        entered, every .csv file inside will be taken as a
                        different sample. Multiple -r is available to include
                        several CLARK, CLARK-l, and CLARK-S full-mode samples.
  -k FILE, --kraken FILE
                        Kraken output files. If a single directory is entered,
                        every .krk file inside will be taken as a different
                        sample. Multiple -k is available to include several
                        Kraken (version 1 or 2) samples.
```

5.2.2 Output

Related to the Recentrifuge output files:

```
  -o FILE, --outhtml FILE
                        HTML output file (if not given, the filename will be
                        inferred from input files)
  -e OUTPUT_TYPE, --extra OUTPUT_TYPE
                        type of extra output to be generated, and can be one
                        of ['FULL', 'DYNOMICS', 'CSV', 'TSV']
```

5.2.3 Tuning

Coarse tuning of algorithm parameters:

```
  -c CONTROLS_NUMBER, --controls CONTROLS_NUMBER
                        this number of first samples will be treated as
                        negative controls; default is no controls
  -s SCORING, --scoring SCORING
                        type of scoring to be applied, and can be one of
                        ['SHEL', 'LENGTH', 'LOGLENGTH', 'NORMA', 'LMAT',
                        'CLARK_C', 'CLARK_G', 'KRAKEN', 'GENERIC']
  -y NUMBER, --minscore NUMBER
                        minimum score/confidence of the classification of a
                        read to pass the quality filter; all pass by default
  -m INT, --mintaxa INT
                        minimum taxa to avoid collapsing one level into the
                        parent (if not specified a value will be automatically
                        assigned)
  -x TAXID, --exclude TAXID
                        NCBI taxid code to exclude a taxon and all underneath
                        (multiple -x is available to exclude several taxid)
  -i TAXID, --include TAXID
                        NCBI taxid code to include a taxon and all underneath
                        (multiple -i is available to include several taxid);
                        by default, all the taxa are considered for inclusion
  -a, --avoidcross      avoid cross analysis
```

5.2.4 Fine tuning

Fine tuning of algorithm parameters:

```
  -z NUMBER, --ctrlminscore NUMBER
```

```
                        minimum score/confidence of the classification of a
                        read in control samples to pass the quality filter; it
                        defaults to ”minscore”
  -w INT, --ctrlmintaxa INT
                        minimum taxa to avoid collapsing one level into the
                        parent (if not specified a value will be automatically
                        assigned)
  -u OPTION, --summary OPTION
                        select to ”add” summary samples to other samples, or
                        to ”only” show summary samples or to ”avoid” summaries
                        at all
  -t, --takeoutroot     remove counts directly assigned to the ”root” level
  --nokollapse          show the ”cellular organisms” taxon
```

5.2.5 Advanced

Advanced modes of running:

```
  -d, --debug           increase output verbosity and perform additional
                        checks (default: False)
  --sequential          deactivate parallel processing (default: False)
```

5.2.6 Other

Other useful arguments:

```
  -h, --help            show the help message and exit
  -V, --version         show program’s version number and exit
```

6 Troubleshooting

This section includes topics of interest in case of issues or suspected troubles in the use of the Recentrifuge package.

6.1 Mismatch between NCBI database versions

The mismatch of the versions of the NCBI database between Recentrifuge and the taxonomic classification engine is not usually a big problem, as long as the Recentrifuge database is more recent and the one used by the classifier is not very old.

- If the taxonomic classification engine uses a newer database, Recentrifuge could miss some new taxids (taxonomic identifiers, aka TaxIDs). Please, use `retaxdump` regularly to keep an updated version of the NCBI taxonomic database used by Recentrifuge.
- If the taxonomic classifier uses an old NCBI database, Recentrifuge could miss some obsolete or merged taxonomic identifiers. Please keep the NCBI taxonomic database used by your classification engine updated, since the database is evolving rapidly. If your data is old, you may benefit from reanalyzing your dataset with updated database and software releases.

When parsing your samples, if Recentrifuge detects orphan taxids (those without a known parent in the NCBI taxonomic tree), it issues a warning like:

```
Warning! 4 orphan taxids (rerun with --debug for details)
```

In this case, or if you are unsure of the effects of the NCBI database mismatch between Recentrifuge and your taxonomic classifier, or any other problem related to the taxonomic identifiers, we suggest using the debug flag (**-d** or **--debug**) in `rcf`. The debugging mode enables the taxid-loss tests, which provide details about the number of orphan taxids and the number of orphan sequences (those sequences afflicted by being assigned to an orphan taxid). Bellow is an example of real output for a CLARK-S sample:

```
Checking taxid loss (orphans)...
Warning! Orphan taxid=697046
Warning! Orphan taxid=758602
Warning! Orphan taxid=1870930
Warning! Orphan taxid=2071623
WARNING! 4 orphan taxids (0.25% of accepted)
  and 6 orphan sequences (0.001% of accepted)
```

In this particular case, the first three taxonomic identifiers were merged into other taxids recently, during the previous months. The last one is currently unknown for the NCBI taxonomic database (as of Dec 30, 2018). Those taxids affected a tiny fraction of the reads, but they sometimes could represent organisms critical to the results.

In summary, our recommendation is to keep both databases updated and use the debug flag (**-d**) in Recentrifuge (**rcf**) to clear up doubts.

6.2 Number of total accumulated reads is less than the accepted reads

The number of total accumulated reads (those counts shown for "root" in a sample by Recentrifuge) can be less than the number of accepted reads. Recentrifuge gives information about this situation:

```
Check for more seqs lost ([in/ex]clude affects)...
Info: 902451 additional seqs discarded (83.560% of accepted)
```

On the other hand, if the values are the same, the message is simply:

```
Check for more seqs lost ([in/ex]clude affects)... OK!
```

The main reason for the mismatch between the number of total accumulated reads and accepted reads is the use of exclusion (**-x** option) or inclusion (**-i** option) lists in `rcf`, especially if the number of discarded sequences is high. Another reason could be the (undesirable) presence of orphan taxids (see the previous subsection). In any case, you can get further details running Recentrifuge with the debugging flag **-d** enabled.

6.3 Where have those taxa with scarce reads assigned gone?

In the taxonomic tree built for each sample, a taxon with a number of assigned reads below the threshold of `mintaxa` is "folded" in the parent taxon, which then combines its counts and scores with those of the child. This algorithm is a kind of high-frequency noise filter to prevent background noise, such as the produced by random sequencing errors or misclassification statistical noise. This noise is seen as an overdispersion of reads in the space of the taxids, in other words, an overestimation of the number of taxa present in the samples due to relatively low quantities of reads assigned to many taxids.

If you don't use exclusion (**-x** option) or inclusion (**-i** option) lists in `rcf`, you can get details about the "tree folding" of the taxa placed on the leaves of the taxonomic tree by enabling the debugging flag (**-d** or **--debug**). For example, using the same CLARK example as in subsections above:

```
Assess accumulation due to ”folding the tree”...
Info: Folded taxid 146923 (Streptomyces parvulus) with 1 original seqs
Info: Folded taxid 78258 (Parascardovia denticolens) with 2 original seqs
Info: Folded taxid 28095 (Burkholderia gladioli) with 3 original seqs
Info: Folded taxid 585 (Proteus vulgaris) with 2 original seqs
(...)
Info: Folded TaxID 860 (Fusobacterium periodonticum) with 1 original seqs
Info: Folded TaxID 2005460 (Chondrocystis sp. NIES-4102) with 1 original seqs
Info: Folded TaxID 194424 (Sulfurospirillum halorespirans) with 1 original seqs
INFO: 1313 TaxIDs folded (83.31% of TAF —TaxIDs after filtering—)
INFO: Final assigned TaxIDs: 579 (reduced to 36.74% of number of TAF)
```

So, there were a lot of taxids with a low number of assigned reads, in other words, a lot of dispersion of scarce reads in taxids. The 83.31% of the TaxIDs with reads assigned after filtering were "folded," so their reads were accumulated into their parents and so forth, until the `mintaxa` threshold was satisfied. Finally, the number of TaxIDs with reads assigned was reduced to a 36.74% of the number of TaxIDs after filtering (a value that could be lower than the initial number of TaxIDs in the sample because of the score filter applied when parsing the sample data).

6.4 Issues with LMAT plasmids

If you add the file `plasmid.names.txt` to the `taxdump` directory as detailed in S4 Appendix Section 1.2.2 in order to include LMAT plasmids classifications in your results, Recentrifuge will detect and parse it. Typically, Recentrifuge performs a sanity check for inconsistencies in the plasmid data and outputs a summary with the relevant information. If you have issues with the LMAT plasmids, please enable the debugging flag is active (**-d**) to `rcf`, since then Recentrifuge will give details. Please see the S4 Appendix Section 3.2.4 for more information.

List of Figures

1 Retest flowchart diagram 18

2 ROC generated by retest showing the impact of the robust contamination removal 19

3 ROC generated by retest in order to study the dependence on the mintaxa parameter . . . 19

4 The mock community 20

References

1. González R, House P, Levkivskyi I, Roach L, Rosum GV (2016) PEP-526: Syntax for Variable Annotations. [Internet]. Python Software Foundation. URL: https://www.python.org/dev/peps/pep-0526/.
2. Smith EV (2015) PEP-498: Literal String Interpolation. [Internet]. Python Software Foundation. URL: https://www.python.org/dev/peps/pep-0498/.
3. Rosum GV, Lehtosalo J, Langa L (2015) PEP-484: Type Hints. [Internet]. Python Software Foundation. URL: https://www.python.org/dev/peps/pep-0484/.
4. Sczyrba A, Hofmann P, Belmann P, Koslicki D, Janssen S, Dröge J, Gregor I, Majda S, Fiedler J, Dahms E, Bremges A, Fritz A, Garrido-Oter R, Jørgensen TS, Shapiro N, Blood PD, Gurevich A, Bai Y, Turaev D, DeMaere MZ, Chikhi R, Nagarajan N, Quince C, Meyer F, Balvočiūtė M, Hansen LH, J. SS, Chia BKH, Denis B, Froula JL, Wang Z, Egan R, Kang DD, Cook JJ, Deltel C, Beckstette M, Lemaitre C, Peterlongo P, Rizk G, Lavenier D, Wu YW, Singer SW, Jain C, Strous M, Klingenberg H, Meinicke P, Barton MD, Lingner T, Lin HH, Liao YC, Silva GGZ, Cuevas DA, Edwards RA, Saha S, Piro VC, Renard BY, Pop M, Klenk HP, Göker M, Kyrpides NC, Woyke T, Vorholt JA, Schulze-Lefert P, Rubin EM, Darling AE, Rattei T, McHardy AC (2017) Critical Assessment of Metagenome Interpretation—a benchmark of metagenomics software. Nature Methods 14: 1063–1072. DOI: 10.1038/nmeth.4458.
5. Burguet-Castell J, Martí JM (2015) Entrez: Call the NCBI E-utilities from Python. Version 2018. URL: https://github.com/jordibc/entrez.
